# A New Brachylophosaurin Hadrosaur (Dinosauria: Ornithischia) with an Intermediate Nasal Crest from the Campanian Judith River Formation of Northcentral Montana

**DOI:** 10.1371/journal.pone.0141304

**Published:** 2015-11-11

**Authors:** Elizabeth A. Freedman Fowler, John R. Horner

**Affiliations:** 1 Museum of the Rockies, Montana State University, Bozeman, Montana, United States of America; 2 Department of Earth Sciences, Montana State University, Bozeman, Montana, United States of America; University of Oxford, UNITED KINGDOM

## Abstract

**Background:**

Brachylophosaurini is a clade of hadrosaurine dinosaurs currently known from the Campanian (Late Cretaceous) of North America. Its members include: *Acristavus gagslarsoni*, which lacks a nasal crest; *Brachylophosaurus canadensis*, which possesses a flat paddle-shaped nasal crest projecting posteriorly over the dorsal skull roof; and *Maiasaura peeblesorum*, which possesses a dorsally-projecting nasofrontal crest. *Acristavus*, from the lower Two Medicine Formation of Montana (~81–80 Ma), is hypothesized to be the ancestral member of the clade. *Brachylophosaurus* specimens are from the middle Oldman Formation of Alberta and equivalent beds in the Judith River Formation of Montana; the upper Oldman Formation is dated 77.8 Ma.

**Methodology/Principal Findings:**

A new brachylophosaurin hadrosaur, *Probrachylophosaurus bergei* (gen. et sp. nov.) is described and phylogenetically analyzed based on the skull and postcranium of a large individual from the Judith River Formation of northcentral Montana (79.8–79.5 Ma); the horizon is equivalent to the lower Oldman Formation of Alberta. Cranial morphology of *Probrachylophosaurus*, most notably the nasal crest, is intermediate between *Acristavus* and *Brachylophosaurus*. In *Brachylophosaurus*, the nasal crest lengthens and flattens ontogenetically, covering the supratemporal fenestrae in large adults. The smaller nasal crest of *Probrachylophosaurus* is strongly triangular in cross section and only minimally overhangs the supratemporal fenestrae, similar to an ontogenetically earlier stage of *Brachylophosaurus*. Sutural fusion and tibial osteohistology reveal that the holotype of *Probrachylophosaurus* was relatively more mature than a similarly large *Brachylophosaurus* specimen; thus, *Probrachylophosaurus* is not simply an immature *Brachylophosaurus*.

**Conclusions/Significance:**

The small triangular posteriorly oriented nasal crest of *Probrachylophosaurus* is proposed to represent a transitional nasal morphology between that of a non-crested ancestor such as *Acristavus* and the large flat posteriorly oriented nasal crest of adult *Brachylophosaurus*. Because *Probrachylophosaurus* is stratigraphically and morphologically intermediate between these taxa, *Probrachylophosaurus* is hypothesized to be an intermediate member of the *Acristavus*-*Brachylophosaurus* evolutionary lineage.

## Introduction

Hadrosaurid dinosaurs were prominent members of Late Cretaceous Campanian ecosystems of North America, with an abundance of diverse taxa. The later portion of the Campanian included solid-crested hadrosaurines coexisting with hollow-crested lambeosaurines, but earlier in the Campanian, hadrosaurines were the most abundant hadrosaurids (See Materials and Methods section regarding use of the term “hadrosaurine”. Extensive bonebeds in the Two Medicine and Judith River Formations of Montana preserve numerous individuals of hadrosaurine taxa such as *Maiasaura peeblesorum* and *Brachylophosaurus canadensis* [1, 2]. These genera are members of the clade Brachylophosaurini, which includes the recently described basal taxon *Acristavus gagslarsoni* [3].

**Fig 1:**
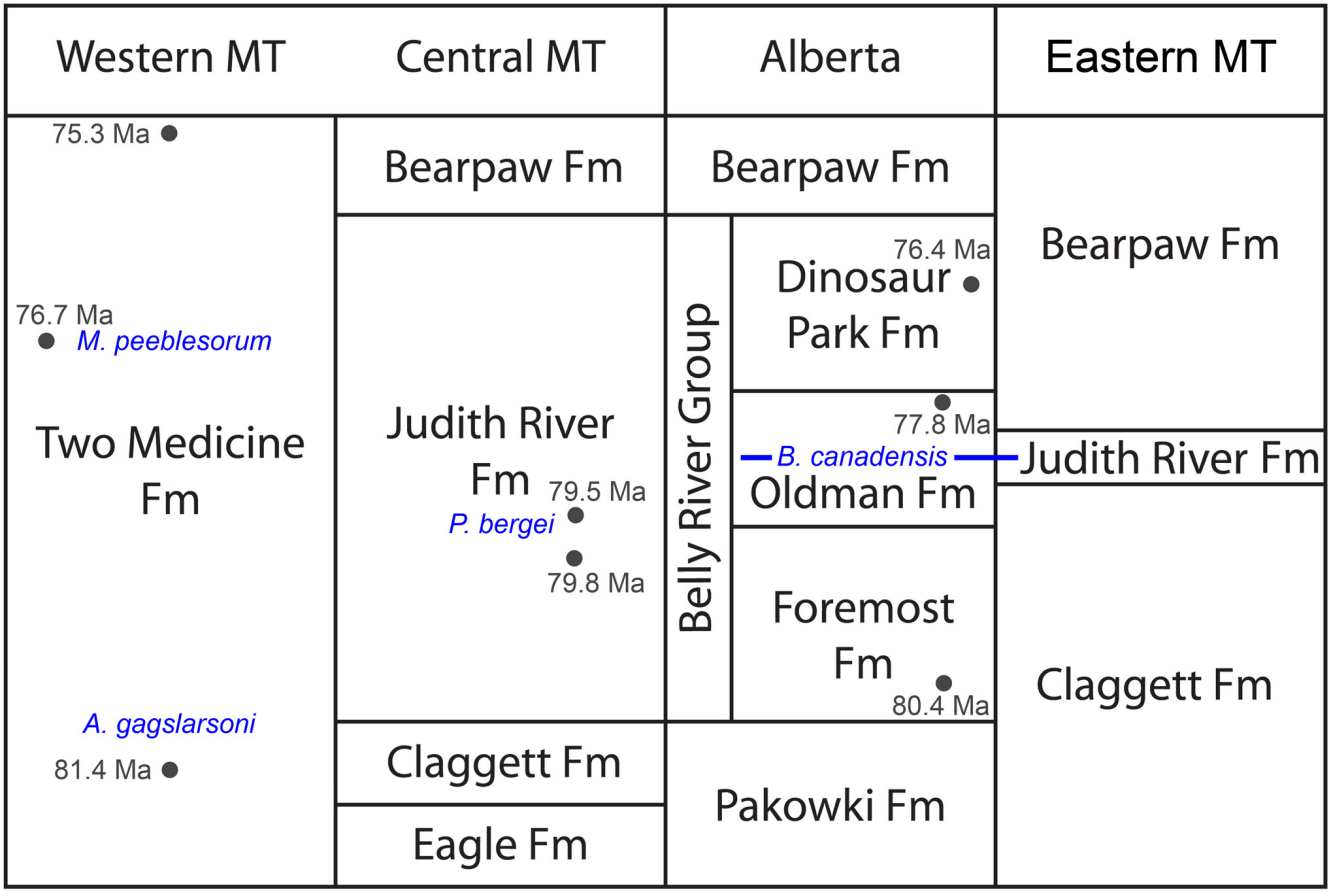
Generalized regional cross section of the Judith River Formation and stratigraphic equivalents, with brachylophosaurin distribution. Members of Brachylophosaurini indicated in blue: *Acristavus gagslarsoni*, *Probrachylophosaurus bergei* gen. et sp. nov., *Brachylophosaurus canadensis*, and *Maiasaura peeblesorum*. Radiometric dates are indicated in dark gray, and have been recalibrated to the Fish Canyon sanidine standard (28.305 +/- 0.036 Ma) of Renne et al. [10] from the originally published values [11–15]; see text and Table 1 for further recalibration details.

*Acristavus*, named for its lack of nasal crest, is found in the lower Two Medicine Formation of Montana (Fig 1; [3]). *Maiasaura* and *Brachylophosaurus* are more typical hadrosaurines in having prominent nasal crests, and are found stratigraphically higher than *Acristavus*. *Maiasaura*, with its dorsally projecting nasofrontal crest, is known from the upper half of the Two Medicine Formation [4–6]. *Brachylophosaurus canadensis* specimens have posteriorly oriented flat paddle-shaped nasal crests, and are likely all from the Comrey Sandstone Zone of the middle Oldman Formation of Alberta and its Judith River Formation equivalent in Montana [7, 8]. A previously published taxon from lower Oldman-equivalent deposits, “*Brachylophosaurus goodwini*” [9], has been referred to *Brachylophosaurus canadensis* [1] and is here considered referable only to Brachylophosaurini indet. due to the state of preservation of the holotype and the lack of a preserved nasal crest.

Stratigraphically intermediate deposits between *Acristavus* and *Brachylophosaurus* include the lower Judith River Formation of Montana, and the corresponding Foremost and lower Oldman Formations of Alberta. Although several partial skeletons have been excavated from the Foremost and lower Oldman Formations and their Judith River Formation equivalents, no species-level diagnostic hadrosaurid material has previously been collected near the Foremost-Oldman Formation boundary, resulting in a gap in our knowledge of Campanian hadrosaur evolution. This paper describes MOR 2919, a specimen with a relatively complete skull from this stratigraphic interval in Kennedy Coulee, northcentral Montana, which fills in the gap with a transitional morphology between the known taxa *Acristavus gagslarsoni* and *Brachylophosaurus canadensis*.

## Institutional Abbreviations

CMN/NMC, Canadian Museum of Nature, formerly National Museum of Canada, Ottawa, Ontario, Canada; FMNH, Field Museum of Natural History, Chicago, Illinois, U.S.A.; GPDM, Great Plains Dinosaur Museum, Malta, Montana, U.S.A.; OTM, Old Trail Museum, Choteau, Montana, U.S.A.; MOR, Museum of the Rockies, Bozeman, Montana, U.S.A.; ROM, Royal Ontario Museum, Toronto, Ontario, Canada; TMP, Royal Tyrrell Museum of Palaeontology, Drumheller, Alberta, Canada; UCMP, University of California Museum of Paleontology, Berkeley, California, U.S.A.; UMNHVP, Natural History Museum of Utah Vertebrate Paleontology, Salt Lake City, Utah, U.S.A.; YPM-PU, Princeton University collection at the Yale Peabody Museum, New Haven, Connecticut, U.S.A.

## Study Areas

**Fig 2:**
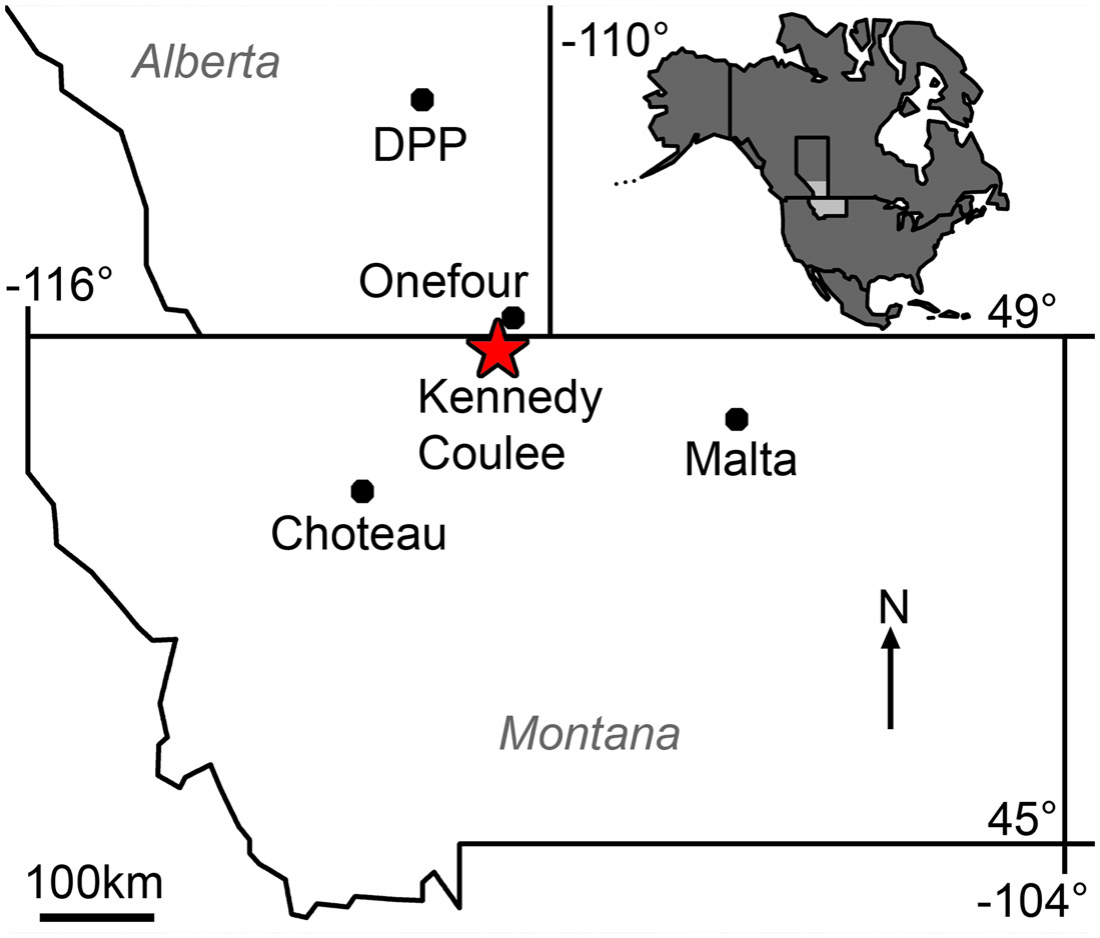
Map of Brachylophosaurini localities in Montana and southern Alberta. Inset shows North America in dark grey with Montana and southern Alberta in light grey. Kennedy Coulee, locality of *Probrachylophosaurus bergei* gen. et sp. nov., marked with red star. Other Brachylophosaurini localities indicated with black dots: Malta, *Brachylophosaurus canadensis*; Onefour, *B*. *canadensis*; DPP (Dinosaur Provincial Park), *B*. *canadensis*; Choteau, *Acristavus gagslarsoni* and *Maiasaura peeblesorum*.

Kennedy Coulee is a richly fossiliferous exposure of the Judith River Formation (Late Cretaceous: Campanian) located in Hill County, northcentral Montana, and joins the valley of the Milk River near the USA-Canada border (Fig 2). In Alberta, Judith River Formation equivalent deposits are named the Belly River Group, and include the Foremost, Oldman, and Dinosaur Park Formations (Fig 1). Although the Montanan Judith River Formation is not subdivided into the formal units of the Belly River Group, the depositional sequences that created the Foremost, Oldman, and Dinosaur Park Formations can be identified in Montana outcrop. Using the terminology of these Canadian formations and their members enables detailed stratigraphic placement of fossiliferous localities, facilitating comparison of the hadrosaurids and other fauna over time.

Exposures in and near Kennedy Coulee are equivalent to the uppermost Foremost Formation and lowermost Oldman Formation of Alberta, according to multiple lines of evidence: lithologic, radiometric, and biostratigraphic. The base of the coulee exposes a thick coal, the Marker A Coal, equivalent to the top of the Taber Coal Zone of the Foremost Formation, capped by a white-gray amalgamated channel sandstone equivalent to the Herronton Sandstone Zone at the top of the Foremost Formation (sensu [13]). The upper portion of Kennedy Coulee, where MOR 2919 was collected, is dominated by mudstones and corresponds to Unit 1 (the lowest unit) of the Oldman Formation. The ceratopsian *Medusaceratops* was also collected from this upper mudstone zone of Kennedy Coulee [16].

To facilitate comparison of radiometric dates from various formations and publications, all relevant dates were recalibrated to the Fish Canyon sanidine standard of 28.305 ± 0.036 Ma using the method of Renne et al. [10]. Published and recalibrated dates for relevant portions of the Judith River Formation, Belly River Group, Two Medicine Formation, and Wahweap Formation are listed in Table 1, and recalibrated dates are plotted on Fig 1.

**Table 1:** Radiometric date recalibrations.

Formation	Stratigraphic height	Published			Recalibrated			Source Publication	Notes
		FCs age	Age	Error	FCs age	Age	Error		
Judith River	Unit 1 of Oldman Formation, 31.8 m above top of Marker A Coal of Taber Coal Zone	27.84*	78.2	0.2	28.305	79.49	0.21	Goodwin and Deino, 1989	Upper Kennedy Coulee, equivalent to Unit 1 of Oldman Formation. Sanidine. *Original standard used was MMhb-1 = 520.4 +/- 1.7 Ma, which was equivalent to FCs = 27.84 (Renne et al., 1998).
Judith River	Taber Coal Zone of Foremost Formation, 4.8 m below top of Marker A Coal	27.84*	78.5	0.2	28.305	79.79	0.21	Goodwin and Deino, 1989	Lower Kennedy Coulee, equivalent to Taber Coal Zone of Foremost Formation. Sanidine. *Original standard used was MMhb-1 = 520.4 +/- 1.7 Ma, which was equivalent to FCs = 27.84 (Renne et al., 1998).
Dinosaur Park	middle, 44 m above bottom	27.84*	75.2	0.3	28.305	76.45	0.31	Eberth and Hamblin, 1993	Sample from Dinosaur Provincial Park area. *The standard used is not mentioned in Eberth and Hamblin (1993), but the paper cites the methods of Thomas et al. (1990), which used the standard of MMhb-1 = 520.4 +/- 1.7 Ma, which was equivalent to FCs = 27.84 (Renne et al., 1998).
Oldman	upper, 4 m below top	27.84*	76.5	0.5	28.305	77.76	0.51	Eberth and Hamblin, 1993	Sample from Dinosaur Provincial Park area. *The standard used is not mentioned in Eberth and Hamblin (1993), but the paper cites the methods of Thomas et al. (1990), which used the standard of MMhb-1 = 520.4 +/- 1.7 Ma, which was equivalent to FCs = 27.84 (Renne et al., 1998).
Foremost	lower, 30 m above bottom	27.84*	79.14	0.15	28.305	80.45	0.16	Eberth, 2005 citing A. L. Deino personal communication, 1993	Sample from Dinosaur Provincial Park area. *The standard used is not mentioned in Eberth (2005), but in the early 1990s Deino’s lab, part of the Berkeley Geochronology Center, was using the Fish Canyon sanidine standard of 27.84 Ma (P. R. Renne personal communication, 2011). The samples listed above were tested in the same lab using the same standard.
Two Medicine	upper, 10 m below top	27.84	74.08	0.05	28.305	75.30	0.07	Rogers et al., 1993	Sample TM-6 plagioclase.
Two Medicine	upper middle	28.03	75.92	0.32	28.305	76.66	0.32	Varricchio et al., 2010	Bentonite is associated with the MOR TM-003 *Maiasaura* bonebed.
Two Medicine	lower, 100 m above bottom	27.84	80.04	0.08	28.305	81.37	0.10	Rogers et al., 1993	Sample RT/TM-7 biotite. This original date was used to estimate age of ~79.43 Ma for MOR 1155, holotype of *Acristavus gagslarsoni* (Gates et al., 2011), which recalibrates to 80.74 Ma.
Wahweap	middle, 40 m above bottom	28.02	80.1	0.3	28.305	80.91	0.31	Jinnah et al., 2009	Sample CF05-B bentonite sanidine. This original date was used to estimate age of 79.34–78.91 Ma for UMNHVP 16607, referred specimen of *Acristavus gagslarsoni* (Gates et al., 2011), which recalibrates to 80.14–79.71 Ma.

Published dates have been recalibrated to the Fish Canyon sanidine standard (28.305 +/- 0.036 Ma) of Renne et al. [10]. Unless specified, stratigraphic heights "above bottom" and "below top" refer to the bottom and top of the entire formation, not its members. Ages and errors are in Ma. FCs: Fish Canyon sanidine standard used for published ages. MMhb-1: McClure Mountain hornblende standard. References used in Table 1: [11–15, 17–19].

Published radiometric dates from the upper mudstone zone and lower coal zone of Kennedy Coulee constrain the age of MOR 2919 between 78.5 +/- 0.2 Ma and 78.2 +/- 0.2 Ma according to the old standard of 520.4 +/- 1.7 Ma for MMhb-1 [11]. Conversion and recalibration to the Fish Canyon sanidine standard mentioned above adjusts these dates to 79.8 +/- 0.2 Ma and 79.5 +/- 0.2 Ma. The proposed stratigraphic position of Kennedy Coulee is consistent with its radiometric age within the range of recalibrated dates from the lower Foremost (80.45 +/- 0.16 Ma) and upper Oldman Formations (77.76 +/- 0.51 Ma) of Alberta [12, 13].

Ray teeth collected from microsites throughout Kennedy Coulee, from the Herronton Sandstone to Unit 1 of the Oldman Formation, possess smooth-sided crowns, and are thus referable to *Pseudomyledaphus* sp. (S1 Text, S1 Fig) [20]. In Alberta, *Pseudomyledaphus* sp. are only found in the Foremost and lowermost Oldman Formations [20–22], supporting the assignment of MOR 2919’s locality to the lowermost Oldman Formation.

*Brachylophosaurus canadensis* specimens collected in Alberta all likely originate from the Oldman Formation (Table 2). The holotype of *B*. *canadensis*, CMN 8893, was collected in 1936 from the Comrey Sandstone Zone (Unit 2) of the middle Oldman Formation near the Red Deer River, within what is now Dinosaur Provincial Park ([23]; D. A. Eberth personal communication 2011). A partial skull, FMNH PR 862, had also been collected in Dinosaur Provincial Park in 1922 [24]. FMNH PR 862 was more recently erroneously associated with the Two Medicine Formation [1, 25], but outcrops along the Red Deer River include the Oldman and Dinosaur Park Formations, and so FMNH PR 862 was likely collected from a similar stratigraphic level as the holotype. A third skull and partial skeleton, TMP 1990.104.001, was collected from a sandstone at an unspecified level of the Oldman Formation near the Milk River outside of the towns of Onefour and Manyberries [7]. Of these three specimens, only the holotype is confirmed to be from the Comrey Sandstone Zone, but there is no evidence to suggest that the other two specimens originate from a different horizon.

**Table 2:** List of Brachylophosaurini specimens examined in this paper.

Taxon	Specimen	Type	Material Preserved	Ontogenetic Stage	Stratigraphic Position	Recalibrated Age	Specimen References
*Probrachylophosaurus bergei*	MOR 2919	holotype	skull (partially articulated) and partial skeleton	adult	Judith River Fm equivalent to Unit 1 of Oldman Fm, 17.5 m above top of Taber Coal Zone of Foremost Fm	78.5–78.2 Ma	this paper
*Probrachylophosaurus bergei*	MOR 1097	referred	partial skull (disarticulated)	subadult	Judith River Fm equivalent to Unit 1 of Oldman Fm	78.5–78.2 Ma	this paper
*Brachylophosaurus canadensis*	CMN 8893	holotype	skull (articulated) and partial skeleton	adult	Unit 2 (Comrey Sandstone) of Oldman Fm, 7.5 m below top of fm	> 77.76 Ma	Sternberg 1953, Cuthbertson and Holmes 2010
*Brachylophosaurus canadensis*	GPDM JRF.65	referred	braincase	adult	Judith River Fm equivalent to Unit 2 (Comrey Sandstone) of Oldman Fm	> 77.76 Ma	
*Brachylophosaurus canadensis*	FMNH PR 862	referred	skull (partially articulated)	adult	possibly Oldman Fm (quarry not relocated)	unknown	
*Brachylophosaurus canadensis*	MOR 720	referred	braincase	adult	upper Judith River Fm	unknown	
*Brachylophosaurus canadensis*	MOR 794	referred	skull (articulated) and skeleton (articulated)	adult	Judith River Fm equivalent to Unit 2 (Comrey Sandstone) of Oldman Fm	> 77.76 Ma	Prieto-Márquez 2005
*Brachylophosaurus canadensis*	MOR 940	referred	braincase	subadult	Judith River Fm equivalent to Unit 2 (Comrey Sandstone) of Oldman Fm	> 77.76 Ma	
*Brachylophosaurus canadensis*	MOR 1071	referred	bonebed including skulls (partially articulated and disarticulated) and skeletons	adults, subadults, juveniles	Judith River Fm equivalent to Unit 2 (Comrey Sandstone) of Oldman Fm	> 77.76 Ma	Prieto-Márquez 2005
*Brachylophosaurus canadensis*	TMP 1990.104.001	referred	skull (articulated) and partial skeleton	adult	Oldman Fm	unknown	Cuthbertson and Holmes 2010
*Maiasaura peeblesorum*	YPM-PU 22405	holotype	skull (partially articulated)	adult	upper Two Medicine Fm	~76.66 Ma	Horner and Makela 1979, Horner 1983
*Maiasaura peeblesorum*	OTM F138	referred	skull (partially articulated) and skeleton	adult	Two Medicine Fm	unknown	Trexler 1995
*Maiasaura peeblesorum*	ROM 44770	referred	skull (articulated) and partial skeleton	adult	Two Medicine Fm	unknown	
*Acristavus gagslarsoni*	MOR 1155	holotype	skull (mostly articulated) and partial skeleton	adult?	lower Two Medicine Fm	~80.74 Ma	Gates et al. 2011
*Acristavus* sp.	UMNHVP 16607	referred	braincase	adult?	upper Middle Mudstone Member of Wahweap Fm, 170 m above base of fm	80.14–79.71 Ma	Gates et al. 2011
Brachylophosaurini indet. "*Brachylophosaurus goodwini*"	UCMP 130139	holotype	partial skull (partially articulated) and partial skeleton	adult	Judith River Fm equivalent to Unit 1 of Oldman Fm	78.5–78.2 Ma	Horner 1988

Relative ontogenetic stages are estimated using the size-based definitions of Evans [26]; size alone should not be used to infer the sexual or skeletal maturity or immaturity of a specimen. Juveniles are less than 50% the size of the largest known specimen of that taxon; subadults are 50–85% the size of the largest specimen; adults are more than 85% the size of the largest specimen. For details and references of the recalibrated ages, see Table 1. *Abbreviations*: *Fm*, Formation.

Although Alberta was the source of the holotype, the majority of known *Brachylophosaurus canadensis* specimens, including a complete adult skeleton (MOR 794), an articulated subadult with skin impressions (JRF 115), and a bonebed containing individuals of various sizes (MOR 1071), were collected from the Judith River Formation exposed near Malta, Montana. Malta is 200 km east of Kennedy Coulee, and thus more distal along the depositional wedge and closer to the Western Interior Seaway. In the Malta area, the base of the Judith River Formation is the shoreface Parkman Sandstone, which is overlain by tan colored, quartz-rich sandstones [8] equivalent to the Comrey Sandstone Zone of the middle Oldman Formation. Strata equivalent to the Foremost Formation and lower Oldman Formation are not present in the distal wedge deposits of Malta (Fig 1). Ray teeth collected with MOR 1071 are referable to *Myledaphus bipartitus* (S1 Text), which, in Alberta, are only found in the Comrey Sandstone Zone and higher units [21], supporting the assignment of the Malta *B*. *canadensis* localities to the Comrey Sandstone Zone of the Oldman Formation. Thus, Kennedy Coulee and MOR 2919 are stratigraphically older than *Brachylophosaurus canadensis* specimens from Alberta and Malta, Montana.

A second species of *Brachylophosaurus*, *B*. *goodwini*, was described from Kennedy Coulee, Hill County, northcentral Montana [9]. The cranial material of the holotype, UCMP 130139, was fragmentary and missing the nasal crest, and so was deemed undiagnostic and referred to *B*. *canadensis* [1, 23]. However, due to the lack of a preserved nasal crest as well as its severe frontal depressions, UCMP 130139 cannot be confidently referred to either *B*. *canadensis* or the new genus described here, and is assigned to Brachylophosaurini indet. (see Discussion).

## Materials and Methods

MOR 2919 was collected by Museum of the Rockies and University of California Museum of Paleontology crews using standard paleontology field techniques [27] on privately owned land. MOR 2919 was generously donated to the Museum of the Rockies by Nolan and Cheryl Fladstol and John and Claire Wendland. No permits were required for the described study, which complied with all relevant regulations.

Terminology in this paper follows traditional conventions and definitions of Hadrosauridae and Hadrosaurinae [28] rather than the proposed alternate names of Saurolophidae and Saurolophinae [29] for reasons of taxonomic stability as detailed in Gates et al. [3].

MOR 2919 was compared morphologically to other hadrosaurine specimens and casts at MOR, ROM, TMP, and UCMP, as well as published descriptions. Measurements were taken directly from the specimens or casts using a tape measure or digital calipers.

To determine the phylogenetic relationships of MOR 2919 within Hadrosaurinae, the specimen was coded into the phylogenetic matrices of Gates et al. [3] and Prieto-Márquez [29]. These matrices were selected because Gates et al. [3] is the description of *Acristavus*, one of the taxa most morphologically similar to MOR 2919, and Prieto-Márquez [29] is a landmark study, the largest, most comprehensive analysis of all Hadrosauridae to that date. The matrices from both studies were used, because although they both have origins in the Horner et al. [28] matrix, the Gates et al. [3] matrix alters few characters, whereas the Prieto-Márquez [29] matrix substantially expands the character list, yielding two extremely different matrices. If both matrices yield the same placement of MOR 2919, then its phylogienetic position can be considered very well supported.

To simplify the analyses and focus on relationships within the clade of interest (Brachylophosaurini), as well as to make the taxon lists of these matrices as similar as possible, most lambeosaurine and basal hadrosauroid taxa were removed from Prieto-Márquez’s [29] and Gates et al.’s [3] matrices. Relationships within lambeosaurines and basal hadrosauroids are thoroughly investigated in Prieto-Márquez [29] and need not be repeated here. *Iguanodon bernissartensis* was retained for use as the outgroup, consistent with Prieto-Márquez [29] and Gates et al. [3]. *Bactrosaurus johnsoni* was retained as a representative hadrosauroid because it is the most complete hadrosauroid in the matrices. *Hadrosaurus foulkii*, although not included in the Gates et al. [3] matrix, was retained in the Prieto-Márquez [29] matrix due to the taxon’s variable position in different phylogenetic analyses and its importance in defining the terms Hadrosauridae and Hadrosaurinae. *Corythosaurus casuarius* was retained as a representative lambeosaurine, consistent with Gates et al. [3]. Several incomplete hadrosaurine taxa (coded with a high percentage of “?”) outside of Brachylophosaurini were removed from both matrices to simplify the analysis and improve resolution of Brachylophosaurini and the other major, well-defined hadrosaurine clades. The removed taxa were: *Barsboldia sicinskii*, *Kerberosaurus manakini*, Sabinas OTU, Salitral Moreno OTU (*Willinakaqe*), *Shantungosaurus giganteus*, and UTEP OTU.

Several character states in the published Prieto-Márquez [29] matrix were recoded, and after initial analysis, some characters were amended or excluded in a second analysis (see Phylogenetic Analyses section). No character states were recoded in the Gates et al. [3] matrix, and no characters were excluded. Thus, phylogenetic analyses were performed on a total of three matrices: 1) the matrix of Prieto-Márquez [29] with all characters included (19 taxa, 370 characters; S1 File); 2) the matrix of Prieto-Márquez [29] with some characters amended or excluded (19 taxa, 367 characters; S2 File); 3) the matrix of Gates et al. [3] with all characters included (13 taxa, 116 characters; S3 File).

The matrices were analyzed with parsimony in PAUP 4.0b10 [30] within a heuristic search of 5,000 replicates using ACCTRAN optimization and tree bisection-reconnection swapping to produce the most parsimonious trees, which were then combined into a strict consensus tree, and followed by a bootstrap analysis using a heuristic search of 5,000 replicates. The complete PAUP settings used are provided in the nexus files (S1–S3 Files). For the Gates et al. [3] matrix, the bootstrap replicates were increased to 50,000 due to the support values for certain clades being extremely close to the cutoff value of 50%. Phylogeny figures were time-calibrated by drawing the cladograms using published age ranges (in Ma) for Cretaceous taxa; dates for members of Brachylophosaurini were recalibrated as in Table 1 and Fig 1, using the method of Renne et al. [10].

The left tibia of MOR 2919 was histologically sampled using the techniques of Lamm [31] for large specimens. The mid-diaphyseal segment with minimum circumference was removed, molded and cast, and embedded in resin for histological sectioning. Given the large dimensions of the tibia (anteroposterior mid-diaphysis cross-sectional diameter maximum 13.35 cm, mediolateral diameter minimum 10.60 cm), the transverse cross-section was cut into three parts (anterior, posteromedial, and posterolateral) that were mounted on separate slides and ground to a thickness of 100 μm. The finished slides were imaged at 10x and 40x total magnification on a Nikon Optiphot-Pol polarizing microscope with a Nikon DS-Fi1 digital sight camera utilizing an automated stage to move the slide incrementally. The resulting photomicrographs were compiled with NIS-Elements BR 3.0 software into high-resolution TIFF image files. Adobe Photoshop CS2 was used to combine the images of the three slides and trace the circumference of each line of arrested growth (LAG). These circumferences were then measured with ImageJ 1.46r [32].

Because the left tibia was slightly crushed, several cortical segments were displaced radially inward, resulting in the LAG tracings overestimating the true circumference due to the LAGs on either side of a displacement needing to be connected by a radially oriented line. This was corrected for each LAG individually by subtracting the length of these radial lines at the three areas of greatest displacement (anterolateral, posterolateral, posteromedial) from the total LAG circumference measured in ImageJ.

The left tibia of MOR 2919 was originally collected by UCMP in 1981 and curated as UCMP 137272. Limb bones prepared at UCMP at the time sometimes included a metal rebar rod and epoxy inserted into the medullary cavity for rigid support. After the tibia was transferred to MOR collections and sectioned for histology, the rebar was cut out of the tibia segments prior to mounting them on slides to avoid grinding metal on histological equipment. This created the open space seen in the center of the medullary cavity on the finished slides.

### Nomenclatural Acts

The electronic edition of this article conforms to the requirements of the amended International Code of Zoological Nomenclature, and hence the new names contained herein are available under that Code from the electronic edition of this article. This published work and the nomenclatural acts it contains have been registered in ZooBank, the online registration system for the ICZN. The ZooBank LSIDs (Life Science Identifiers) can be resolved and the associated information viewed through any standard web browser by appending the LSID to the prefix "http://zoobank.org/". The LSID for this publication is: urn:lsid:zoobank.org:pub:657DD0A4-799D-443A-BE9F-A2987DFABADD. The electronic edition of this work was published in a journal with an ISSN, and has been archived and is available from the following digital repositories: PubMed Central, LOCKSS.

## Results

### Systematic Paleontology

Dinosauria Owen, 1842

Ornithischia Seeley, 1888

Ornithopoda Marsh, 1881

Hadrosauridae Cope, 1869

Hadrosaurinae Cope, 1869

Brachylophosaurini Gates et al., 2011

#### Definition

Modified from Gates et al. [3]: Hadrosaurine ornithopods more closely related to *Brachylophosaurus*, *Probrachylophosaurus*, *Maiasaura*, or *Acristavus* than to *Gryposaurus* or *Saurolophus*.

#### Diagnosis

As in Gates et al. [3].

#### Referred material

UCMP 130139, a partial skull and skeleton originally described as the holotype of *Brachylophosaurus goodwini* [9], and later assigned to *Brachylophosaurus canadensis* [1, 23]. Due to the lack of a preserved nasal, and the presence of deep frontal depressions, the specimen cannot be confidently assigned to any current genus of Brachylophosaurini.

#### Horizon and locality

UCMP 130139 was collected from the Judith River Formation of Kennedy Coulee, Hill County, northcentral Montana, in beds equivalent to the lower Oldman Formation, with a published height of approximately 15 m above the Marker A Coal of the Taber Coal Zone of the Foremost Formation [9]. However, a remeasured section shows that the site was actually only a few meters above the Marker A Coal, and lies within the Herronton Sandstone Zone (Mark Goodwin and David Evans personal communication, 2014).

*Brachylophosaurus canadensis* Sternberg, 1953

#### Holotype

CMN 8893

#### Referred Material

FMNH PR 862 (partial skull); MOR 720 (braincase); MOR 794 (nearly complete articulated skeleton); MOR 940 (braincase); MOR 1071 (monodominant bonebed); TMP 90.104.01 (complete skull and articulated partial skeleton).

#### Emended diagnosis (revised from Cuthbertson and Holmes [23])

Nasal crest flat and paddle-shaped in adults, covering most or all of the supratemporal fenestrae; prefrontal as in Cuthbertson and Holmes [23]: “prefrontal projecting posteriorly over frontal, and more posteriorly, ventromedially directed to underlie nasal crest and contribute to anterior border of supratemporal fenestra”. These autapomorphies, together with the following traits, form a unique combination of characters: “only the anterior tip of the lacrimal contacting the maxilla; extremely elongated anterior maxillary process” [23].

#### Remarks

In their rediagnosis of the holotype CMN 8893, Cuthbertson and Holmes [23] reduce the number of autapomorphies listed in Prieto-Márquez [1], and list an additional autapomorphy: “quadratojugal with ‘noncrescentic’ posterior margin variably forming paraquadratic foramen with quadrate” [23]. Because the posterior margin of the quadratojugal and the interpreted presence of a paraquadratic foramen are variable within *Brachylophosaurus* (see Quadrate and Quadratojugal descriptions and comparisons below), this character is not here considered an autapomorphy, and has been excluded from the emended diagnosis.

#### Horizons and localities

CMN 8893, FMNH PR 862, and TMP 90.104.01 were collected from the Oldman Formation of southeastern Alberta. CMN 8893 and FMNH PR 862 were collected in Dinosaur Provincial Park; TMP 90.104.01 was collected near Onefour and the Milk River. Of these Albertan specimens, the exact stratigraphic position is known only for CMN 8893: the Comrey Sandstone Zone (Unit 2) of the Oldman Formation. MOR 720 was collected from the upper Judith River Formation in badlands surrounding the Missouri River north of Winifred, Fergus County, central Montana. MOR 794, MOR 940, and MOR 1071 were collected from the Judith River Formation of Malta, northern Montana, in beds equivalent to the Comrey Sandstone Zone of the Oldman Formation.

*Probrachylophosaurus* gen. nov.

urn:lsid:zoobank.org:act:7B7C87AC-2EFE-4587-9A24-A5D48C908941

#### Type species

*Probrachylophosaurus bergei* sp. nov.

#### Etymology

*Pro-* (Latin) before, -*brachylophosaurus* (Greek) short-crested lizard, in reference to the new taxon’s stratigraphic position below that of *Brachylophosaurus canadensis*.

#### Diagnosis

As for type and only species.

*Probrachylophosaurus bergei* sp. nov.

urn:lsid:zoobank.org:act:49D503CB-7FA6-4D66-8FC0-0B4E2A3EE106

#### Holotype

**Fig 3:**
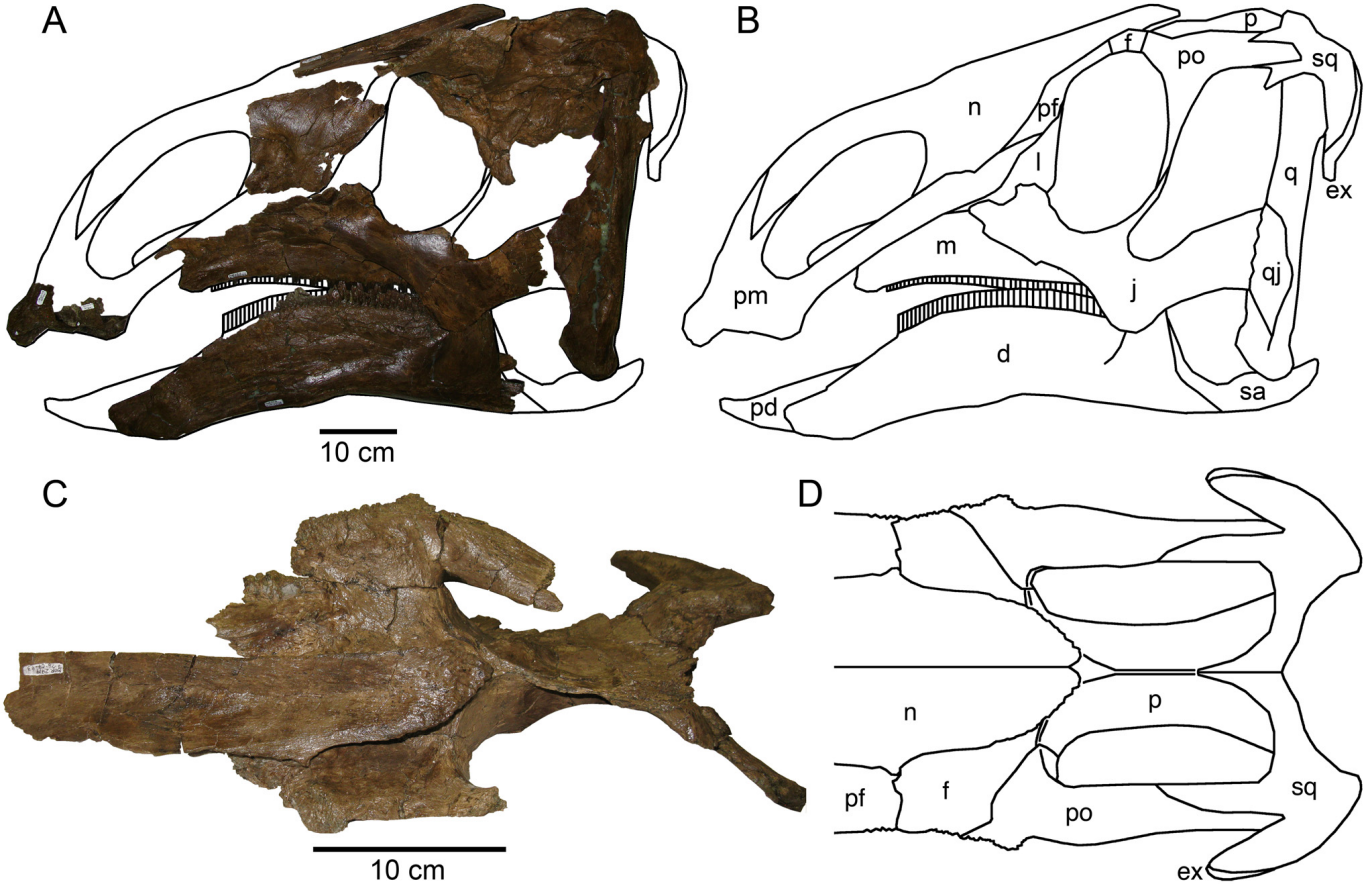
*Probrachylophosaurus bergei* gen. et sp. nov. skull reconstruction. (A) Preserved skull elements of MOR 2919, left lateral view. Predentary not included due to its poor preservation and diagenetic compression. (B) Outline of skull reconstruction, left lateral view. The outline accounts for diagenetic distortion of the posterior braincase, but otherwise does not correct for distortion of skull elements. Outlined regions where left skull material was not preserved are based on right bones when available. Regions with neither left nor right material preserved are hypothesized reconstructions based on *Brachylophosaurus canadensis* skulls. (C) Braincase with left nasal crest, dorsal view. (D) Outline of braincase reconstruction with nasal crest, dorsal view. Reconstruction accounts for diagenetic lateral compression and distortion of posterior braincase. *Abbreviations*: *d*, dentary; *ex*, exoccipital; *f*, frontal; *j*, jugal; *l*, lacrimal; *m*, maxilla; *n*, nasal; *p*, parietal; *pd*, predentary; *pf*, prefrontal; *pm*, premaxilla; *po*, postorbital; *q*, quadrate; *qj*, quadratojugal; *sa*, surangular; *sq*, squamosal.

MOR 2919, majority of a skull and skeleton, disarticulated. Cranial material includes a right premaxilla (fragmentary), both maxillae, left jugal, partial right lacrimal, left posterior nasal, partial mid-region of right nasal, articulated braincase (with articulated frontals, parietal, postorbitals, and exoccipitals), both squamosals, both quadrates, predentary, both dentaries, and right surangular (Fig 3). Postcranial material includes atlas fragments and at least 10 other cervical vertebrae, 11 dorsal vertebrae, 29 caudal vertebrae, 19 chevrons, approximately 19 ribs, both ilia, both pubes, both ischia, both tibiae, both fibulae, both astragali, right metatarsal II, and right metatarsal IV. Forelimbs and sacral vertebrae are absent, although an isolated neural spine may belong to a sacral vertebra.

#### Referred specimen

MOR 1097, fragmentary subadult skull material including the right posterior nasal crest, right jugal, left coronoid process of dentary, dentary tooth rows, maxilla tooth rows, partial left prefrontal, and dorsal and ventral condyles of right quadrate.

#### Etymology

Species name *bergei* in memory of Sam Berge, co-owner of the land where the specimen was discovered, and friend and relative of many members of the Rudyard, Montana community, who have supported paleontologic research for decades. Pronunciation: berg-ee-i

#### Horizon and locality

**Fig 4:**
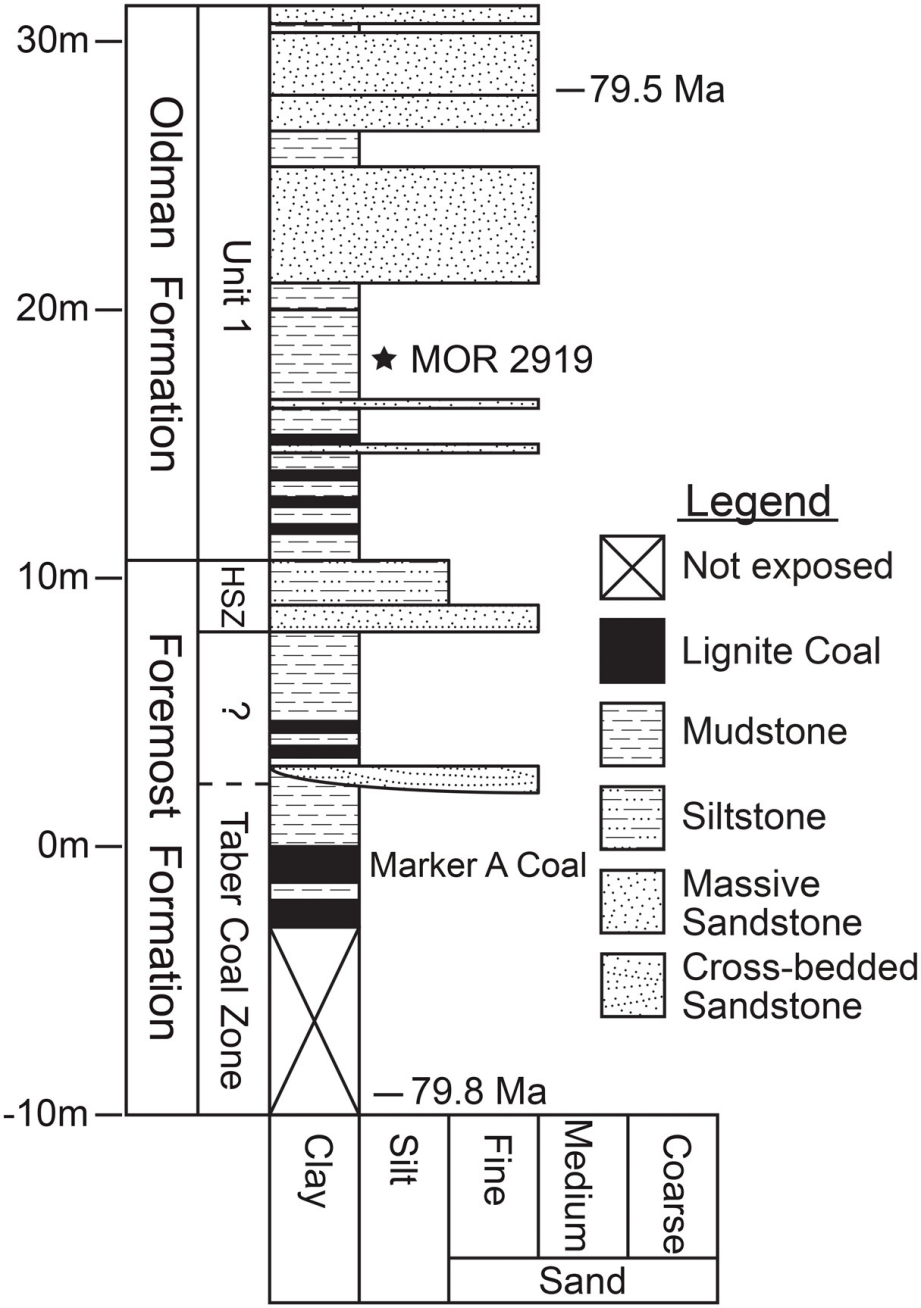
Stratigraphic section at *Probrachylophosaurus bergei* gen. et sp. nov. MOR 2919 quarry, MOR locality JR-518. Datum (0 m) is the top of the Marker A coal of the Taber Coal Zone of the Foremost Formation. Radiometric dates were recalibrated from Goodwin and Deino [11] samples collected southwest of MOR JR-518 in Kennedy Coulee; their stratigraphic heights are indicated on the section. This section is atypical for Kennedy Coulee sections in that it has a relatively thin Herronton Sandstone Zone (HSZ) low in section, and more sandstones in the upper part of the section, whereas most of the coulee has a thick sandstone zone above the Marker A coal, and is dominated by mudstone in the upper regions. The region indicated with “?” below the HSZ may or may not be classified as a continuation of the Taber Coal Zone.

MOR 2919 was collected from private land north of Rudyard, Montana, just east of the mouth of Kennedy Coulee along the Milk River near the USA-Canada border, in exposures of the Judith River Formation. The site, MOR locality JR-518 (“Superduck”), is within a grey mudstone stratigraphically equivalent to Unit 1 of the Oldman Formation of Alberta. The bone horizon is 17.5 m above the top of the Marker A Coal of the Taber Coal Zone of the Foremost Formation, and 7.0 m above the top of the Herronton Sandstone Zone of the Foremost Formation (Fig 4), with a recalibrated age between 79.5 +/- 0.2 Ma and 79.8 +/- 0.2 Ma [10, 11]. The skeleton was completely disarticulated and there was no preferred orientation of the long bones. Associated microsite material was rare, but dominated by tyrannosaur teeth. MOR 1097 was collected from state-owned land less than 1 km east of MOR 2919, and at a similar stratigraphic height.

The site was discovered by Kyoko Kishi and partially excavated by a UCMP crew led by Mark Goodwin in 1981 and 1994, which collected some postcranial material (UCMP locality V81232). Additional material became exposed in 2007, so a MOR crew continued excavation in 2007 and 2008, collecting the skull and extensive postcrania. Because all skeletal material at the site belongs to a single individual, UCMP graciously transferred its postcranial material to MOR. Consequently, the specimen numbers have been altered; all bones are now MOR 2919, and the old UCMP numbers are now treated as “field numbers” for purposes of identifying individual bones within MOR 2919. Original UCMP collections numbers were: left tibia UCMP 137272, right fibula UCMP 156955, right metatarsal IV UCMP 399999, right ilium UCMP 172484, left astragalus UCMP 172484, and caudal vertebra UCMP 400000. A jacket containing an unprepared and thus previously uncataloged right tibia was also transferred to MOR.

#### Diagnosis

*Probrachylophosaurus bergei* is a hadrosaurine hadrosaurid diagnosed by the following features: solid crest consisting entirely of the nasals that overhangs the supratemporal fenestrae by less than 2 cm in adults; nasal crest being extremely dorsoventrally thickened medially, resulting in a strongly triangular frontal plane cross section, with the dorsal angle formed by the paired nasals in posterior view being less than 130 degrees. These autapomorphies, together with the following traits, form a unique combination of characters: posterior lacrimal mediolaterally wide as in *Acristavus* but not *Brachylophosaurus*, caudoventral apex of the rostral process of the jugal is posterior to the caudodorsal apex as in *Acristavus* but not *Brachylophosaurus*, squamosals contact each other medially as in *Acristavus* but not *Brachylophosaurus*, posteriorly-oriented solid nasal crest as in *Brachylophosaurus* but not *Acristavus*.

## Osteological Description

The focus of this description is to highlight features of MOR 2919 that differ from other specimens of Brachylophosaurini, are phylogenetically significant, or that may be ontogenetically variable in hadrosaurids. Characters not mentioned are either unobservable in the preserved elements of MOR 2919, or are consistent with *Brachylophosaurus canadensis* and related hadrosaurs [1, 23, 33], such that descriptions need not be duplicated here. The comparative specimens of *B*. *canadensis* used herein are identified by specimen number rather than simply genus and species name due to slight morphologic differences among specimens (Table 2). Generally, morphologic descriptions of *B*. *canadensis* are based on the holotype, CMN 8893, for characters observable on an articulated skull, and MOR 1071 for characters only observable on disarticulated specimens. Unless otherwise indicated, all morphologic descriptions of *Acristavus* are based on MOR 1155 (holotype; [3]), *Maiasaura* on YPM-PU 22405 (holotype; [5]), *Gryposaurus* on CMN 2278 (holotype of *Gryposaurus notabilis*; [34]), and *Prosaurolophus* on MOR 454 (holotype of *Prosaurolophus blackfeetensis*; [35]). For features not preserved on the *Maiasaura* holotype, a referred specimen (OTM F138) of uncertain stratigraphic position is used for comparison [36].

MOR 2919 (Fig 3) represents one of the largest brachylophosaurin specimens. Exact comparisons of cranial dimensions among specimens are difficult due to diagenetic compression affecting each specimen to a different degree and direction. However, for specimens with associated postcrania, comparisons are more straightforward (Table 3). Although the relative size of individuals does not necessarily indicate the same degree of relative maturity, the larger size of MOR 2919 suggests that it is an adult, not merely an immature specimen of *B*. *canadensis*; this conclusion is supported by its axial fusion and osteohistology (see Discussion).

**Table 3:** Size comparisons of MOR 2919 and other selected Brachylophosaurini specimens.

Taxon	Specimen	Quadrate		Dentary		Tibia		Fibula		Metatarsal II	
		Height (cm)	% MOR 794	Length (cm)	% MOR 794	Length (cm)	% MOR 794	Length (cm)	% MOR 794	Length (cm)	% MOR 794
*Probrachylophosaurus*	MOR 2919	37.0	114% (adult)	52.5	102% (adult)	120.0	105% (adult)	112.0	109% (adult)	38.0	115% (adult)
	MOR 1097	28.0 (est)	86% (*76% sub)	-	-	-	-	-	-	-	-
*Brachylophosaurus*	MOR 794	32.5	100% (adult)	51.5	100% (adult)	114.0	100% (adult)	103.0	100% (adult)	33.0	100% (adult)
	FMNH 862	32.5 (est)	92% (adult)	45.0	87% (adult)	-	-	-	-	-	-
	MOR 1071	32.5	100% (adult)	45.5	88% (adult)	103.5	91% (adult)	94.0	91% (adult)	36.5	111% (adult)
	MOR 1071	21.3	66% (sub)	29.5	57% (sub)	65.0	57% (sub)	64.5	63% (sub)	23.5	71% (sub)

The value “% MOR 794” indicates the specimen’s size relative to that of MOR 794, the largest *Brachylophosaurus canadensis*, and its categorization as adult or subadult based solely on its size, not its histological maturity; those specimens 50–85% the size of MOR 794 are considered subadults [26]. The size of MOR 1097 is also given relative to MOR 2919 (*76%), which categorizes MOR 1097 as a subadult. The height of incomplete quadrates was estimated; the quadrate of FMNH is nearly complete, and is a similar size to that of MOR 794. The MOR 1097 quadrate estimate was based on the width of the ventral condyle, which scales linearly with height (R^2^ = 0.999) in these specimens. Because MOR 1071 specimens are from a disarticulated bonebed, most bones cannot be associated together as specific individuals. For each element, the largest specimen and a representative subadult specimen were included here. Field numbers of MOR 1071 specimens, largest followed by smaller for each element: quadrates, 8-13-98-559D, 6-30-98-1; dentaries, 8-98-X (part of skull 7-7-98-86), 8-1-99-313; tibiae, 672-L, 8-10-98-514; fibulae, 7-7-98-88, 7-23-99-181; metatarsal IIs, 7-19-99-131B, 8-7-99-470. *Abbreviations*: *est*, estimated; *sub*, subadult.

### Skull—Maxillary/facial complex

#### Premaxilla

The premaxilla is severely crushed and fragmented, and so is of limited morphological use. The anterior portion is best preserved; the posterodorsal and posteroventral processes are absent. The oral margin is anteroposteriorly wide and ventrally deflected as in *Brachylophosaurus* (CMN 8893, MOR 1071) rather than dorsally reflected as in other hadrosaurines (e.g. *Gryposaurus*, *Prosaurolophus* MOR 447). The ventral margin is eroded; no denticles are preserved. Premaxillary foramina are unobservable, either due to crushing or true absence.

#### Maxilla

**Fig 5:**
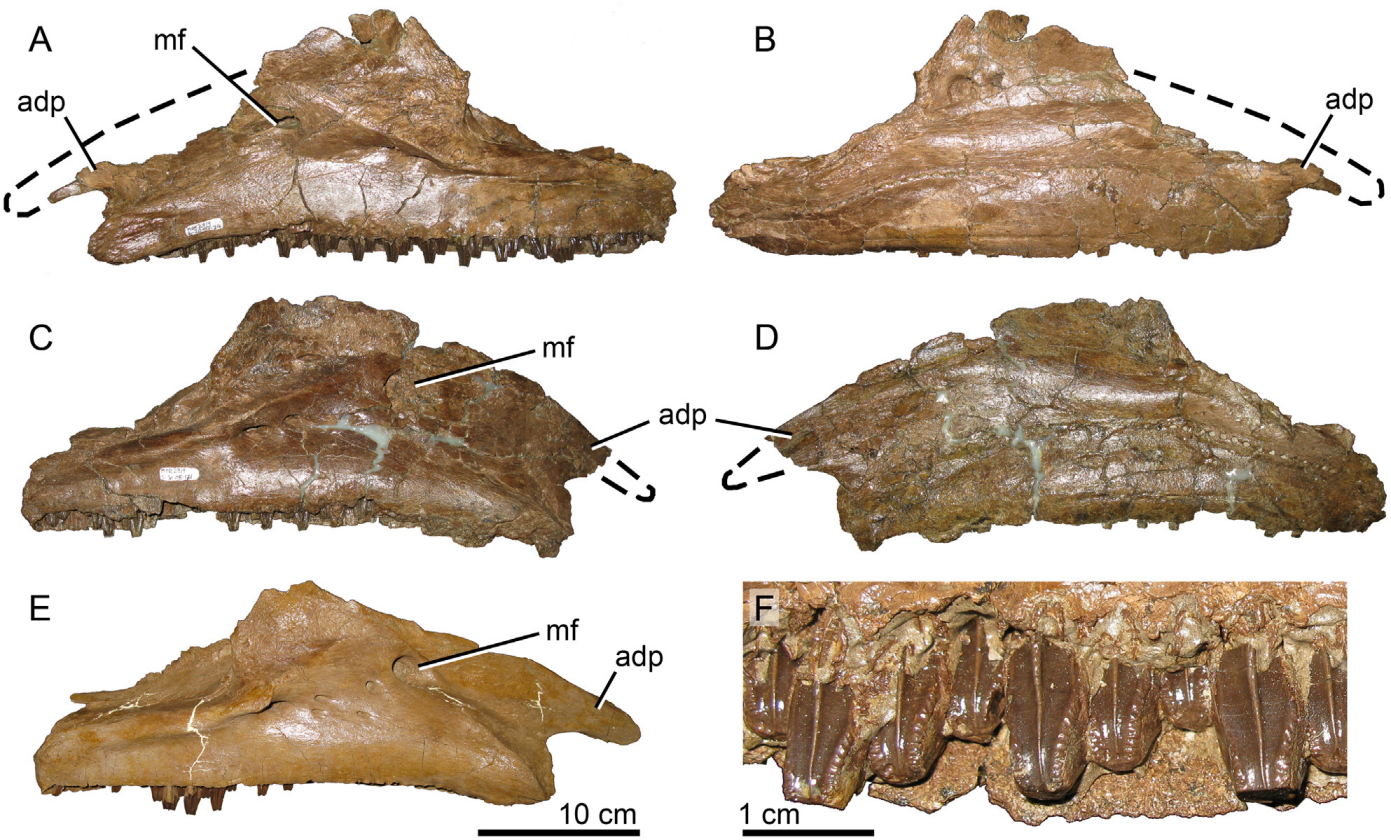
Brachylophosaurin maxillae. (A-D, F) *Probrachylophosaurus bergei* gen. et sp. nov., MOR 2919. Estimated reconstruction of anterodorsal process indicated with dashed line. (A) left maxilla, lateral view; (B) left maxilla, medial view; (C) right maxilla, lateral view; (D) right maxilla, medial view; (E) *Brachylophosaurus canadensis* right maxilla, lateral view MOR 1071-8-13-98-559; (F) teeth of MOR 2919 left maxilla demonstrating slight sinuosity of median carinae. *Abbreviations*: *adp*, anterodorsal process; *mf*, maxillary foramen.

The maxillae of MOR 2919 are consistent in most respects with the morphology of *Brachylophosaurus* (Fig 5). The relative size and placement of the large maxillary foramen in MOR 2919 is consistent with all members of Brachylophosaurini (*Brachylophosaurus* MOR 1071, *Maiasaura* OTM F138, *Acristavus*), although the number and pattern of additional smaller maxillary foramina on the lateral surface can be variable between individuals of the same species. Maxillae from the MOR 1071 *Brachylophosaurus* bonebed have four to five smaller foramina in differing patterns of arrangement. MOR 2919 has three of these smaller foramina on each maxilla. As in *Acristavus*, the posterior maxillary process is broken at its base on both maxillae, but if complete would likely have been similar to that of *Brachylophosaurus*. The anterodorsal maxillary process is also partially broken on both maxillae (anteriorly on the right and anterodorsally on the left), but if complete would resemble that of *Brachylophosaurus*. The ventral margin of the maxilla is nearly straight, with the anterior one-third only slightly ventrally deflected, similar to the condition seen in *Acristavus* and *Brachylophosaurus*.

The left maxilla possesses 52 tooth rows; the right maxilla tooth rows are obscured anteriorly. The occlusal plane of the left maxilla (30.5 cm) of MOR 2919 is slightly longer than that of *Brachylophosaurus* holotype CMN 8893 (right 29 cm, left 28.5 cm), which has 46 tooth rows [23]. In both specimens, tooth sizes are largest in the center of the maxilla, and decrease in size anteriorly and posteriorly [23]. MOR 2919 is able to accommodate its increased density of tooth rows by decreasing its anterior and posterior tooth sizes relatively more than in CMN 8893. Because the number of tooth rows in hadrosaurines increases ontogenetically, the high tooth count in MOR 2919 is likely due to its size and maturity rather than being taxonomically informative. Less than one-third of the teeth that had begun to wear on the occlusal plane remain, so the number of teeth per family exposed on the occlusal plane cannot be determined; most teeth preserved in the maxillae are unworn. Each tooth possesses a single median carina, many of which are subtly sinusoidal (Fig 5F), as in *Brachylophosaurus* (FMNH PR 862). The edges of the crowns have papillae that are slightly smaller but more prominent than those of *Brachylophosaurus* (MOR 1071).

#### Jugal

**Fig 6:**
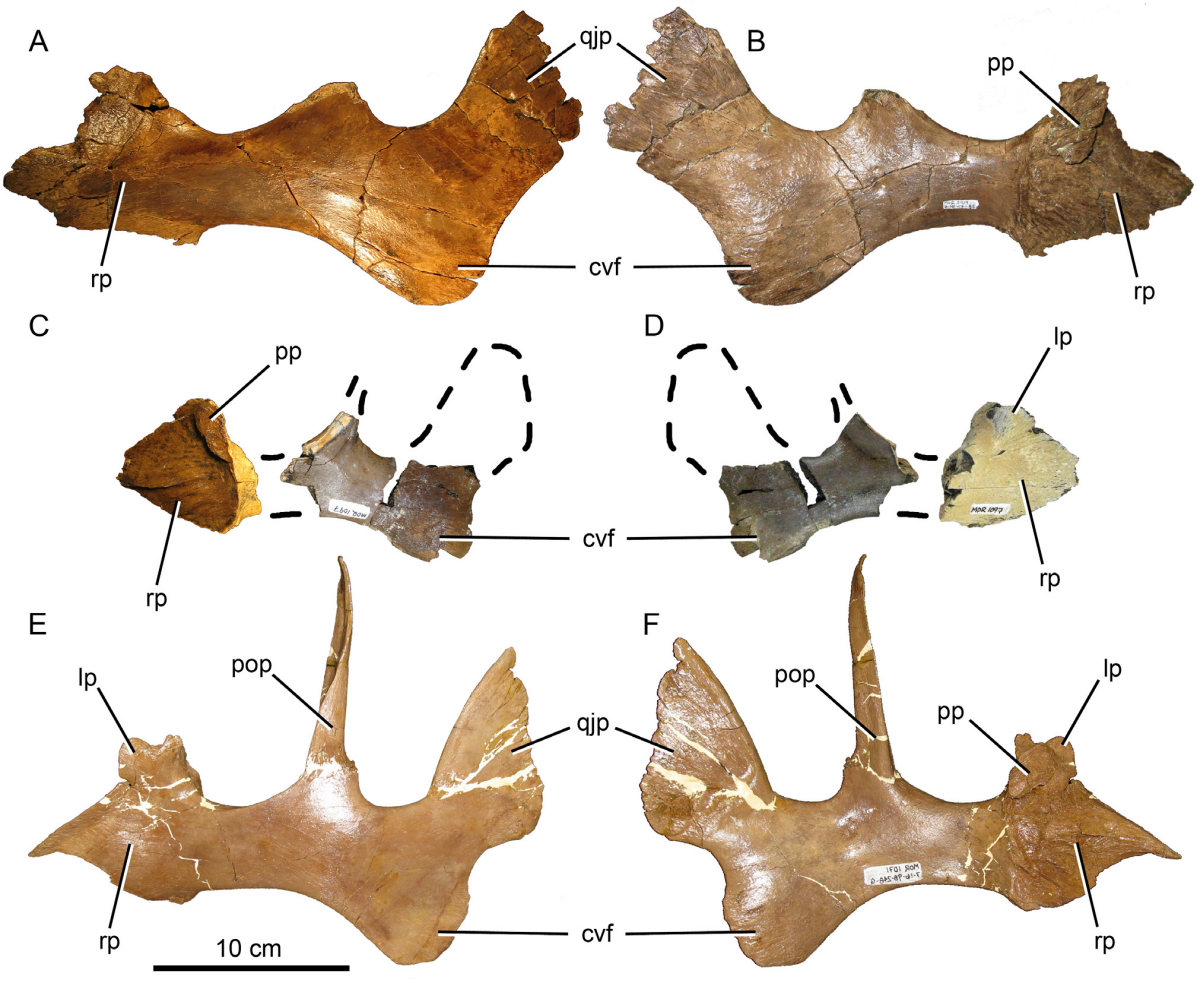
Brachylophosaurin jugals. (A, B), *Probrachylophosaurus bergei* gen. et sp. nov. adult, MOR 2919, left jugal; (A) lateral view; (B) medial view. (C, D) *P*. *bergei* subadult, MOR 1097, right jugal reconstructed with dashed line; (C) medial view; (D) lateral view. (E, F) *Brachylophosaurus canadensis* adult, MOR 1071-7-16-98-248-Q, right jugal reversed. Note that the postorbital process was broken at its base and should be inclined more posteriorly; (E) lateral view; (F) medial view. *Abbreviations*: *cvf*, caudoventral flange; *lp*, lacrimal process; *pop*, postorbital process; *pp*, palatine process; *qjp*, quadratojugal process; *rp*, rostral process.

On the medial surface of the rostral (maxillary) process of subadult and adult *Probrachylophosaurus* (Fig 6), the palatine joint is obliquely oriented, raised medially, and extends anteriorly to form a lip as in *Brachylophosaurus* and *Acristavus*. The adult MOR 2919 palatine process extends the farthest anteriorly, forming a narrow slot below its 10 mm overhang (Fig 6B), and most closely matches that of adult *Brachylophosaurus* MOR 1071-7-16-98-248-Q (Fig 6F, lip 5 mm). The anterior curvature that forms the lip in the subadult MOR 1097 is gentle and forms a small overhanging lip (Fig 6C, 3 mm) identical in size and morphology to *Acristavus*. The lip in *Maiasaura* is variably developed, overhanging 4 mm in the holotype YPM-PU 22405 but only 1 mm in OTM F138. The palatine joint does not form an overhanging lip in *Prosaurolophus* or *Gryposaurus* (MOR 478, *G*. *latidens*).

In *Brachylophosaurus* (CMN 8893, FMNH PR 862, MOR 794, MOR 1071), the caudodorsal and caudoventral apices of the rostral process are in line vertically when the rostral process is oriented horizontally. In MOR 1097, MOR 2919, and *Acristavus* (contra [29]), the caudoventral apex is posterior to the caudodorsal apex when the rostral process is oriented horizontally (character 106/J4 in [29]). In *Maiasaura* (YPM-PU 22405, OTM F138), the caudoventral apex is only slightly posterior to the caudodorsal apex. The lacrimal process on the dorsal rostral process is broken in MOR 2919, but intact in MOR 1097, where it resembles that of *Brachylophosaurus*, although it is relatively slightly larger in MOR 1097. The ventral margin of the rostral process is straight in MOR 1097 and MOR 2919, as in *Acristavus*. The ventral margin is sigmoidal in lateral view in *Brachylophosaurus* (CMN 8893, MOR 1071) and somewhat sigmoidal in one *Maiasaura* specimen (OTM F138), although it is straight in the *Maiasaura* holotype (YPM-PU 22405).

The ascending postorbital process is broken at its base, but bone texture at the base indicates that the postorbital process would have been angled to the same degree as in *Brachylophosaurus*. The ventral margin of the orbit is wider than the ventral margin of the lateral temporal fenestra, as in *Brachylophosaurus*, *Acristavus*, and *Maiasaura*.

MOR 2919 has a relatively wider (dorsoventrally) caudal constriction of the jugal than do *Brachylophosaurus* (CMN 8893, MOR 1071-7-16-98-248-Q), *Acristavus*, and *Maiasaura*, although MOR 1097’s caudal constriction is narrower than in these other taxa. The ratio of the depth of the caudal constriction to the anterior constriction in MOR 2919 is 1.42 (character 113/J11 in [29]). The ratio of dorsoventral depth of the caudoventral flange to the caudal constriction is 1.45 (character 110/J8 in [29]). These ratios are 1.40 and 1.61, respectively, in the *Brachylophosaurus* holotype (CMN 8893). The caudoventral flange of MOR 2919 is dorsoventrally deep, as in other brachylophosaurins, but relatively wider anteroposteriorly than in *Brachylophosaurus*.

The concavity of the posterior jugal margin between the quadratojugal process and caudoventral flange is weakly developed in MOR 2919, as in *Acristavus* and *Maiasaura*. The concavity is deeper in *Brachylophosaurus*. The medial side of the quadratojugal process is more gently excavated in MOR 2919 than in *Brachylophosaurus* or *Maiasaura*. The excavation in *Acristavus* is slightly more pronounced than in MOR 2919, but less than in *Brachylophosaurus*.

#### Lacrimal

**Fig 7:**
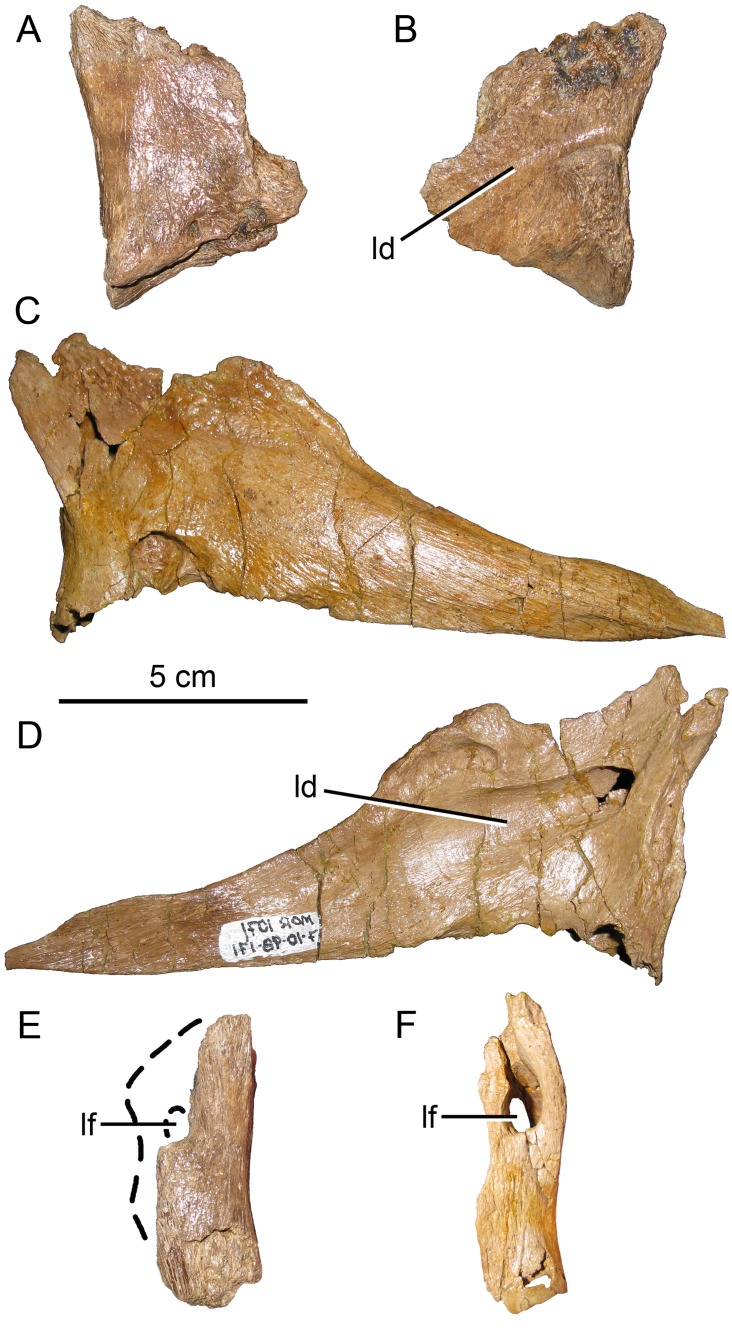
Brachylophosaurin lacrimals. (A, B, E) *Probrachylophosaurus bergei* gen. et sp. nov., MOR 2919, posterior right lacrimal; (A) lateral view; (B) medial view; (C, D, F) *Brachylophosaurus canadensis*, MOR 1071-7-10-98-171, left lacrimal reversed; (C) lateral view; (D) medial view; (E) posterior view MOR 2919, medial portion reconstructed with dashed line; (F) posterior view MOR 1071-7-10-98-171. *Abbreviations*: *ld*, lacrimal duct; *lf*, lacrimal foramen.

The posterior right lacrimal preserves part of the anterior orbital margin and anteroventrally oriented lacrimal duct (Fig 7). The medial portion of the lacrimal is not preserved, so only the lateral side of the lacrimal foramen is present; the foramen’s original size and shape cannot be determined. However, the bone lateral to the foramen is robust (Fig 7E), over twice as mediolaterally thick as the corresponding region in *Brachylophosaurus* MOR 1071-7-10-98-171 (Fig 7F), consistent with the robusticity described for *Acristavus* [3]. The posterior jugal contact is robust and ventrally convex, whereas it is strongly ventrally concave in *Brachylophosaurus*.

#### Nasal

**Fig 8:**
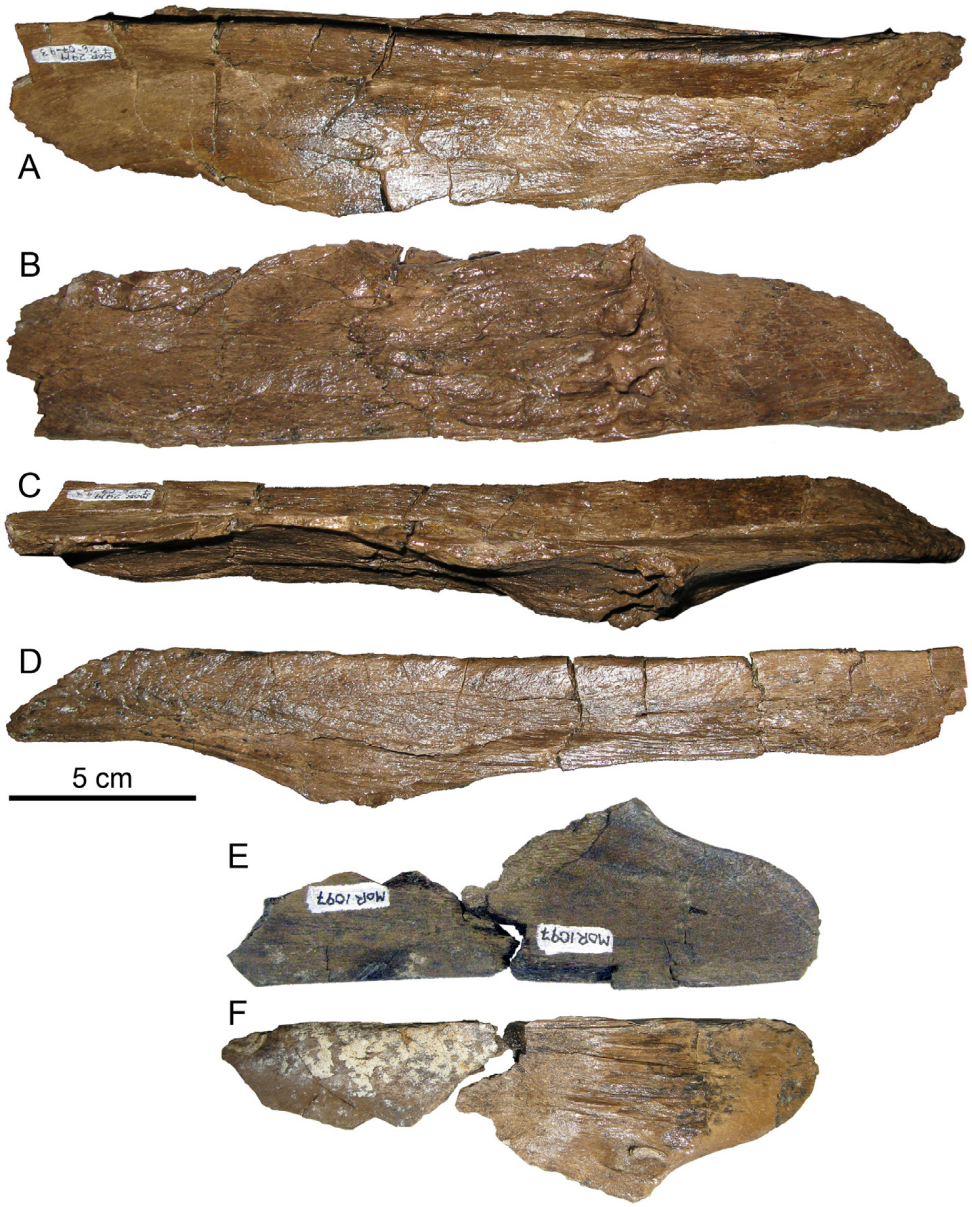
*Probrachylophosaurus bergei* gen. et sp. nov. nasals. (A-D) Adult, MOR 2919, posterior left nasal, crest pointing right except for (D); (A) dorsal view; (B) ventral view showing rugose nasofrontal suture; (C) lateral view; (D) medial view. (E, F) Subadult, MOR 1097, posterior right nasal; (E) dorsal view; (F) ventral view showing linearly striated nasofrontal suture.

**Fig 9:**
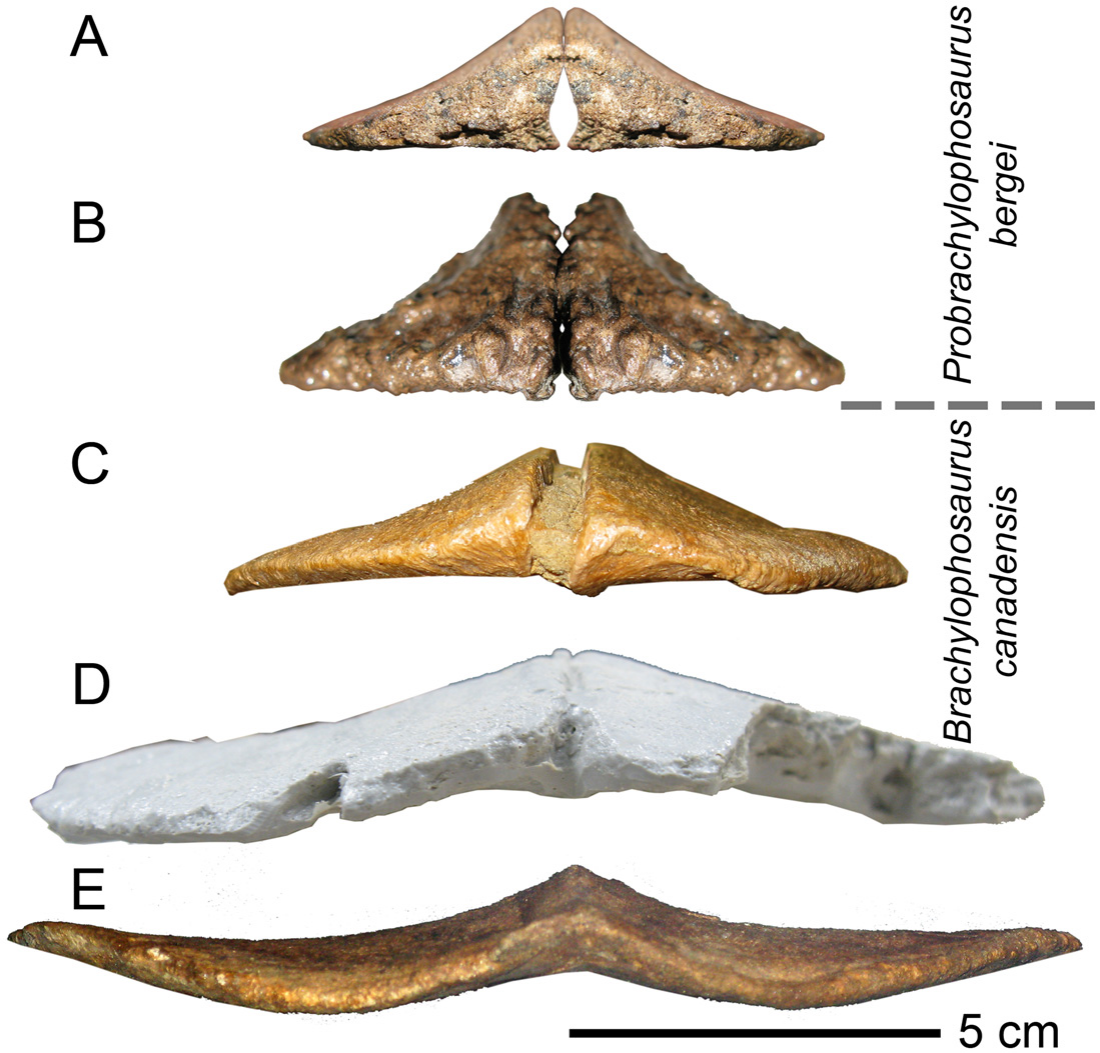
Brachylophosaurin nasals, posterior view. In *Probrachylophosaurus bergei* gen. et sp. nov. and slender *Brachylophosaurus canadensis* morphotypes, the nasal crest is narrow and triangular in cross section. In *B*. *canadensis*, the nasal crest flattens as it grows wider. (A) *Probrachylophosaurus bergei* subadult, MOR 1097; (B) *P*. *bergei* adult, MOR 2919; (C) *Brachylophosaurus canadensis* slender morphotype, MOR 1071-7-7-98-86; (D) *B*. *canadensis* intermediate morphotype, FMNH PR 862; (E) *B*. *canadensis* robust morphotype, MOR 794.

The size and shape of the nasal crest is the main distinguishing character between *Probrachylophosaurus* and *Brachylophosaurus*. The nasal crest of *Probrachylophosaurus* (MOR 1097, MOR 2919; Figs 8 and 9) is shorter, and more triangular in cross-section, than equivalently aged *Brachylophosaurus* specimens. The crest consists entirely of the nasals, and overhangs the supratemporal fenestrae by less than 1 cm in the adult (Fig 3). The length of the nasal crest that extends posterodorsally, overhanging the posterior frontals, is 8.0 cm in the adult (MOR 2919) and 3.2 cm in the subadult (MOR 1097). The nasal crest is extremely dorsoventrally thickened medially, and rapidly thins laterally. The dorsal angle formed by the paired nasals, in posterior view, is 112° in the adult (MOR 2919; Fig 9B) and 126° in the subadult (MOR 1097; Fig 9A). The crest tapers posteriorly in a “V” shape. The posteriormost dorsal surface of the nasal crest is rugose in MOR 2919 but smooth in MOR 1097 (Fig 8).

**Fig 10:**
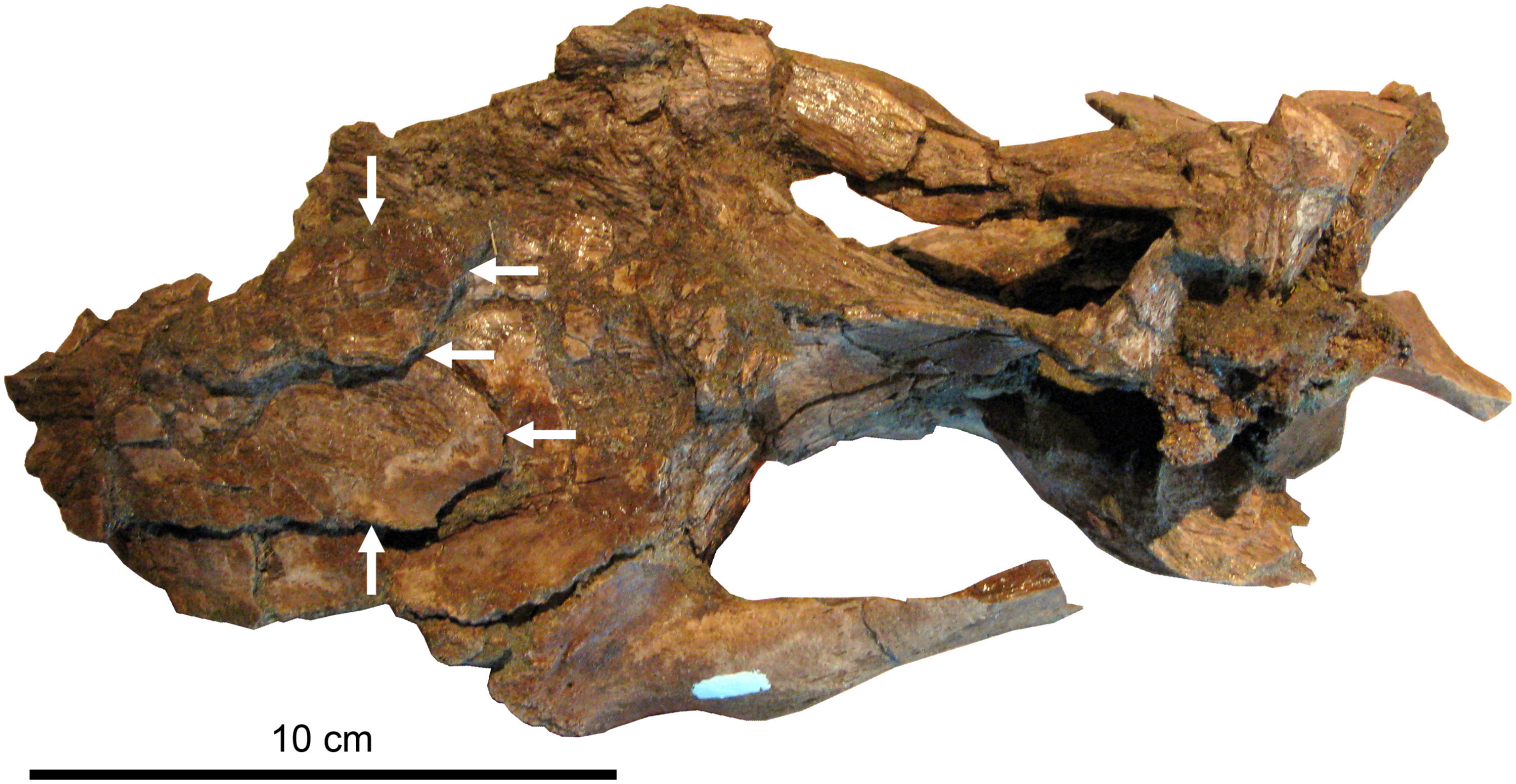
Subadult *Brachylophosaurus canadensis* braincase MOR 940 in dorsal view. Anterior is to the left. Specimen is from the Judith River Formation near Malta, Montana, in a horizon equivalent to the Comrey Sandstone Zone of the middle Oldman Formation. Borders of the nasals are indicated by white arrows. The specimen is heavily fractured, and the nasals are slightly elevated above the frontals, but this is due to fracturing; when undeformed, the nasals would have lain flush over the frontals, with no open space in between. There is no projection forming an overhanging, posteriorly oriented nasal crest as would be seen in larger *Brachylophosaurus canadensis* specimens.

In adult *Brachylophosaurus*, the exposed nasal crest is composed entirely of the nasals, but the frontal and/or prefrontal platform does extend posteriorly over the supratemporal fenestrae to support the base of the nasal crest (see frontal description below). The flat, paddle-like nasal crest of *Brachylophosaurus* varies in size amongst individuals. Prieto-Márquez (2005) categorized specimens as “slender” or “robust”. The nasal crest of slender specimens (MOR 1071-7-7-98-86, MOR 1071-7-16-98-248) covers most of the length of the supratemporal fenestrae, but does not reach their posterior or lateral margins. The crest tapers posteriorly in a “U” shape, resulting in the crest overhanging approximately 50% of the area of the supratemporal fenestrae. The crest is thickened medially; the dorsal angle of the paired nasals is 140° in MOR 1071-7-7-98-86 (Fig 9C). The nasal crest of the robust MOR 794 is much longer and wider, overhanging the entire supratemporal fenestrae and extending posteriorly past the supraoccipital; the dorsal angle of the paired nasals is 165° (Fig 9E). These slender and robust categories are not discrete; some *Brachylophosaurus* specimens (CMN 8893, FMNH PR 862, TMP 1990.104.001) possess intermediate morphologies, completely overhanging the supratemporal fenestrae posteriorly but not laterally. The crests of FMNH PR 862 and TMP 1990.104.001 are nearly robust, while CMN 8893 more closely resembles an enlarged slender morphology. These intermediate crest morphologies also have intermediate dorsal angles of the paired nasals: 151° in FMNH PR 862 (Fig 9D) and 157° in CMN 8893. The nasals of subadult *Brachylophosaurus canadensis* (MOR 940) appear to remain flat against the frontal and do not form a crest (Fig 10).

The nasofrontal articulation surface of MOR 2919 is rugose, and the posterior articulation is composed of deep pits (up to 12 mm) and projections to interlock with those of the frontal (Fig 8B). The nasofrontal articulation surface of MOR 1097 consists of anteroposterior striations and a single pit on the posterolateral margin (Fig 8F). The striations on the subadult MOR 1097 nasofrontal articulation surface correlate well to the striations on the frontals of subadult *Brachylophosaurus* (see frontal discussion below). No disarticulated nasals of *Brachylophosaurus* were available for comparison.

The anterior nasals are not preserved, and no part of the nasals or premaxilla preserves more than a few centimeters of possible narial margin, if any. However, the lateral middle region of the left nasal is preserved, with articulation surfaces for the prefrontal, lacrimal, and posteroventral process of the premaxilla. On the lateral nasal surface, dorsal to the lacrimal articulation, is a subcircular depression 2 cm in diameter that includes a small fenestra. On the medial side in the same location is an anteroventrally-posterodorsally oriented excavation 4 cm long, 1 cm wide, and 1 cm deep; the small fenestra opens halfway along the length of the excavation. This excavation may correspond to the “anteroposteriorly oriented groove terminating in elongated foramen” described for *Brachylophosaurus* [1]. However, the excavation in MOR 2919 is located on the medial side of the lateral nasal anterior to the prefrontal, whereas in MOR 1071-7-7-98-86 it is on the ventral side of the dorsal nasal medial to the prefrontal. Alternatively, the excavation in MOR 2919 may be pathologic, and possibly associated with the pathologies on both dentaries.

#### Prefrontal

No prefrontals are preserved in MOR 2919. MOR 1097 includes a partial left prefrontal, missing the posterior orbital region. Its morphology is generally similar to that of *Brachylophosaurus*.

#### Frontal

**Fig 11:**
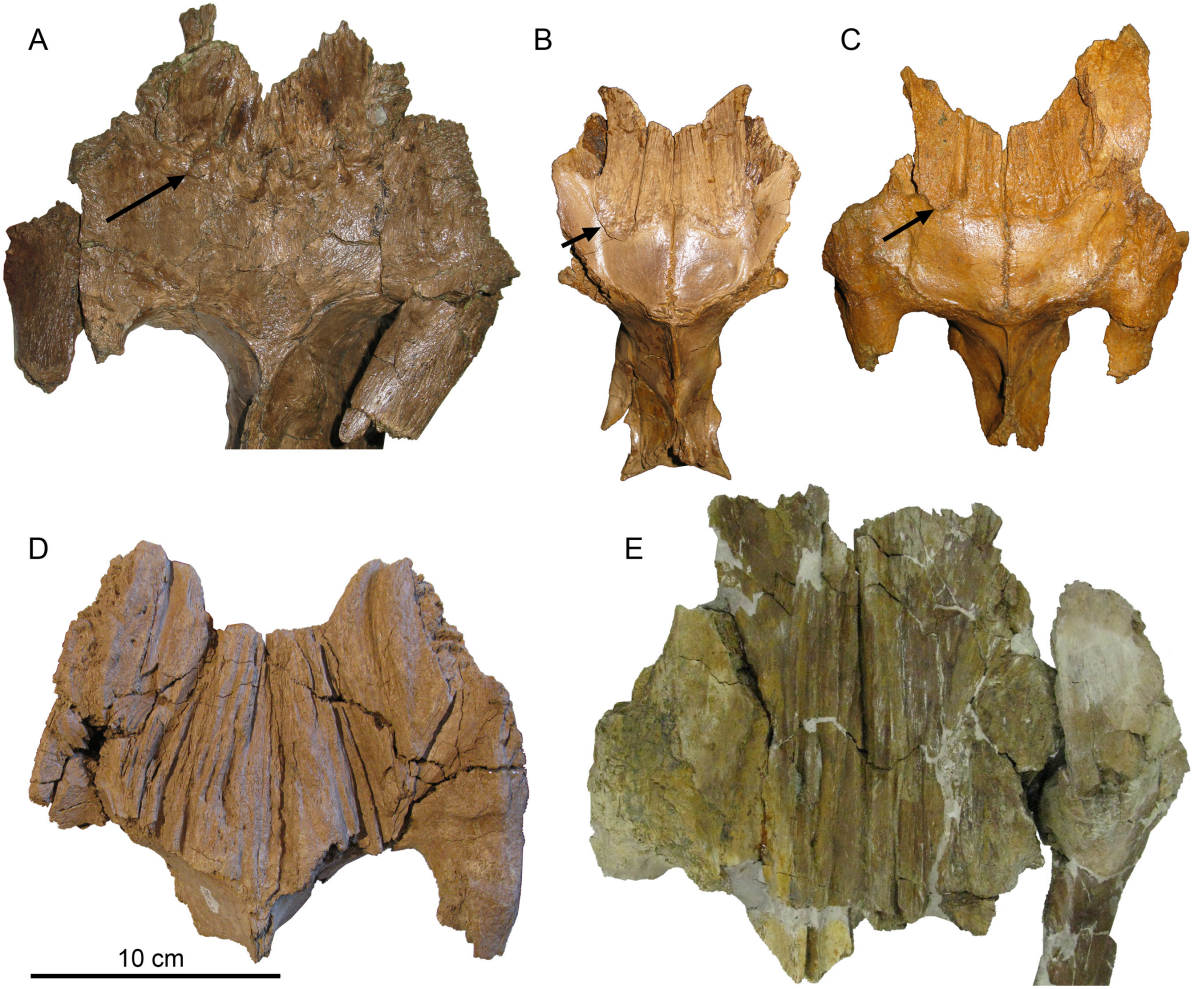
Posterior migration of brachylophosaurin nasofrontal suture with ontogeny and stratigraphic age. In the stratigraphically oldest specimen, the adult *Probrachylophosaurus bergei* gen. et sp. nov., (A) MOR 2919, from the Judith River Formation equivalent of Unit 1 of the lower Oldman Fm, the nasofrontal suture extends over less than half of the frontals. This suture extends over approximately half of the frontals in subadult *Brachylophosaurus canadensis* specimens (B) MOR 1071-7-13-99-87-I and (C) MOR 1071-C-3-3, from the Judith River Formation equivalent of the Comrey Sandstone of the middle Oldman Fm, and migrates posteriorly to completely cover the frontals in the adult (D) GPDM JRF.65, a condition also seen in adult *B*. *canadensis* from the upper Judith River Formation, (E) MOR 720. Border of nasofrontal suture in (A-C) indicated with arrows.

The anterior margin of the frontals in MOR 2919 forms a posteriorly directed “V” (Fig 11), similar to that seen in the “slender” adult *Brachylophosaurus* MOR 1071-7-7-98-86. The anterior frontals in subadult *Brachylophosaurus* MOR 1071 braincases form posteriorly directed “U” shapes. Both are distinct from the rectangular anterior frontal margin of *Acristavus*, with its midline slight anterior projection. The adult *Brachylophosaurus* skull GPDM JRF.65 has the rectangular margin of *Acristavus* but without the midline anterior projection.

The frontals of MOR 2919 at the orbital margin are 2.2 cm thick, rugose, and contribute a length of 2.3 cm to the orbital margin with no indentation between the prefrontal and postorbital, similar to that of *Acristavus* (MOR 1155). The area near the orbital margin of UCMP 130139 is too poorly preserved to observe the locations of sutures, and so the presence, absence, or extent of frontal contribution to the orbital margin cannot be determined; as in MOR 2919, there is no indentation between the prefrontal and postorbital regions. In *Brachylophosaurus*, the frontal margin may be partially or entirely recessed in a medial indentation (0 to 1 cm deep) in the dorsal orbital margin between the prefrontal and postorbital. The base of this indentation exposes a length of 0 to 3 cm of the frontal to the orbital margin (CMN 8893, FMNH PR 862, MOR 794, all MOR 1071 braincases).

#### Nasofrontal suture

**Fig 12:**
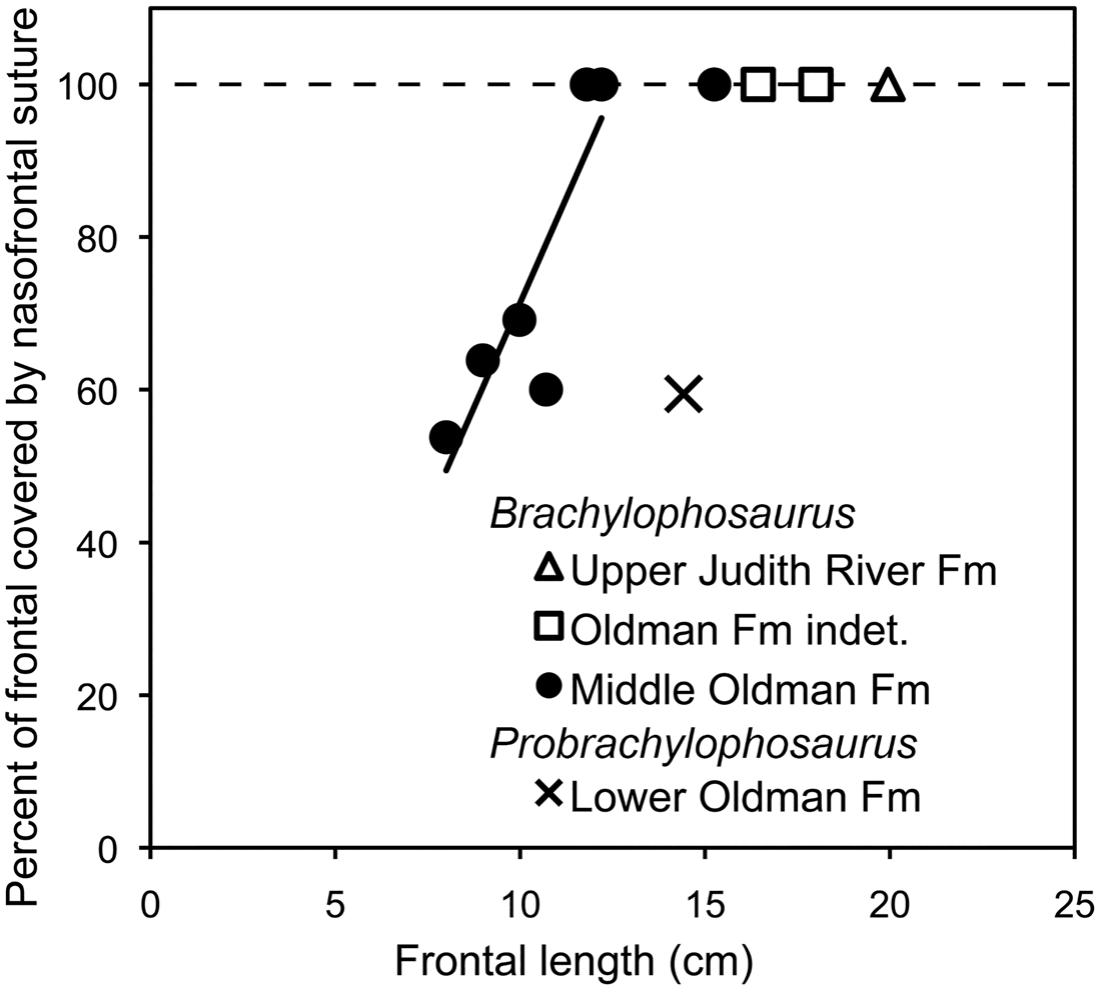
Posterior migration and enlargement of nasofrontal suture with growth in brachylophosaurins. In subadult *Brachylophosaurus*, the nasofrontal suture covers 50–75% of the dorsal frontal surface; in adults, the nasofrontal suture covers the entire dorsal surface. In *Brachylophosaurus* specimens from the Comrey Sandstone Zone of the middle Oldman Formation and its Judith River Formation equivalent in Malta, Montana (solid black circles; CMN 8893, JRF.65, MOR 940, and MOR 1071 specimens C-3-3, 6-30-98-4, 7-13-99-87-I, and 7-7-98-86), the relative coverage of the nasofrontal suture increases with frontal length until the frontal is entirely covered by the nasofrontal suture. Larger adult *Brachylophosaurus* braincases from an unknown stratigraphic height in the Oldman Formation of Alberta (open squares; FMNH PR PR 862, TMP 1990.104.001) and the Upper Judith River Formation of central Montana (open triangle; MOR 720; stratigraphic height relative to Oldman Formation of Alberta unknown) also have the frontals entirely covered by the nasofrontal suture. *Probrachylophosaurus* gen. nov. from the Montana Judith River Formation equivalent of the lower Oldman Formation (black x; MOR 2919) differs from the *Brachylophosaurus* growth trajectory in having a subadult *Brachylophosaurus* degree of coverage in an adult sized skull.

In MOR 2919, the nasofrontal suture is rugose, composed of anteroposterior linear striations, with pits at its posterior margin. The suture covers approximately half (59%) of the dorsal frontal surface, resembling the subadult condition of *Brachylophosaurus* (Figs 11A and 12). Two small subadult specimens of *Brachylophosaurus*, MOR 1071-7-13-99-87-I (Fig 11B) and MOR 1071-C-3-3 (Fig 11C), allot 69% and 60%, respectively, of their frontal surfaces to the nasofrontal suture; the suture surfaces are covered with anteroposterior linear striations but are not pitted posteriorly. A slightly larger subadult, MOR 1071-6-30-98-4, has a suture at least 64% the length of the frontals (frontals are slightly broken anteriorly), and the surface includes both anteroposterior linear striations and minor posterior pitting. Another subadult specimen, MOR 940, still has the nasals articulated onto the frontals; the nasals cover 54% of the dorsal frontal surface, and do not rise above the frontals in a crest (Fig 10). All currently known *Brachylophosaurus* specimens with an overhanging nasal crest have a nasofrontal suture that covers 100% of the frontals (CMN 8893, FMNH PR 862, MOR 794, MOR 1071-8-98-86, MOR 1071-7-16-98-248). A growth stage of *Brachylophosaurus* with an incipient crest between the stages of the non-crested subadult MOR 940 and the large-crested specimens is hypothesized but currently unknown.

The posteriormost margin of the nasofrontal articulation is mediolaterally straight in MOR 1071-C-3-3 and MOR 1071-6-30-98-4, but in MOR 1071-7-13-99-87-I and MOR 940 the medial portion is indented anteriorly (the “M-shape” of Prieto-Márquez, 2005). The margin is gently convex posteriorly in MOR 2919 with no medial indentation. The interfrontal-parietal articulation is posterodorsally elevated in MOR 2919, as noted for *Brachylophosaurus* [1]. In the MOR 1071 subadult braincases, a raised lip defines the posterior margin of the nasofrontal articulation. The posterior margin in MOR 2919 lacks any line or lip, and instead undulates with the rugosities and posterior pits.

In adult *Brachylophosaurus*, the nasofrontal suture covers the entire anteroposterior length of the frontals (Figs 11D–11E and 12). The platform supporting the nasals is posteriorly elongated to overhang the supratemporal fenestrae by a short distance. In CMN 8893, FMNH PR 862, and MOR 1071-7-7-98-86, this projection appears to be composed entirely of the prefrontals, but in MOR 720, it is composed of the frontals. The longer the nasal crest, the more the prefrontals and/or frontals are elongated posteriorly. The gracile-crested MOR 1071-7-7-98-86 possesses prefrontals that overhang the supratemporal fenestra by 2 cm, whereas the more robust FMNH PR 862 possesses prefrontals that overhang by 4 cm. The posteriorly incomplete frontals of MOR 720, a larger individual than FMNH PR 862, would have overhung the fenestrae by at least 5 cm. In subadult specimens where the nasofrontal suture does not cover the entire frontals, the prefrontals and frontals do not protrude over the supratemporal fenestrae at all (MOR 1071-7-13-99-87-I, MOR 1071-C-3-3, MOR 1071-6-30-98-4).

Posterior to the nasofrontal articulation, the frontals of MOR 2919 are shallowly depressed by 5 to 7 mm. *Acristavus* (MOR 1155) also possesses shallow frontal depressions. Frontal depressions are not observed in adult *Brachylophosaurus* due to complete coverage by the nasofrontal articulation. The MOR 1071 subadult *Brachylophosaurus* specimens have small anteroposteriorly elongated frontal depressions 3 to 4 mm deep near the frontal-postorbital sutures, more laterally than those of MOR 2919. The dorsal sutures between the frontals and postorbitals of MOR 2919 are faintly visible, but are fused and nearly completely remodeled.

The frontals of the holotype of *Brachylophosaurus goodwini*, UCMP 130139, are fragmented and poorly preserved, but UCMP 130139 remains unique among Brachylophosaurini specimens in possessing deep frontal depressions (at least 2 cm deep). Horner [9] measured the frontal thickness at the deepest point of the depression in UCMP 130139 as 5 mm thick. The corresponding region in MOR 2919 is 26 mm thick on the right side and 22 mm thick on the left side (slightly crushed).

#### Parietal

The lateral suture between the parietal and laterosphenoid is fused but still faintly visible. The overall morphology of the parietal is consistent with that of *Brachylophosaurus* and *Acristavus* [1, 3, 23].

#### Postorbital

**Fig 13:**
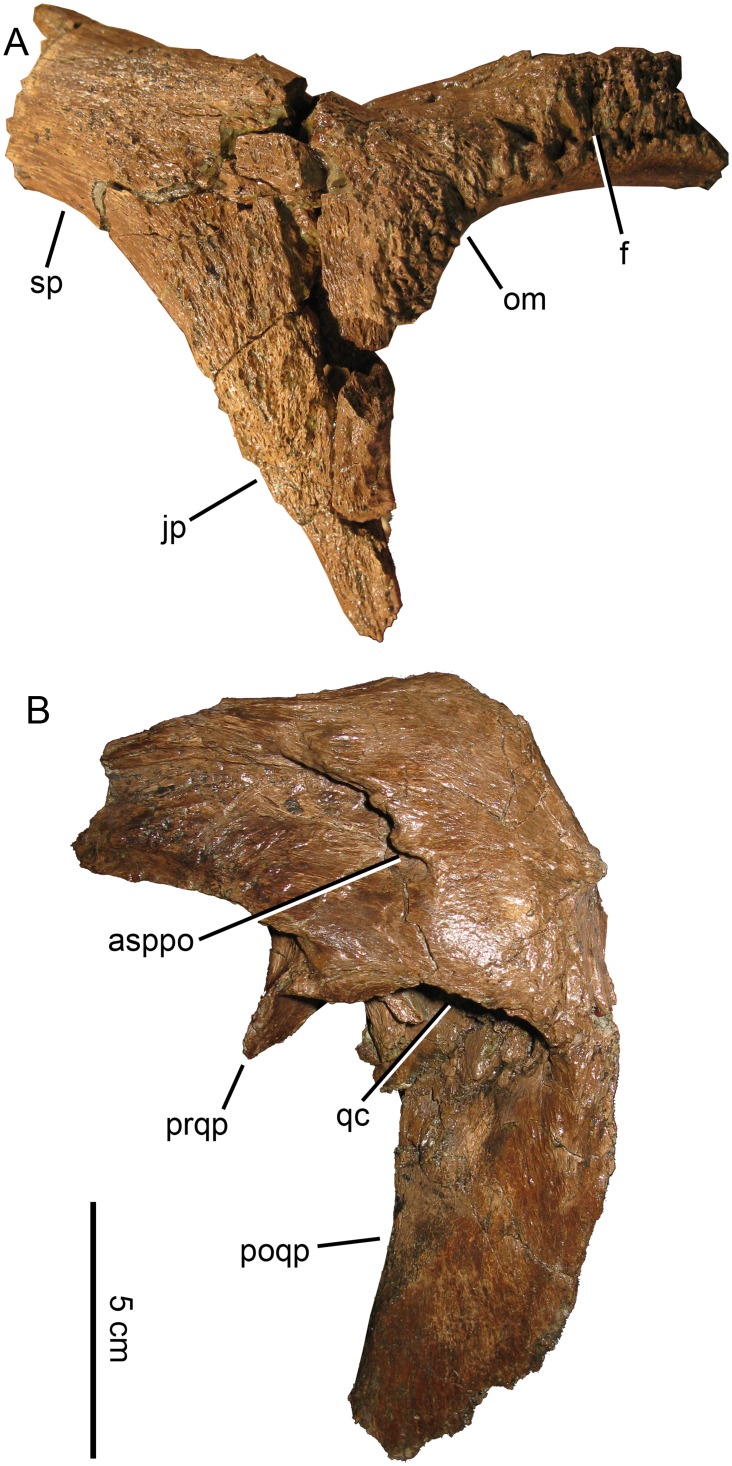
*Probrachylophosaurus bergei* gen. et sp. nov. postorbital and squamosal. MOR 2919 (A) right postorbital and orbital margin of frontal, lateral view; (B) left squamosal, lateral view. *Abbreviations*: *asppo*, articulation for the squamosal process of the postorbital; *f*, frontal; *jp*, jugal process; *om*, orbital margin; *poqp*, postquadratic process; *prqp*, prequadratic process; *qc*, quadrate cotylus; *sp*, squamosal process.

The orbital margin of the postorbital in MOR 2919 is similarly rugose to that of the frontal (Fig 13A). The lateral surface of the jugal process varies amongst specimens of Brachylophosaurini, and is highly intraspecifically variable within *Brachylophosaurus canadensis*. Thus it may not be a character of taxonomic value. The lateral surface is rough and straight in MOR 2919. This surface is smooth and concave in some *Brachylophosaurus* (MOR 794, MOR 1071-7-7-98-86). In other *Brachylophosaurus* (FMNH PR 862), the superior portion of the jugal process is smooth and gently concave, but inferiorly becomes rough and straight, identical to the condition in *Maiasaura* and Brachylophosaurini indet. (UCMP 130139). Cuthbertson and Holmes [23] describe the lateral surface of the *Brachylophosaurus* holotype (CMN 8893) as “deeply pitted”, which is more similar to FMNH PR 862 and MOR 2919 than to the specimens from Malta, Montana (MOR 794, MOR 1071-7-7-98-86). The right postorbital of *Brachylophosaurus* TMP 1990.104.001 has a circular pathologic depression, and should not be used for textural comparison. The lateral surface of the jugal process in *Acristavus gagslarsoni* (MOR 1155) is smooth and concave, matching the Malta specimens. MOR 2919 lacks the anteriorly directed sheet of bone in the posterodorsal corner of the orbit and jugal process depression present in *Acristavus* sp. (UMNHVP 16607).

The squamosal processes of the MOR 2919 postorbitals were fractured at their bases and reattached; these breaks combined with diagenetic deformation make it difficult to determine whether the squamosal processes projected somewhat dorsally as in most hadrosaurines, or remained in line with the anterior postorbitals as in *Brachylophosaurus* (CMN 8893, MOR 794, TMP 1990.104.001) and *Acristavus* (MOR 1155) [3]. The squamosal processes of MOR 2919 are broken posteriorly before the contact with the squamosal. The sutures between the postorbitals and parietal are completely fused and obscured.

#### Squamosal

The prequadratic process is stout and as mediolaterally wide as it is dorsoventrally high (Fig 13B), similar to that of subadult *Brachylophosaurus* (MOR 1071-7-13-99-87-H), but it is shorter and wider than that of adult *Brachylophosaurus* (CMN 8893, MOR 794, MOR 1071-7-7-98-86) and *Acristavus* (MOR 1155). The prequadratic processes of MOR 2919, *Acristavus* (MOR 1155), and *Brachylophosaurus* (MOR 1071-7-7-98-86, MOR 1071-7-13-99-87-H) are strongly anteroposteriorly compressed. Outside of Brachylophosaurini, the prequadratic process is nearly twice as dorsoventrally high as it is mediolaterally wide, and is subcircular in cross section rather than compressed (e.g. *Prosaurolophus* MOR 447-7-27-7-6). The prequadratic processes in *Maiasaura* specimens YPM-PU 22405 and OTM F138 are not preserved well enough for comparison. The postquadratic process in MOR 2919, *Acristavus* (MOR 1155), and *Brachylophosaurus* (MOR 1071-7-7-98-86) is more mediolaterally compressed than in *Maiasaura* or *Prosaurolophus*.

The articulation for the squamosal process of the postorbital extends to a point on the squamosal above the middle of the quadrate cotylus in MOR 2919, some *Brachylophosaurus* (FMNH PR 862), and Brachylophosaurini indet. (UCMP 130139), and extends just posterior to the middle in other *Brachylophosaurus* (MOR 794, MOR 1071-7-7-98-86). The squamosal process of the postorbital extends just anterior to the middle of the quadrate cotylus in *Acristavus*.

Posteriorly, the parasagittal crest of the parietal appears to bifurcate, similar to the condition in *Acristavus*. In MOR 2919 and *Acristavus*, this apparent bifurcation is actually composed of the posteromedial processes of the squamosals, which contact each other directly (Fig 3C and 3D). The posteromedial processes are broken off of the main bodies of both squamosals of MOR 2919, but a fragment of the left process remains attached to the parietal, with the suture to the parietal fused and nearly obliterated. On the right side, the squamosal was broken roughly at the fused suture. In *Brachylophosaurus*, the squamosals are separated by a narrow (MOR 1071-7-7-98-86) or thickened (CMN 8893; [23]) extension of the parasagittal crest. In *Maiasaura* OTM F138, the parasagittal crest inserts partially between the posteromedial processes of the squamosals so they are separated anteriorly but not posteriorly; this area is broken in the *Maiasaura* holotype YPM-PU 22405.

#### Quadrate

**Fig 14:**
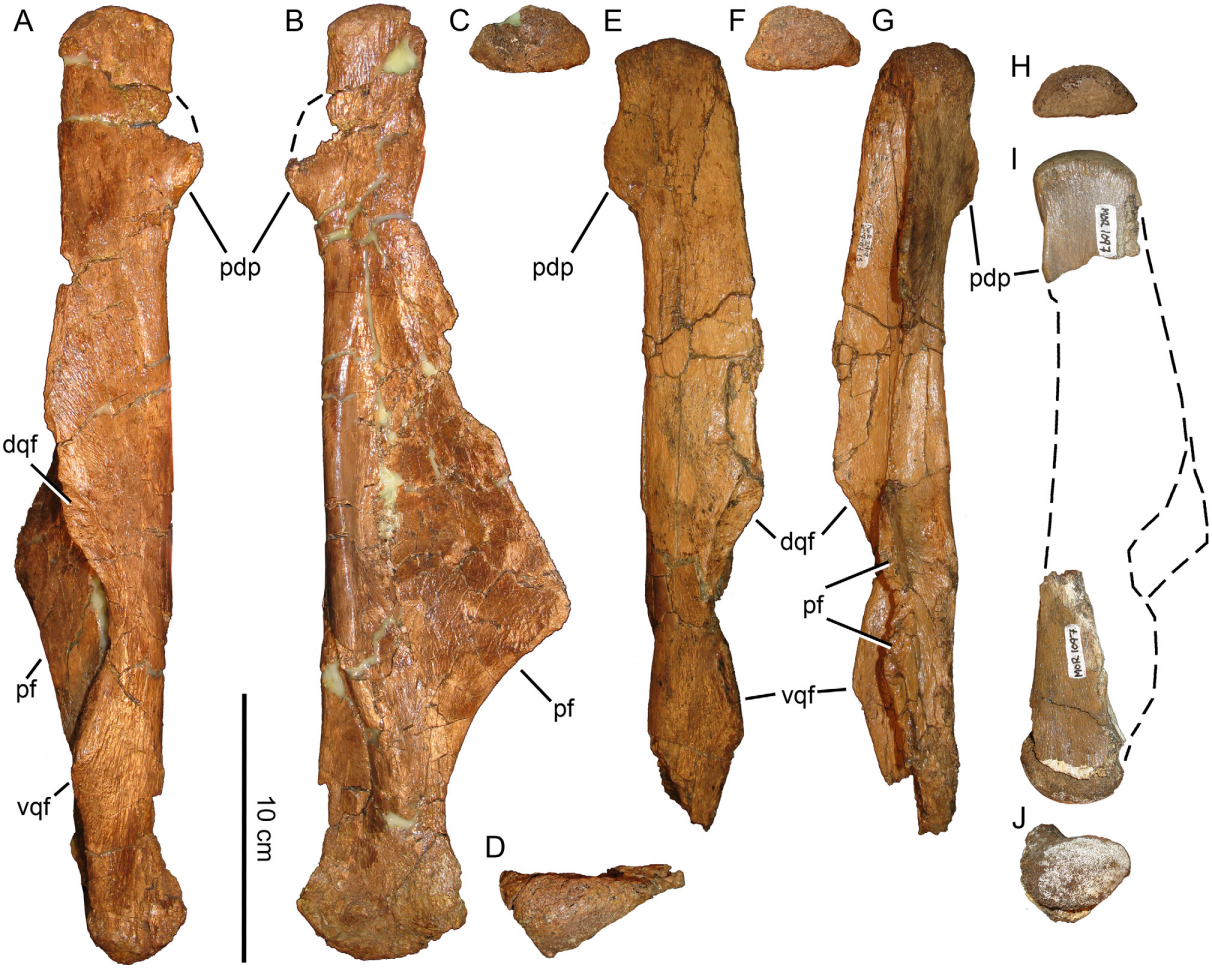
*Probrachylophosaurus bergei* gen. et sp. nov. quadrates. (A-D) Adult, MOR 2919, left quadrate; (A) lateral view; (B) medial view; (C) dorsal view of squamosal condyle; (D) ventral view of surangular condyle. (E-G) Adult, MOR 2919, right quadrate with broken pterygoid flange; (E) lateral view; (F) dorsal view of squamosal condyle; (G) medial view. (H-J) Subadult, MOR 1097, right quadrate; (H) dorsal view of squamosal condyle; (I) lateral view with missing portion reconstructed with dashed line; (J) ventral view of surangular condyle. Condyles are all oriented with the lateral side down. *Abbreviations*: *dqf*, dorsal quadratojugal flange; *pdp*, posterodorsal process; *pf*, pterygoid flange; *vqf*, ventral quadratojugal flange.

The posterodorsal process (also termed the quadrate buttress [3] or squamosal buttress [29]) below the squamosal condyle is slightly more enlarged than in *Brachylophosaurus*. In MOR 2919 the process is 4 cm high and extends 1 cm posteriorly (Fig 14). In *Brachylophosaurus* (CMN 8893, FMNH PR 862, MOR 794, MOR 1071-8-13-98-559-D) the posterodorsal process is more pointed, with its base a maximum of 3 cm high and extending a maximum of 1 cm posteriorly. The posterodorsal process in Brachylophosaurini indet. (UCMP 130139) is more similar to that of *Brachylophosaurus* than to MOR 2919. The posterodorsal process in *Acristavus* (MOR 1155) is low and gently convex, with its base 2 cm high and extending 3 mm posteriorly. In *Maiasaura* (YPM-PU 22405 and OTM F138) the posterodorsal process is elongated (3–3.5 cm high) as in MOR 2919, but low, extending only 3–5 mm posteriorly, as in *Acristavus*. The posterodorsal process in *Prosaurolophus* has an elongate base 2.5 cm high and extends 3 mm posteriorly, resembling that of *Maiasaura* and *Acristavus*. In *Gryposaurus notabilis* ROM 873 the posterodorsal process is large, convex, and continuous with the posterior margin of the squamosal condyle; in the *G*. *notabilis* holotype CMN 2278 the posterodorsal process is less pronounced posteriorly, but is vertically straight and continuous with the posterior margin of the condyle. The presence or absence of the posterodorsal process (squamosal buttress) is a character (Q5, character 120) in the matrix of Prieto-Márquez [29]; the similarities in size and shape of the posterodorsal process between *Probrachylophosaurus* and *Brachylophosaurus*, and their differences compared to other taxa such as *Gryposaurus*, suggest that the “presence” character state could be split into additional character states for greater phylogenetic resolution.

The dorsal half of the quadrate curves slightly posteriorly in *Brachylophosaurus* (CMN 8893, FMNH PR 862, MOR 794, MOR 1071-8-13-98-559-D) [33], Brachylophosaurini indet. (UCMP 130139), and *Maiasaura*, but is straight in MOR 2919 and *Acristavus*. In *Brachylophosaurus* (MOR 1071-8-13-98-559-D) the flange above the dorsal quadratojugal articulation is much larger anteriorly than the ventral flange to accommodate the posterior deflection of the dorsal quadrate, so that the dorsal quadratojugal articulation is directly above the ventral in life position. Specimens with straight quadrates (MOR 2919 and *Acristavus*) have shallow quadratojugal embayments, with the dorsal flange only slightly larger than the ventral flange. Although *Maiasaura* possesses the posterior deflection of the dorsal quadrate, its quadratojugal flanges are nearly equal in size.

There may have been a paraquadratic foramen present in MOR 2919. Cuthbertson and Holmes [23] noted that the quadratojugal embayment of the quadrate of the *Brachylophosaurus* holotype CMN 8893 possesses roughened dorsal and ventral articular facets for the quadratojugal, and interpreted the smooth surface between the dorsal and ventral facets as evidence supporting a paraquadratic foramen. Cuthbertson and Holmes [23] also noted that a paraquadratic foramen is not visible in other specimens of *Brachylophosaurus* (MOR 794 and TMP 1990.104.001), although the separated dorsal and ventral articular facets are present on the quadrate of TMP 1990.104.001 (and the disarticulated quadrate MOR 1071-8-13-98-559-D). The authors hypothesized that a paraquadratic foramen may have been variably present or absent in different individuals of *Brachylophosaurus*. The embayment for the quadratojugal in MOR 2919 has roughened articular surfaces for the dorsal and ventral quadratojugal contacts, and is smooth in between them, similar to the state in *Brachylophosaurus* that was interpreted by Cuthbertson and Holmes [23] as a small paraquadratic foramen. The quadrates of the *Acristavus* holotype MOR 1155, *Prosaurolophus* MOR 447-8-4-7-2, and *Gryposaurus* MOR 2573 also have a small smooth area separating the dorsal and ventral articular facets. Thus, if this smooth area is evidence of a paraquadratic foramen, then this foramen is widespread among hadrosaurines. Because a paraquadratic foramen is coded as absent for all hadrosaurines and lambeosaurines in the analysis of Prieto-Márquez [29] (character 189), the presence of a smooth area between the dorsal and ventral articular facets may not be indicative of a paraquadratic foramen.

The pterygoid flange has a slightly roughened surface where it articulates with the pterygoid; its extent is similar to that in *Brachylophosaurus* (FMNH PR 862, MOR 1071-8-13-98-559-D); the dorsal margin of the articulation in MOR 2919 is defined by a slight ridge also present in *Brachylophosaurus*. The articulation surfaces in *Acristavus* and *Maiasaura* (OTM F138) are very subtly roughened.

#### Quadratojugal

No quadratojugals of MOR 2919 are preserved, so it is unknown whether they had a crescentic or noncrescentic posterior margin, and whether this may have contributed to the margin of a paraquadratic foramen. Some specimens of *Brachylophosaurus* (holotype CMN 8893) have a noncrescentic posterior margin of the quadratojugal and the interpreted presence of a paraquadratic foramen, whereas other specimens (MOR 794, MOR 1071-7-21-98-344) have a crescentic posterior margin of the quadratojugal, and no visible paraquadratic foramen [23].

### Skull—Braincase

**Fig 15:**
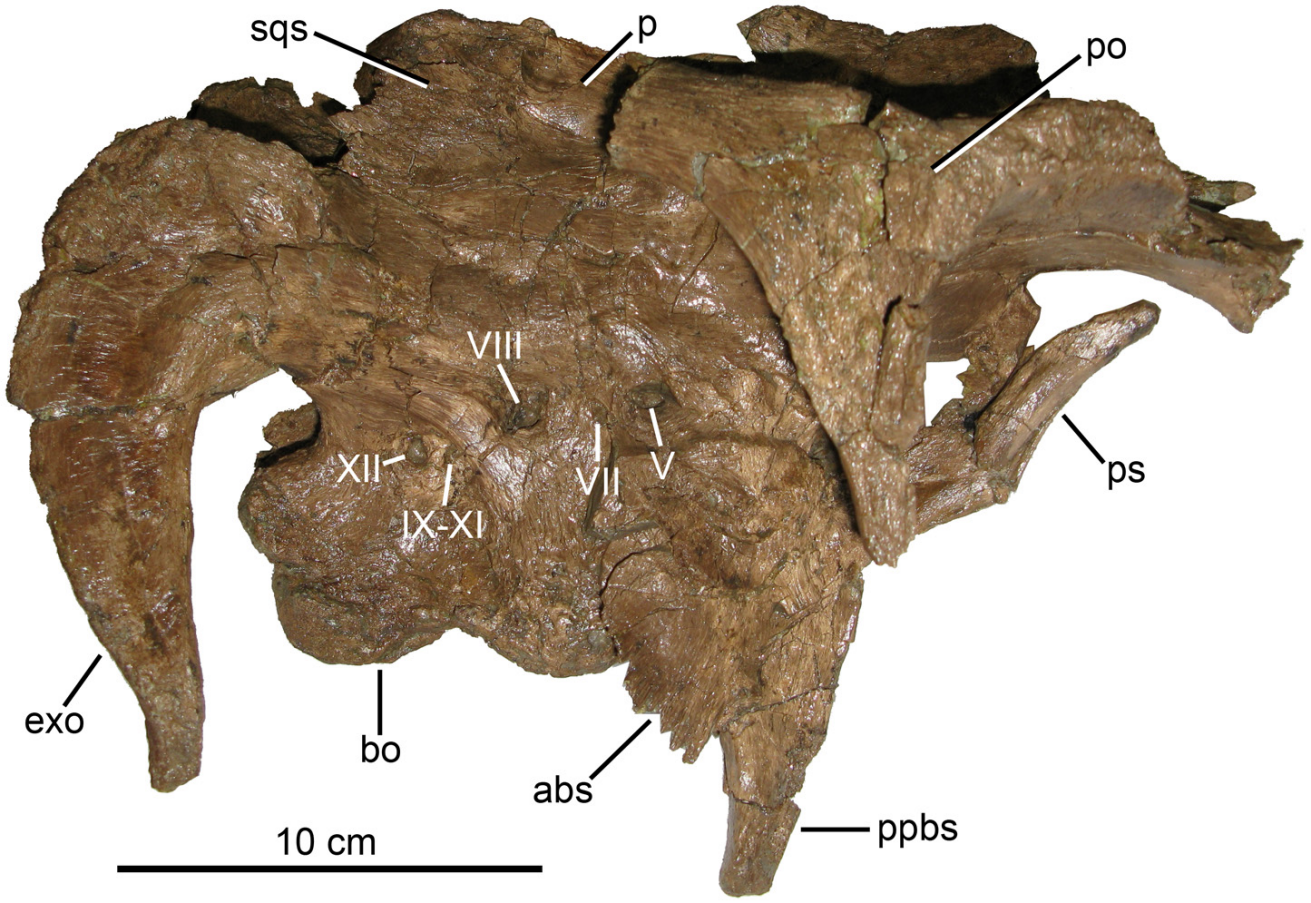
*Probrachylophosaurus bergei* gen. et sp. nov. braincase. MOR 2919, right lateral view. *Abbreviations*: *abs*, alar process of basisphenoid; *bo*, basioccipital; *exo*, exoccipital; *p*, parietal; *po*, postorbital; *ppbs*, pterygoid process of basisphenoid; *ps*, parasphenoid (broken and deflected dorsally); *sqs*, squamosal suture on parietal, broken; *V-XII*, cranial nerve foramina.

The braincase elements are fully fused and suture lines are either in the process of being remodeled or are already obliterated (Fig 15), making the contacts of individual elements difficult to define, as in CMN 8893 [23]. Crushing has obscured some cranial nerve foramina. Aside from the features noted below, the overall morphology conforms to that of *Brachylophosaurus* [1].

The large alar process of the basisphenoid is extremely thin, as in *Brachylophosaurus* (MOR 1071-7-7-98-86); the proximal-distal striations are more prominent in MOR 2919. The posteroventral process of the basisphenoid is more pronounced in MOR 2919 than in *Maiasaura* (OTM F138), *Acristavus*, and some *Brachylophosaurus* specimens (MOR 1071-7-7-98-86), but smaller than in others (CMN 8893; [23]). In *Brachylophosaurus* (MOR 1071-7-7-98-86) and *Acristavus* the pterygoid processes of the basisphenoid are directed nearly entirely laterally, and slightly anteriorly and ventrally. In MOR 2919 and *Maiasaura* (OTM F138) the pterygoid processes are directed more ventrally than laterally, and slightly posteriorly. Much of this difference may be diagenetic; the braincases of MOR 1071-7-7-98-86 and MOR 1155 are dorsoventrally compressed, MOR 2919 is laterally compressed, and OTM F138 is slightly laterally compressed. The direction of compression likely determines the relative degree of lateral or ventral orientation of the pterygoid processes, and may determine whether the pterygoid processes are directed slightly anteriorly or posteriorly. Thus, the angle between the pterygoid processes of the basisphenoid is not a useful phylogenetic character without first accounting for diagenetic influence on all specimens.

In MOR 2919 as in *Brachylophosaurus* (CMN 8893, MOR 1071-7-7-98-86), the opening for cranial nerve VII is slightly posteroventral to cranial nerve V, rather than directly posterior as in *Acristavus* (UMNHVP 16607) [3, 23]. The groove in the opisthotic leading from cranial nerve VIII to the exoccipital is much wider and deeper in *Brachylophosaurus* (MOR 1071-7-7-98-86) than in MOR 2919. The paraoccipital processes of the exoccipitals barely curve anteriorly, similar to *Brachylophosaurus* (CMN 8893, MOR 794), and unlike the strong anterior curvature in *Acristavus*. The region of the posterior-most contacts between the squamosals, supraoccipital, and exoccipitals is broken, but if intact the exoccipital roof would have been short as in *Brachylophosaurus* (MOR 1071-7-7-98-86).

#### Palate

The pterygoids, ectopterygoids, vomers, and palatines are not preserved in MOR 2919. A partial palatine in MOR 1097 is morphologically similar to that of *Brachylophosaurus* (MOR 1071-7-26-99-221).

### Skull—Mandible

#### Predentary

**Fig 16:**
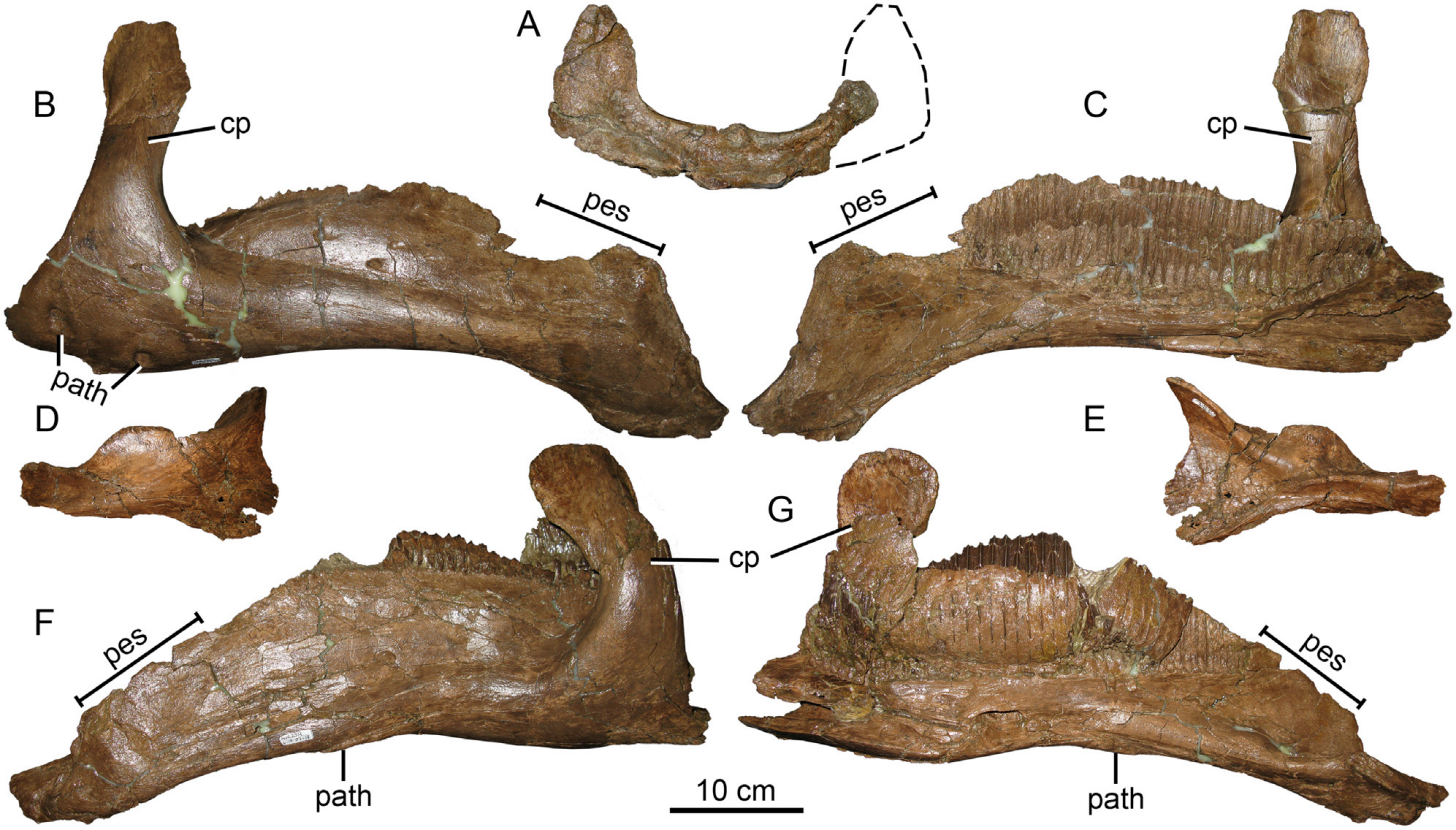
*Probrachylophosaurus bergei* gen. et sp. nov. mandible. MOR 2919 (A) predentary, ventral view; (B) right dentary, lateral view; (C) right dentary, medial view; (D) right surangular, lateral view; (E) right surangular, medial view; (F) left dentary, lateral view; (G) left dentary, medial view. The anterior portion of the right dentary was crushed mediolaterally, flattening the symphyseal region into a vertical orientation. The coronoid process of the left dentary was fractured, displacing the coronoid process anteromedially. *Abbreviations*: *cp*, coronoid process; *path*, pathology; pes, proximal edentulous slope.

The predentary is very poorly preserved, but its relative dimensions are similar to those of *Brachylophosaurus* (MOR 1071-7-28-98-299), being roughly twice as wide mediolaterally as anteroposteriorly, and having a squared anterolateral corner (Fig 16A). The denticles are not preserved, and the nutrient foramina are crushed and obscured.

#### Dentary

The dental lamina is extremely thin; in some regions there are ridges corresponding to the median carinae of the teeth, and in other areas the lamina is so thin that the median carinae are exposed, possibly due to postmortem abrasion of the lamina (Fig 16). In *Brachylophosaurus* (FMNH PR 862), *Acristavus*, and *Maiasaura*, the dental laminae are similarly ridged superficial to the carinae. The left dentary of MOR 2919 is larger than any other brachylophosaurin dentary (Table 1), and preserves the complete series of 43 tooth rows. *Brachylophosaurus canadensis* dentaries contain up to 39 tooth rows (CMN 8893; [33]); other specimens have 33 tooth rows (MOR 1071-8-98-X) and 36 tooth rows (FMNH PR 862). Most dentary tooth rows in MOR 2919 contribute two teeth to the occlusal surface, which is less concave than in other Brachylophosaurini. Typically, each tooth row corresponds to one medial dental foramen. However, the 3^rd^ and 4^th^ anterior tooth rows share the same dental foramen; because the number of tooth rows in hadrosaurids increases ontogenetically [37], this could represent the origin of an additional tooth row, and an additional foramen would develop later. The ventromedial groove that leads to the Meckelian canal originates below or anterior to the first tooth row in *Brachylophosaurus* (FMNH PR 862, MOR 1071-8-98-W, CMN 8893) [23] and the *Maiasaura* holotype YPM-PU 22405, but several rows posterior to the first tooth row in MOR 2919, *Acristavus*, and *Maiasaura* OTM F138.

The dentary teeth have a prominent median carina with no secondary carinae. The median carinae are straight or rarely extremely subtly sinusoidal in MOR 2919, but many median carinae are subtly sinusoidal in at least one *Brachylophosaurus* (FMNH PR 862). The edges of the crowns have faint papillae that are much less prominent than the papillae on the maxillary teeth.

MOR 2919 has five anterolateral dental foramina. *Brachylophosaurus canadensis* may be intraspecifically variable: CMN 8893 has four anterolateral dental foramina [23], but FMNH PR 862 has either four or five anterolateral dental foramina (preservation may be obscuring some foramina). *Maiasaura* is also intraspecifically variable: YPM-PU 22405 has five foramina, but OTM F138 only has four foramina. *Acristavus* (MOR 1155) has four foramina.

MOR 2919, *Maiasaura* (YPM-PU 22405, OTM F138), and *Acristavus* (MOR 1155) possess a relatively shorter proximal edentulous slope of the dentary than in *Brachylophosaurus* (CMN 8893, FMNH PR 862, MOR 1071-8-98-W). In *Brachylophosaurus* and *Maiasaura*, the edentulous slope is actually horizontal for a few centimeters just anterior to the first tooth row before sloping anteroventrally; in MOR 2919 and *Acristavus* the anteroventral slope begins immediately anterior to the first tooth row.

**Fig 17:**
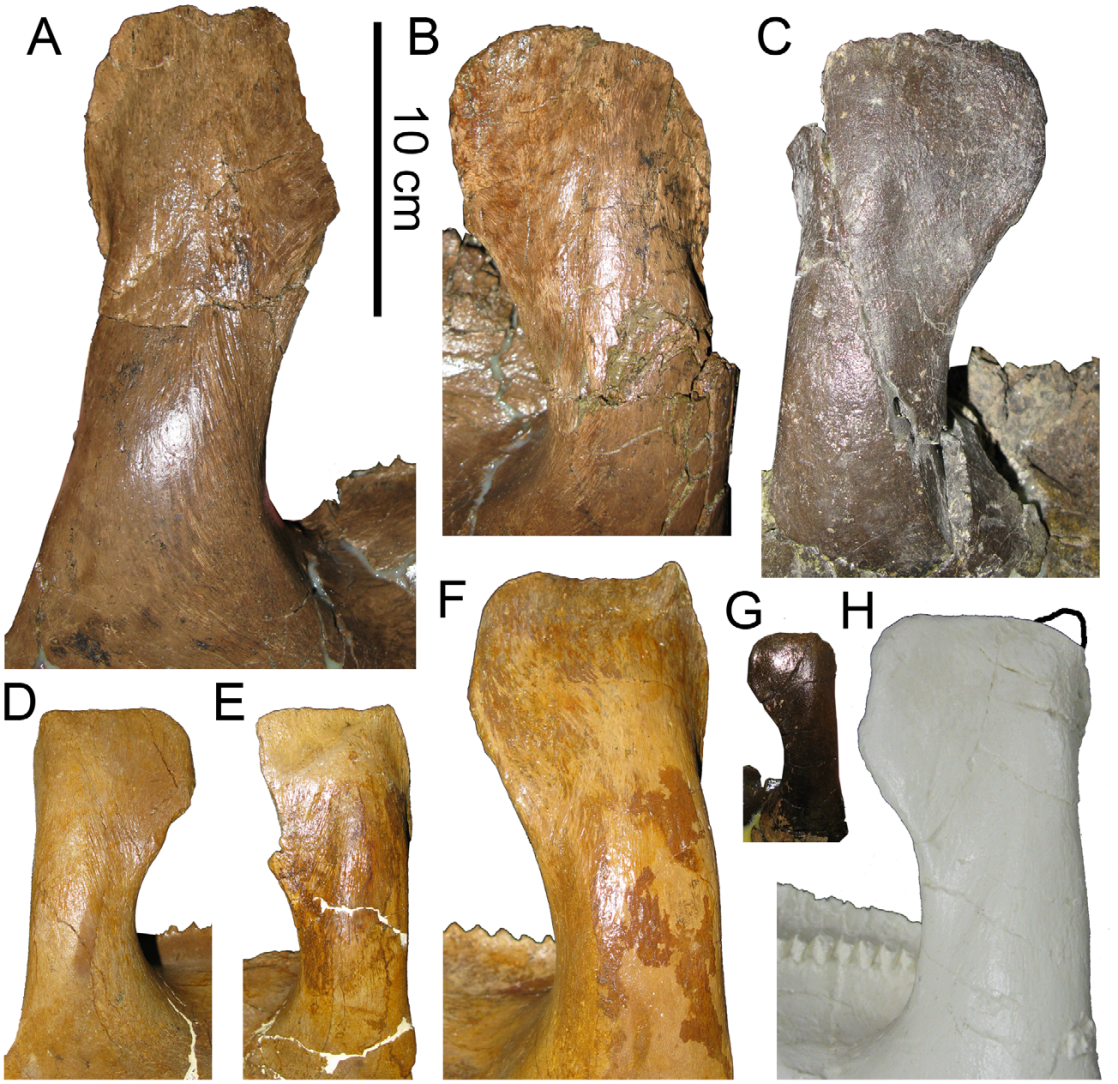
Taxonomic distribution and ontogenetic development of caudodorsal point on coronoid process of dentary. Caudodorsal point absent in: *Probrachylophosaurus* gen. nov. adult, MOR 2919, (A) right coronoid process, anterior margin broken, (B) left coronoid process, dorsal margin broken and entire process fractured and misaligned; (C) *Acristavus*, MOR 1155, right coronoid process, posterior margin broken. Caudodorsal point develops ontogenetically and with individual variation in *Brachylophosaurus*: (D) subadult, MOR 1071-8-1-99-313, right coronoid process; (E) subadult, MOR 1071-7-10-98-179, left coronoid process, anterior margin broken; (F) adult, MOR 1071-8-98-X, left coronoid process. Caudodorsal point develops ontogenetically in *Maiasaura*: (G) juvenile, MOR 547-W-55-2P, left coronoid process; (H) adult, OTM F138, left coronoid process of cast. The original description of OTM F138 [36] includes a photograph of the left dentary with a complete caudodorsal point, but the cast at MOR does not include the caudodorsal point, suggesting that when the resin was poured into the mold, it did not completely fill this area. The missing caudodorsal point is indicated by a black outline traced from the left dentary photograph in Trexler [36].

*Maiasaura* (OTM F138, ROM 44770; area broken in holotype YPM-PU 22405) and *Brachylophosaurus* (CMN 8893, FMNH PR 862, MOR 1071-7-7-02-62-D, MOR 1071-8-98-X) are unique in possessing a dentary coronoid process with a horizontally straight dorsal margin leading to a sharp caudodorsal point (Fig 17). MOR 2919 has the horizontally straight dorsal margin, although it is thin and slightly broken, and appears to lack the caudodorsal point. The caudodorsal point also appears absent in *Acristavus* (MOR 1155), but the posterodorsal margin of the coronoid process is slightly broken, and so the presence or absence of a caudodorsal point cannot be confirmed. The caudodorsal point develops ontogenetically in *Maiasaura* and *Brachylophosaurus*; juveniles have the straight dorsal margin with no point (*Maiasaura* MOR 547-W-55-2P, *Brachylophosaurus* MOR 1071-8-1-99-313), similar to the state of the stratigraphically older MOR 2919. The subadult *Probrachylophosaurus* specimen, MOR 1097, does include a coronoid process, but the dorsal end is broken, so the size and shape of its margins cannot be observed. The presence or absence of a caudodorsal point may be a useful ontogenetic taxonomic character, but because the point is easily taphonomically broken, it may be difficult to determine whether the absence of a caudodorsal point is true or taphonomic in certain specimens.

#### Surangular

The surangular (Fig 16D and 16E) conforms in all respects to the morphology described for *Brachylophosaurus* [1, 23]. The surangular of *Acristavus* is also consistent with that of *Brachylophosaurus* [3]. The articular, splenial, and angular are not preserved.

### Postcrania

In nearly all respects, the postcrania of MOR 2919 are consistent with those of *Acristavus* and *Brachylophosaurus*.

#### Vertebrae

**Fig 18:**
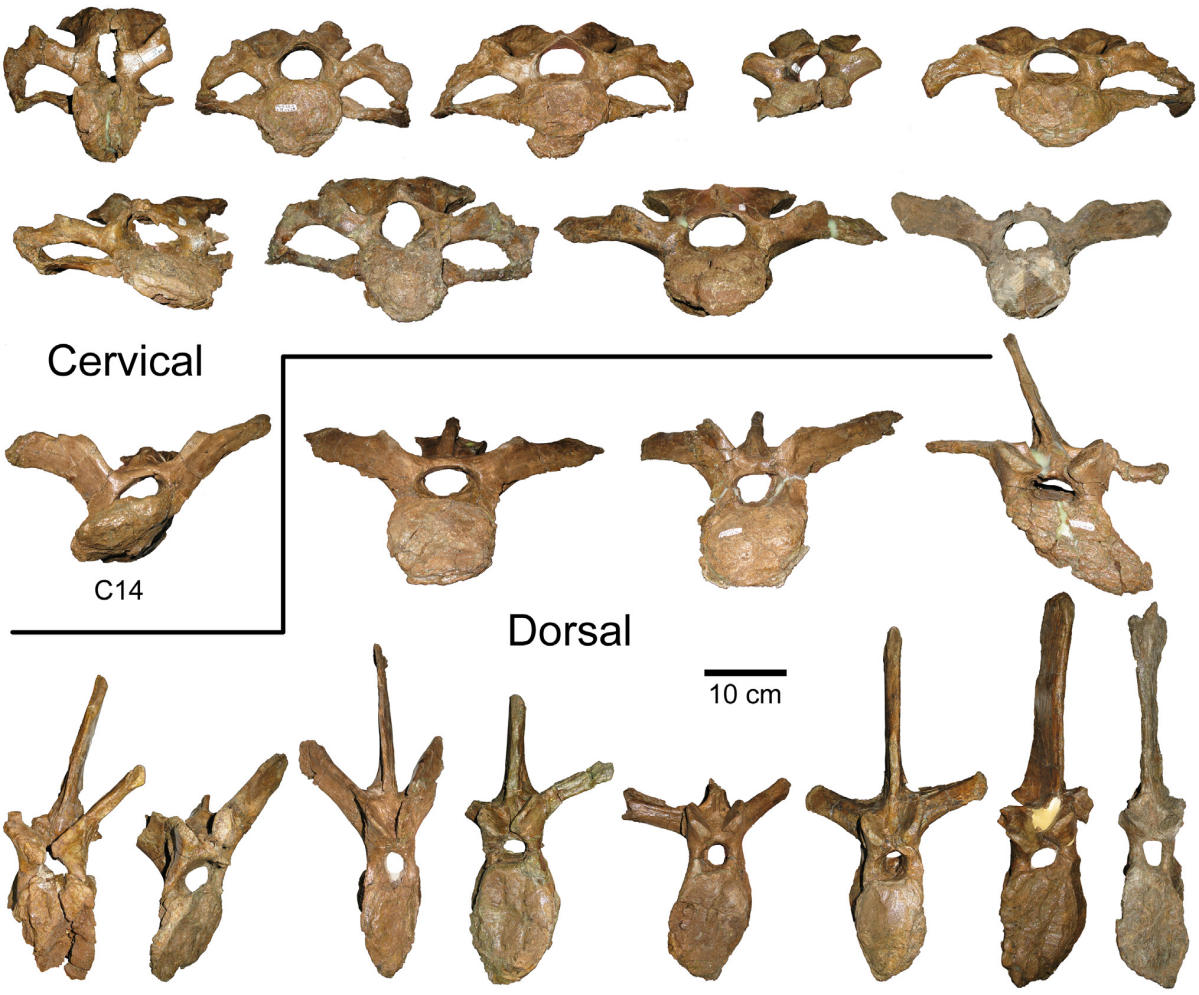
*Probrachylophosaurus bergei* gen. et sp. nov. cervical and dorsal vertebrae. MOR 2919 vertebrae are pictured in anterior view in their approximate order within their series. Serial position of vertebrae is not listed because the complete series of cervicals and dorsals were not preserved, and so their exact serial positions cannot be determined with confidence. However, the last cervical vertebra pictured, “C14”, is consistent with the morphology of cervical 14 in CMN 8893, the holotype of *Brachylophosaurus canadensis* [23]. Atlas fragments and other vertebral fragments not pictured.

**Fig 19:**
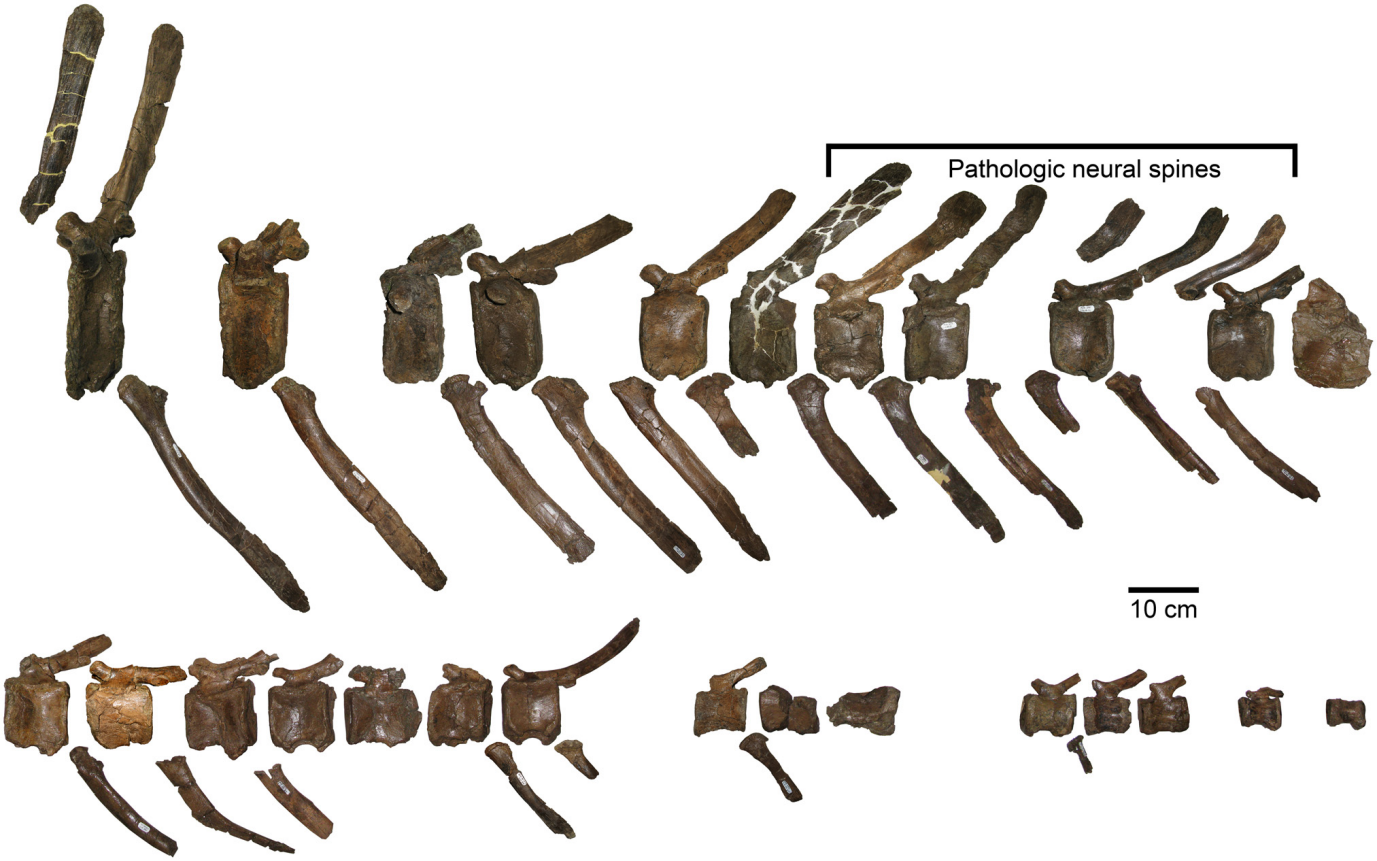
*Probrachylophosaurus bergei* gen. et sp. nov. caudal vertebrae. MOR 2919 vertebrae, neural spines, and chevrons are pictured in left lateral view in their approximate order within their series. Due to the disarticulated and incomplete nature of this caudal vertebral series, exact serial positions cannot be determined with confidence. Gaps between pictured vertebrae indicate noticeably missing segments of multiple vertebrae; additional vertebrae may be missing between any two vertebrae pictured here. Additional caudal vertebral fragments not pictured.

In all collected vertebrae of MOR 2919 (cervicals, dorsals, and caudals), the neural arches are fully fused to the centra, with the suture lines nearly completely obliterated (Figs 18 and 19). Small cervical, dorsal, and caudal vertebrae from the MOR 1071 bonebed either have unfused neural arches found disarticulated from their centra, or are unfused but remained connected. A broken surface on an adult (determined by relative size) *Brachylophosaurus canadensis* dorsal vertebra MOR 1071-8-18-98-593 demonstrates that internal fusion and remodeling are complete, but the external surface of the suture line is still being remodeled. A dorsal vertebra of MOR 2919 from a similar serial position exhibits very faint remodeled bone texture along the location of sutural fusion.

MOR 2919 includes atlas fragments, 10 well-preserved cervical vertebrae, and fragments of additional cervical vertebrae, for a total of at least 12 cervical vertebrae (Fig 18). One cervical is morphologically consistent with cervical 14 of CMN 8893, the holotype of *Brachylophosaurus canadensis* [23]. Because the cervical series of MOR 2919 is incomplete, it cannot be determined whether this is actually the 14^th^ vertebra in the series. Prieto-Márquez [25] describes the articulated *B*. *canadensis* specimen MOR 794 as having only 13 cervical vertebrae, but Cuthbertson and Holmes [23] note that they may differ in their interpretation of whether the 14^th^ vertebra is a cervical or a dorsal.

#### Pelvis and hindlimb

**Fig 20:**
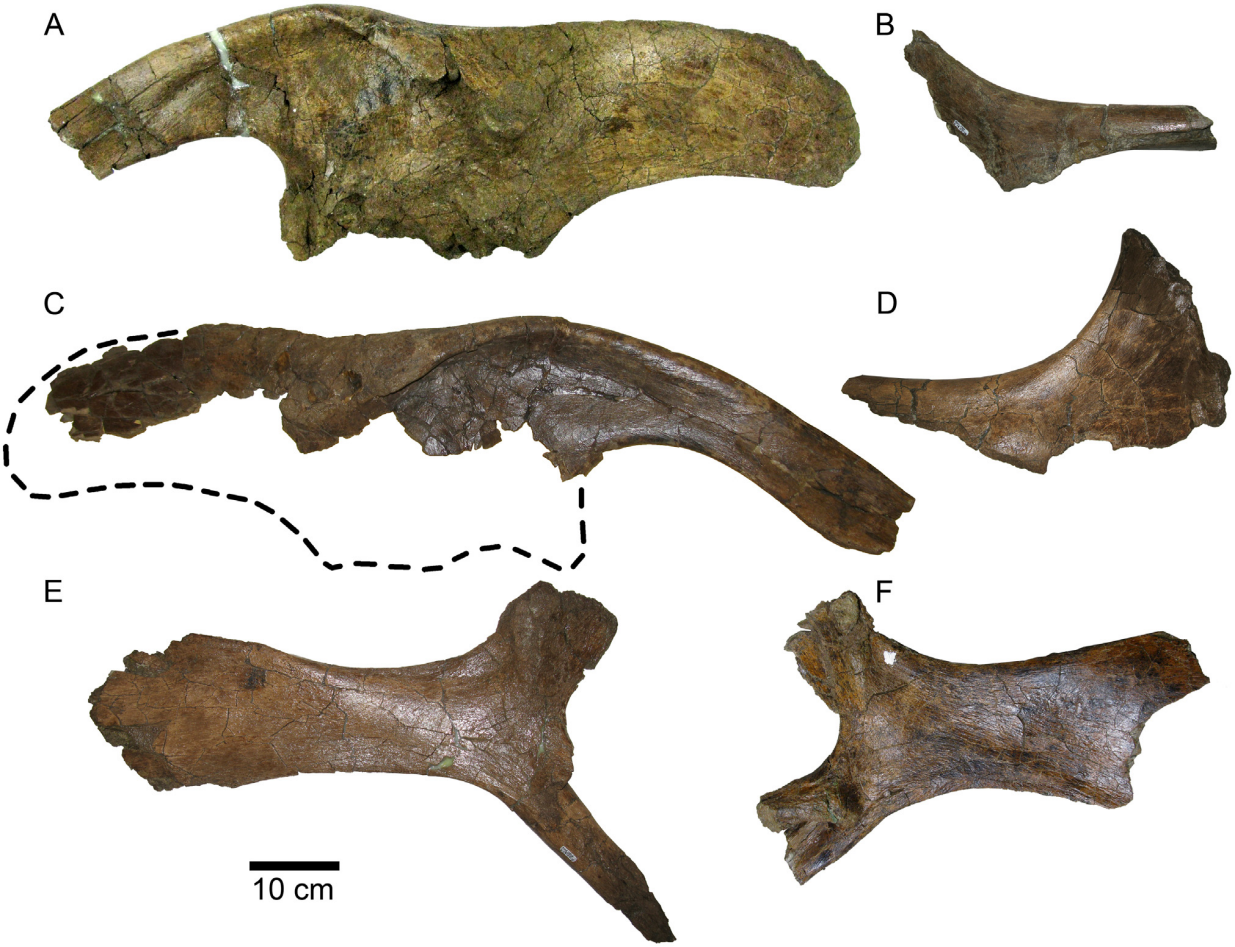
*Probrachylophosaurus bergei* gen. et sp. nov. pelvic elements. MOR 2919, all elements in lateral view; (A) left ilium; (B) partial proximal left ischium; (C) right ilium with dashed line indicating missing area; (D) partial proximal right ischium; (E) left pubis; (F) right pubis.

**Fig 21:**
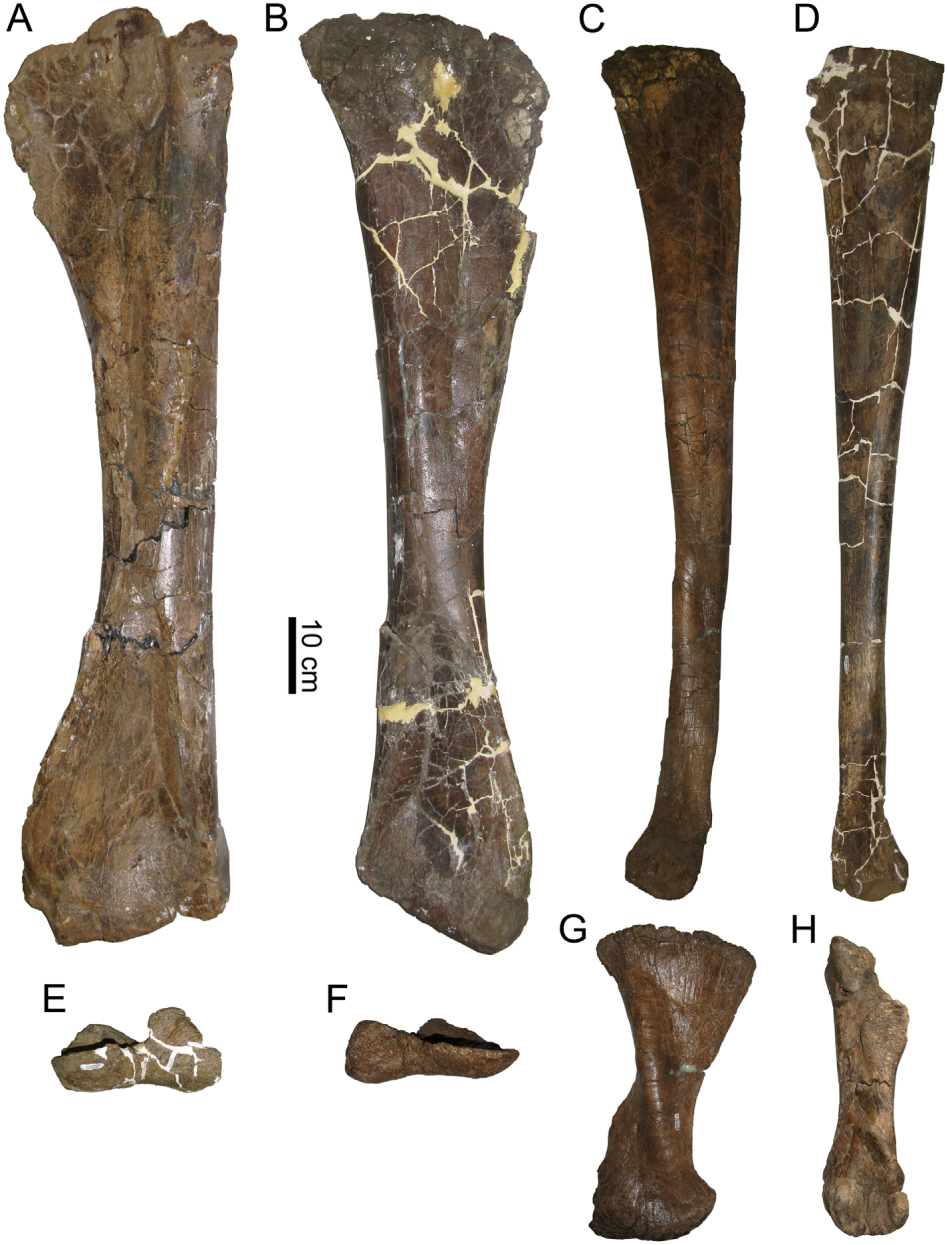
*Probrachylophosaurus bergei* hindlimb elements. MOR 2919 (A) left tibia, lateral view; (B) right tibia, lateral view; (C) left fibula, lateral view; (D) right fibula, lateral view; (E) left astragalus, anterior view; (F) right astragalus, anterior view; (G) right metatarsal II, medial view; (H) right metatarsal IV, medial view.

In *Brachylophosaurus* MOR 1071 specimens, the ischial peduncle of the pubis flares at its tip. In *Acristavus* and MOR 2919, it is a more rounded enlargement (Fig 20). The lengths of the fully intact limb bones (tibia, fibula, and metatarsal II; Fig 21) are provided in Table 3.

### Pathologies

Pathologies are present on both dentaries, at least six caudal vertebrae, and two metatarsals. The dentaries of MOR 2919 possess a total of three pathologic circular depressions each surrounded by a ring of raised bone (Fig 16). Six middle caudal vertebrae of MOR 2919 exhibit neural spine pathologies consistent with various degrees of breakage and healing. The pathologies are limited to a single region of the middle of the tail (Fig 19); the caudal series is disarticulated and incomplete, so there may have been additional pathologic vertebrae within this region. The only two metatarsals recovered, the right II and IV, both exhibit pathologic rugose surface texture. The right metatarsal II has this rugose texture on much of its contact surface with metatarsal III, and nowhere else. The right metatarsal IV has the rugose texture concentrated on its medial contact surface with metatarsal III, as well as it distal posterolateral surface. Metatarsal III was not recovered, but presumably would have suffered from a similar pathology. The distal ends of both tibiae and both fibulae exhibit patches of pathologic surface texture, suggesting that both feet may have been similarly affected. The pathologies of MOR 2919 will be described and discussed in detail in a later paper.

### Summary of Morphologic Comparisons Among *Probrachylophosaurus* and Other Brachylophosaurins

In cases where cranial morphology can be directly compared between *Probrachylophosaurus*, *Acristavus*, *Brachylophosaurus*, and *Maiasaura*, *Probrachylophosaurus* is always more similar to *Acristavus* or *Brachylophosaurus* than it is to *Maiasaura*. Of the cranial elements well-preserved in the *Probrachylophosaurus* material, those with the most useful features for comparing brachylophosaurins are the jugal, lacrimal, nasal, frontal, squamosal, quadrate, braincase, and dentary.

The palatine process of the jugal in subadult *Probrachylophosaurus* (MOR 1097) is identical to the stratigraphically older *Acristavus*, but the palatine process of the jugal in adult *Probrachylophosaurus* (MOR 2919) is most similar to the stratigraphically younger *Brachylophosaurus*. The palatine process in *Maiasaura* is more similar to *Acristavus* and subadult *Probrachylophosaurus* than to *Brachylophosaurus* and adult *Probrachylophosaurus*. The caudoventral apex of the rostral process of the jugal is posterior to the caudodorsal apex in both *Probrachylophosaurus* and *Acristavus*; the apices are vertically aligned in *Brachylophosaurus*. *Maiasaura* possesses an intermediate condition. The ventral margin of the rostral process of the jugal is nearly straight in *Probrachylophosaurus*, *Acristavus*, and *Maiasaura*, but sigmoidal in *Brachylophosaurus*. The concavity of the posterior jugal margin is shallow in *Probrachylophosaurus*, *Acristavus*, and *Maiasaura*, but deeper in *Brachylophosaurus*. The medial excavation of the quadratojugal process of the jugal is shallowest in *Probrachylophosaurus*, less shallow in *Acristavus*, and deepest in *Brachylophosaurus* and *Maiasaura*. The mediolateral width of the lacrimal is more thick and robust in *Probrachylophosaurus* and *Acristavus* than in *Brachylophosaurus* and *Maiasaura*.

*Probrachylophosaurus* and *Brachylophosaurus* are united by having a solid nasal crest overhanging the dorsal skull roof; *Acristavus* lacks any crest, and *Maiasaura* has a crest formed by the dorsal projection of both the nasals and frontals [3, 5, 6, 33]. The nasal crest of the smaller adult (“slender”) *Brachylophosaurus* resembles a hypermorphic form of a *Probrachylophosaurus* crest, extending the crest posteriorly and laterally. The larger adult (“robust”) *Brachylophosaurus* crest continues this growth trajectory, with the posterolateral growth forming a flat paddle shape. These taxa have the same pattern in their nasofrontal articulation; subadult *Brachylophosaurus* and adult *Probrachylophosaurus* possess a nasofrontal articulation covering slightly over half of the dorsal frontal surface, whereas adult *Brachylophosaurus* have a larger nasofrontal articulation that covers the entire dorsal frontal surface.

The frontal contribution to the orbital margin is more similar in *Probrachylophosaurus* and *Acristavus* than either is to that of *Brachylophosaurus*. The prequadratic process of the squamosal in *Probrachylophosaurus* is more similar to that of subadult *Brachylophosaurus* than adult *Brachylophosaurus* or *Acristavus*. The postquadratic process of the squamosal is similar in *Probrachylophosaurus*, *Acristavus*, and *Brachylophosaurus*, and different from that of *Maiasaura*. The posterior end of the postorbital-squamosal articulation occurs just anterior to the middle of the quadrate cotylus in *Acristavus*, in the middle of the cotylus in *Probrachylophosaurus* and some *Brachylophosaurus* specimens (FMNH PR 862), and just posterior to the middle in other *Brachylophosaurus* (MOR 794, MOR 1071-7-7-98-86). In *Probrachylophosaurus* and *Acristavus*, the squamosals contact each other. In *Maiasaura*, the squamosals are partially separated by the parietal, and in *Brachylophosaurus*, the squamosals are completely separated by the parietal. The location of cranial nerve VII, as well as the curvature of the paraoccipital processes of the exoccipitals, are more similar in *Probrachylophosaurus* and *Brachylophosaurus* than either taxon is to *Acristavus*.

The size and shape of the posterodorsal process of the quadrate is similar in *Probrachylophosaurus* and *Brachylophosaurus*. The process in *Acristavus* is shorter and smaller; the process in *Maiasaura* is intermediate between *Probrachylophosaurus* and *Acristavus*. The quadrate posterior margin is straight in *Probrachylophosaurus* and *Acristavus*, but curved in *Brachylophosaurus* and *Maiasaura*.

The position of the ventromedial groove of the dentary of *Probrachylophosaurus* is more similar to *Acristavus* and *Maiasaura* OTM F138 than to *Brachylophosaurus* or *Maiasaura* YPM-PU 22405. The proximal edentulous slope of the dentary is shorter in *Probrachylophosaurus*, *Maiasaura*, and *Acristavus* than it is in *Brachylophosaurus*. The proximal-most portion of the edentulous slope is immediately sloping in *Probrachylophosaurus* and *Acristavus*, but horizontal in *Brachylophosaurus* and *Maiasaura*.

*Probrachylophosaurus* exhibits a unique combination of characters that make it morphologically intermediate between *Acristavus* and *Brachylophosaurus*. *Probrachylophosaurus* is most similar to *Acristavus* in overall morphology of the jugal and lacrimal. The morphologies of the frontal, quadrate, and squamosal are more similar to *Acristavus* in some attributes, but more similar to *Brachylophosaurus* in others. The braincase of *Probrachylophosaurus* is most similar to *Brachylophosaurus*. The presence of a posteriorly-oriented nasal crest also unites *Probrachylophosaurus* with *Brachylophosaurus*. The dentary is more similar to that of *Acristavus* than to *Brachylophosaurus*. These relationships of morphological similarities and differences were analyzed in more detail using phylogenetic analyses.

## Phylogenetic Analyses

### Analyses based on matrix of Prieto-Márquez [29]

*Probrachylophosaurus bergei* was coded into two versions of the matrix of Prieto-Márquez [29]. The first version included some recodings of previous taxa, with all characters included. The second version included these same recodings, but excluded some characters as detailed below.

The first analysis using the Prieto-Márquez [29] matrix included recodings of eight characters for *Acristavus* based on reexamination of the material. See Prieto-Márquez [29] for complete descriptions of characters and abbreviation codes (e.g., J4 is the fourth jugal character). Character 106 (J4), the relative position of the caudoventral apex of the rostral process of the jugal, was changed from state 1 to 0. *Probrachylophosaurus* is also coded as 0; *Brachylophosaurus* and *Maiasaura* are state 1. The quadrate of the *Acristavus* holotype (MOR 1155) was not coded into the original matrix, so it has been added here, replacing characters 116–121 (Q1-Q6)?????? with 011211. These are the same codings as the quadrate of *Probrachylophosaurus* and *Brachylophosaurus*; *Maiasaura* has states 001211. Character 259 (PB8), the length/width ratio of the ischial peduncle of the pubis, was recoded from 0 to 1 for *Acristavus*; *Probrachylophosaurus*, *Brachylophosaurus*, and *Maiasaura* are also state 1.

In *Brachylophosaurus*, Character 138 (F1) was originally coded as state 0, indicating the lack of bifurcation of the anterior frontal margin. However, this is only true for some adult specimens (e.g. GPDM JRF.65); the “slender” adult *Brachylophosaurus* MOR 1071-7-7-98-86 and subadults MOR 1071-7-13-99-87-I and MOR 1071-C-3-3 have bifurcated anterior frontal margins, so this was recoded to the multistate “01”.

When *Probrachylophosaurus bergei* is added to Prieto-Márquez’s [29] matrix, with extraneous taxa removed and some character states recoded (see above), but all characters included (S1 File), four most parsimonious trees were produced. The strict consensus tree recovers *Brachylophosaurus* and *Maiasaura* as sister taxa, with *Probrachylophosaurus* and *Acristavus* forming a basal polytomy within Brachylophosaurini (S2 Fig). Characters responsible for the position of *Probrachylophosaurus* included: 1 (dtth1), number of dentary tooth positions; 15 (mxth1), number of maxillary tooth positions; 36 (dt4), angle of the ventral rostral margin of the dentary; 44 (dt13), lack of caudodorsal point on coronoid process of dentary; 106 (j4), relative positions of caudodorsal and caudoventral apices of rostral process of jugal; 110 (j8), size of caudoventral flange of jugal; 113 (j11), ratio of caudal and rostral constrictions of jugal; and 143 (f6), contribution of the frontal to the orbital margin. The bootstrap 50% majority-rule consensus tree (5,000 replicates) finds 55% support for a *Probrachylophosaurus*-*Brachylophosaurus*-*Maiasaura* clade, with *Acristavus* as the basal member of Brachylophosaurini, and 64% support for *Brachylophosaurus* and *Maiasaura* as sister taxa.

Three of Prieto-Márquez’s [29] characters were then excluded from the second analysis. Characters 1 (dtth1) and 15 (mxth1) are based on the number of teeth in the dentary and maxilla, respectively. The number of teeth in hadrosaurid jaws increases ontogenetically [37, 38], so the high tooth counts in MOR 2919 are liable to be due to the specimen’s size and maturity rather than a true phylogenetic signal separating it from *Brachylophosaurus* and *Maiasaura*. Character 123 (pf2) describes the shape of the prefrontal contribution to the orbital margin. Many specimens of Brachylophosaurini examined in this study are intermediate between the character states, and so this character was excluded to avoid ambiguous state assignments.

Character 143 (f6) in Prieto-Márquez [29] is a modification of character 57 in Horner et al. [28]. The contribution of the frontal to the orbital margin is either present or absent in Horner et al. [28], but Prieto-Márquez [29] divides presence into two states, frontal exposed and forming part of orbital margin, or forming a narrow triangular apex that may or may not reach the orbital margin between the prefrontal and postorbital. This division is problematic because the width of the orbital contribution of the frontal is intraspecifically variable in *Brachylophosaurus* (MOR 1071 braincases, FMNH PR 862), and potentially may vary similarly in other taxa that currently have smaller sample sizes. Also, the distinction between the two states (frontal forming part of orbital margin or doing so through a narrow triangular apex) is not well defined. Prieto-Márquez [29] codes *Acristavus*, *Brachylophosaurus*, and *Maiasaura* as having the narrow triangular apex, but their contribution to the orbital margin can be more than 1 cm and not triangular. Thus, the frontal contribution to the orbital margin, when present, forms a continuum rather than two discrete states. End members are clear, but most brachylophosaurin specimens fall in between the end points, and so the original version of the character (simply presence or absence of frontal contribution to the orbital margin) in Horner et al. [28] is preferred. Consequently, character 143 (f6) was recoded to merge states 0 and 1, so there are only two states (presence or absence) rather than three.

**Fig 22:**
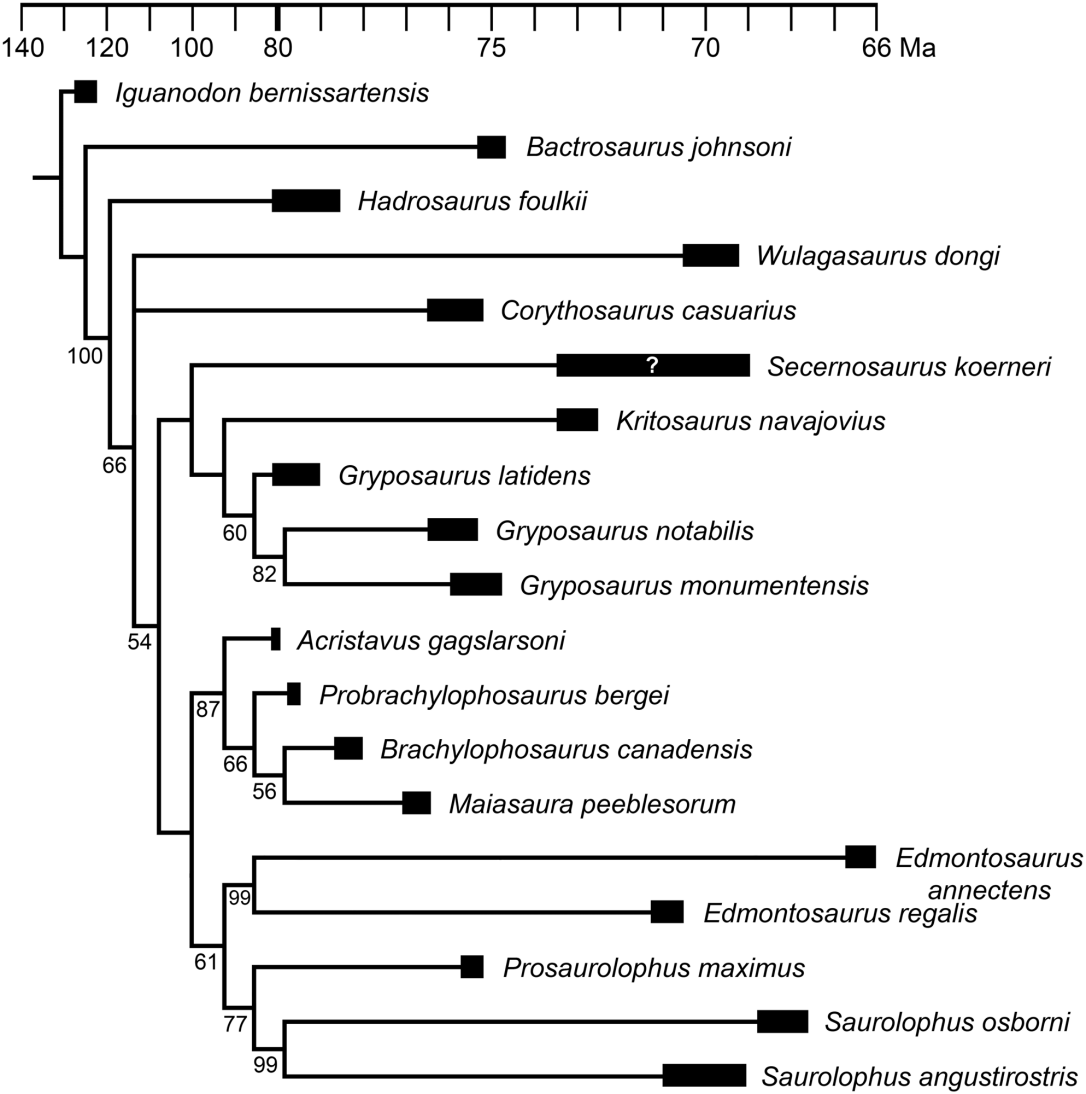
Time-calibrated cladogram of hadrosaurines based on Prieto-Márquez [29]. The strict consensus of two most parsimonious trees resulting from adding *Probrachylophosaurus bergei* gen. et sp. nov. to Prieto-Márquez’s [29] matrix with minor character recodings and exclusions as discussed in text was plotted using the age ranges for each taxon; branch lengths do not reflect the number of character state changes. Values on branches represent bootstrap support; branches without values had less than 50% support. Tree statistics: shortest tree length = 614, Consistency Index = 0.72, Retention Index = 0.68, Rescaled Consistency Index = 0.49. Age ranges of Brachylophosaurini are those recalibrated in Fig 1; age ranges of other taxa are approximate, and are based on unrecalibrated previously published dates [3, 7, 13, 15, 39–48]. Note that the age scale changes before and after 80 Ma. Also note that the age of *Bactrosaurus* has been variably proposed to be any time from late Turonian to early Maastrichtian in age [49–51].

After these exclusions and recodings (S2 File), two most parsimonious trees were produced. The strict consensus tree recovers *Acristavus* as the basal member of Brachylophosaurini, with *Brachylophosaurus* and *Maiasaura* as sister taxa, and *Probrachylophosaurus* in an intermediate position (Fig 22). *Probrachylophosaurus*, *Brachylophosaurus*, and *Maiasaura*, but not *Acristavus*, share one apomorphic character state: 134 (sq1) state 0, very short precotyloid process of the squamosal. *Probrachylophosaurus* differs from *Acristavus*, *Brachylophosaurus*, and *Maiasaura* in its state for three characters: 36 (dt4) state 2, angle of the ventral rostral margin of the dentary; 110 (j8) state 1, size of the caudoventral flange of the jugal; and 113 (j11) state 2, ratio of caudal and rostral constrictions of the jugal; these character states are homoplasies shared with non-brachylophosaurin hadrosaurine taxa. *Brachylophosaurus* and *Maiasaura* are united as sister taxa due to two characters: 44 (dt13) state 1, the autapomorphic presence of a caudodorsal point on the coronoid process of the dentary; and 106 (j4) state 1, relative positions of caudodorsal and caudoventral apices of the rostral process of the jugal. Compared to the previous result, this bootstrap 50% majority-rule consensus tree finds increased (66%, was 55%) support for a *Probrachylophosaurus*-*Brachylophosaurus*-*Maiasaura* clade, with *Acristavus* as the basal member of Brachylophosaurini, and decreased (56%, was 64%) support for *Brachylophosaurus* and *Maiasaura* as sister taxa.

### Analysis based on matrix of Gates et al. [3]

**Fig 23:**
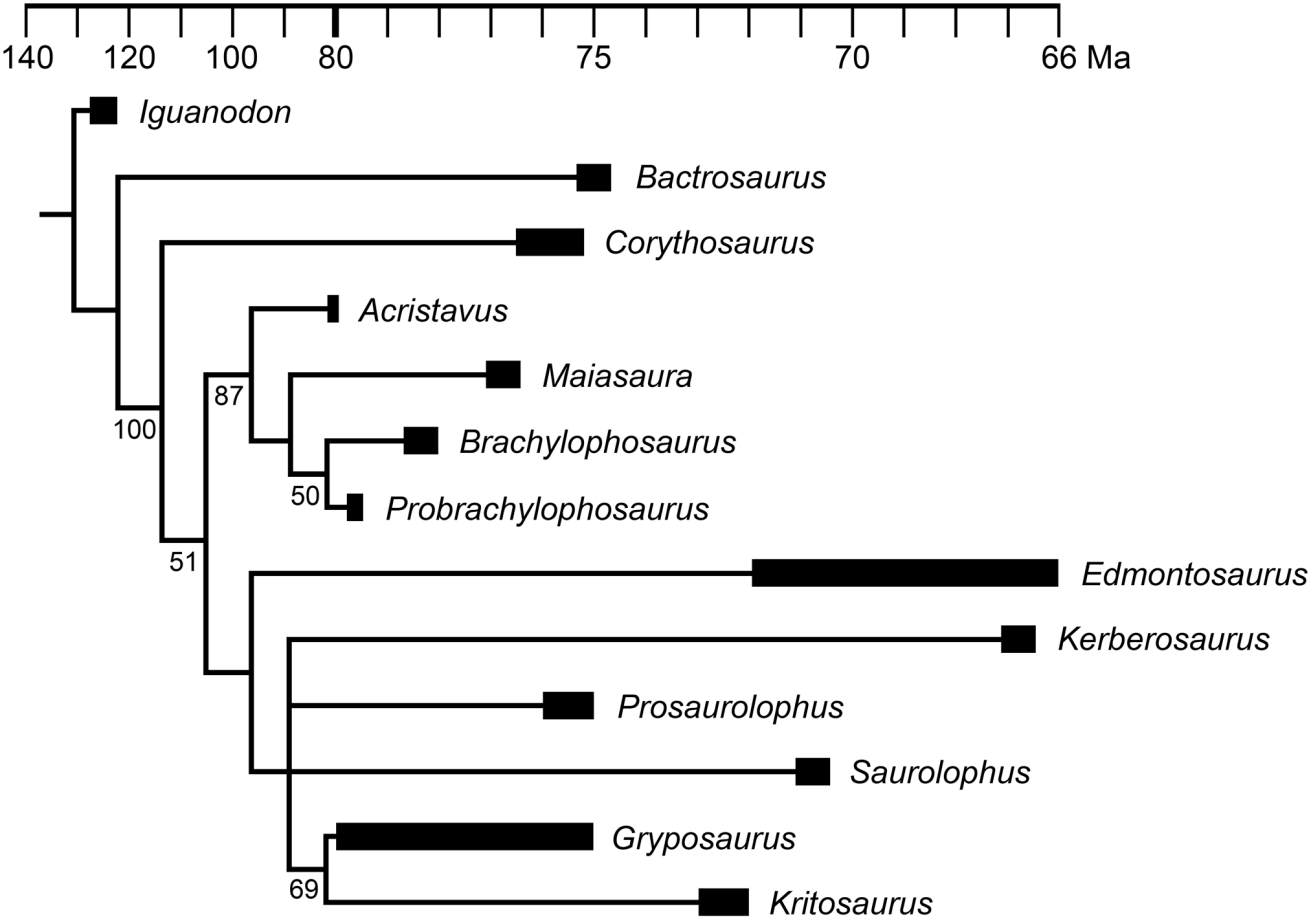
Time-calibrated cladogram of hadrosaurines based on Gates et al. [3]. The strict consensus of two most parsimonious trees resulting from adding *Probrachylophosaurus bergei* gen. et sp. nov. to Gates et al.’s [3] matrix was plotted using the age ranges for each taxon; branch lengths do not reflect the number of character state changes. Because species within the same genus had the same codings, only genera are listed in the phylogeny. Values on branches represent bootstrap support; branches without values had less than 50% support. Tree statistics: shortest tree length = 188, Consistency Index = 0.70, Retention Index = 0.65, Rescaled Consistency Index = 0.45. Age ranges are approximate, and are based on those in Gates et al. [3], aside from Brachylophosaurini, which have been recalibrated as in Fig 1. Note that the age scale changes before and after 80 Ma. Also note that the age of *Bactrosaurus* has been variably proposed to be any time from late Turonian to early Maastrichtian in age [49–51].

When *Probrachylophosaurus bergei* is added to Gates et al.’s [3] matrix, with extraneous taxa removed (see Methods), but all characters included (S3 File), two most parsimonious trees were produced. The strict consensus tree recovers *Probrachylophosaurus* and *Brachylophosaurus* as sister taxa, with *Acristavus* as the basalmost member of Brachylophosaurini (Fig 23). *Probrachylophosaurus*, *Brachylophosaurus*, and *Maiasaura*, but not *Acristavus*, share two apomorphic character states: character 36 state 1, presence of solid nasal crest; and character 37 state 1, circumnarial fossa terminates anterior to nasal crest. *Acristavus*, *Probrachylophosaurus*, and *Maiasaura*, but not *Brachylophosaurus*, share one apomorphic character state: character 53 state 0, straight to slightly curved ventral margin of the rostral process of the jugal. *Probrachylophosaurus* and *Brachylophosaurus* are united as sister taxa due to one apomorphic character state: character 105 state 0, postacetabular process is less than 40 percent the total length of the ilium. *Probrachylophosaurus* differs from *Acristavus*, *Brachylophosaurus*, and *Maiasaura* in its state for one character: character 72 state 0, low basipterygoid transverse ridge of the basisphenoid. The bootstrap 50% majority-rule consensus tree (50,000 replicates) finds 50% support for *Probrachylophosaurus* and *Brachylophosaurus* as sister taxa, with the positions of *Acristavus* and *Maiasaura* having less than 50% support.

### Phylogenetic Relationships

Brachylophosaurini is supported as a robust clade in all recent phylogenetic analyses, but the relationships amongst its members vary. Prior to the description of *Acristavus gagslarsoni*, this clade consisted solely of *Maiasaura peeblesorum* and *Brachylophosaurus canadensis* [1, 28, 43]. Prieto-Márquez [29] recovered *Maiasaura* and *Brachylophosaurus* as sister taxa, with *Acristavus* (“Two Medicine OTU”) as the basal member of the clade. However, Gates et al. [3] recovered *Acristavus* and *Maiasaura* as sister taxa.

When *Probrachylophosaurus* is added to the Prieto-Márquez [29] and Gates et al. [3] matrices, it is recovered with confidence as a member of Brachylophosaurini (bootstrap support 87% in both analyses). *Acristavus* is recovered as the basal taxon in all analyses, consistent with its stratigraphic position; thus, *Acristavus* or a close relative was likely ancestral to the rest of Brachylophosaurini. The inclusion of *Probrachylophosaurus* in the Prieto-Márquez [29] matrix still results in *Maiasaura* and *Brachylophosaurus* as sister taxa, but with much weaker bootstrap support for the clade (56% vs. 69% in [29]). *Probrachylophosaurus* is recovered as intermediate between *Acristavus* and the *Brachylophosaurus*-*Maiasaura* clade. The inclusion of *Probrachylophosaurus* in the Gates et al. [3] matrix yields *Probrachylophosaurus* and *Brachylophosaurus* as sister taxa, with *Maiasaura* intermediate between them and *Acristavus*. The differing positions of *Maiasaura* relative to *Probrachylophosaurus* and *Brachylophosaurus* in each analysis leads to uncertainty regarding the timing of the cladogenic split between the *Brachylophosaurus* and *Maiasaura* lineages. The analysis based on the Gates et al. [3] matrix suggests that *Maiasaura* diverged from the *Probrachylophosaurus*-*Brachylophosaurus* lineage sometime between the stratigraphic existence of *Acristavus* and *Probrachylophosaurus* (Fig 23). The analysis based on the Prieto-Márquez [29] matrix suggests that the *Maiasaura* lineage either diverged from the *Brachylophosaurus* lineage sometime between the stratigraphic existence of *Probrachylophosaurus* and *Brachylophosaurus*, or *Maiasaura* is derived from *Brachylophosaurus* (Fig 22).

In the analysis based on the Prieto-Márquez [29] matrix, *Brachylophosaurus* and *Maiasaura* are united by two characters: 44 (dt13) caudodorsal point on the coronoid process of the dentary and 106 (j4) position of the caudoventral apex of the rostral process of the jugal relative to the caudodorsal margin of the rostral process. The caudodorsal point on the coronoid process of the dentary is potentially very useful for taxon identification, but is also problematic. The caudodorsal margins of Brachylophosaurini coronoid processes are very thin, and are broken in many specimens. The caudodorsal margins of the coronoid processes of *Acristavus* and *Probrachylophosaurus* are extremely thin and partially broken; they do not preserve points, and the thinness of the bone seems to indicate that there could not have been a point, but this cannot be confirmed with absolute confidence. Also, the caudodorsal point enlarges ontogenetically in *Brachylophosaurus* and *Maiasaura*; small individuals lack the point entirely. Thus, the maturity of a specimen lacking the caudodorsal point must be considered before including it in phylogenetic analyses. The *Acristavus* and *Probrachylophosaurus* holotypes are from large enough individuals that they should display the adult condition for this character. Character 106 (j4) concerns the relative positions of the caudodorsal and caudoventral points of the rostral process of the jugal. *Maiasaura* is coded as having the same state as *Brachylophosaurus*; the caudoventral point is directly ventral to the caudodorsal point, rather than being posterior to the caudodorsal point as it is in *Acristavus* and *Probrachylophosaurus*. However, in *Maiasaura*, the caudoventral point is slightly posterior to the caudodorsal point, and so is actually intermediate between the states of *Acristavus*-*Probrachylophosaurus* and *Brachylophosaurus*. In summary, the phylogenetic analyses concur in placing *Acristavus* as the basal-most member of the Brachylophosaurini, consistent with its relative stratigraphic position, but the relationships among *Probrachylophosaurus*, *Brachylophosaurus*, and *Maiasaura* vary depending on the cladistic matrix used.

## Histology

**Fig 24:**
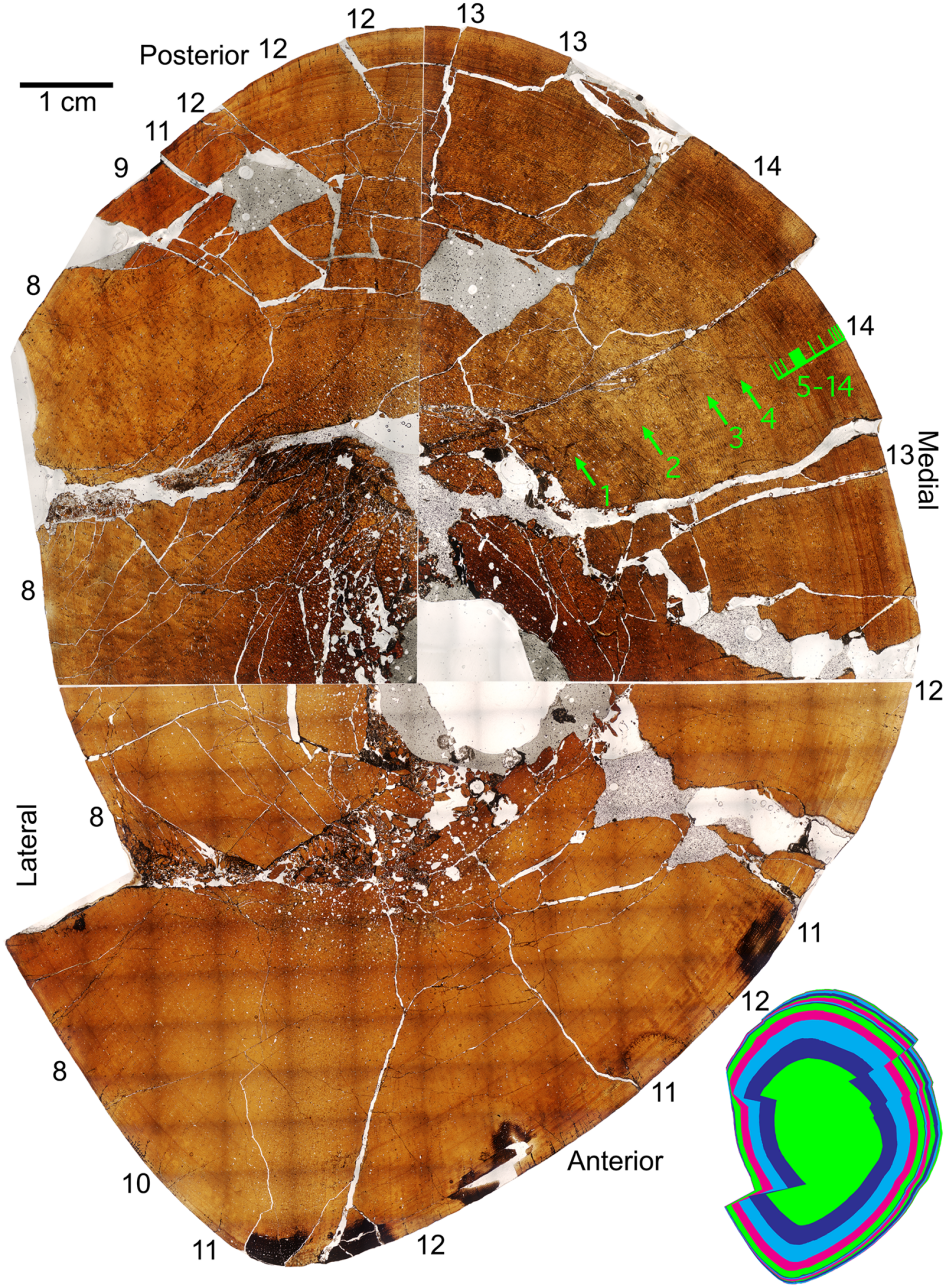
Tibial histology of MOR 2919, mid-diaphyseal cross-section. Numbered green arrows identify lines of arrested growth (LAGs). The closely spaced outer LAGs are indicated with tick marks along a green line. Numbers around the circumference of the cross-section indicate the number of LAGs preserved in that radial segment of the tibia. Note that diagenetic crushing has displaced several segments radially inward. Thumbnail image in lower right corner colors the area between each pair of LAGs to highlight locations of radial displacement as well as to indicate bone deposition between later LAGs occurring primarily along the posteromedial region. A high-resolution version of this figure is available at http://www.morphobank.org/index.php/Projects/Media/id/377387/project_id/2157.

**Fig 25:**
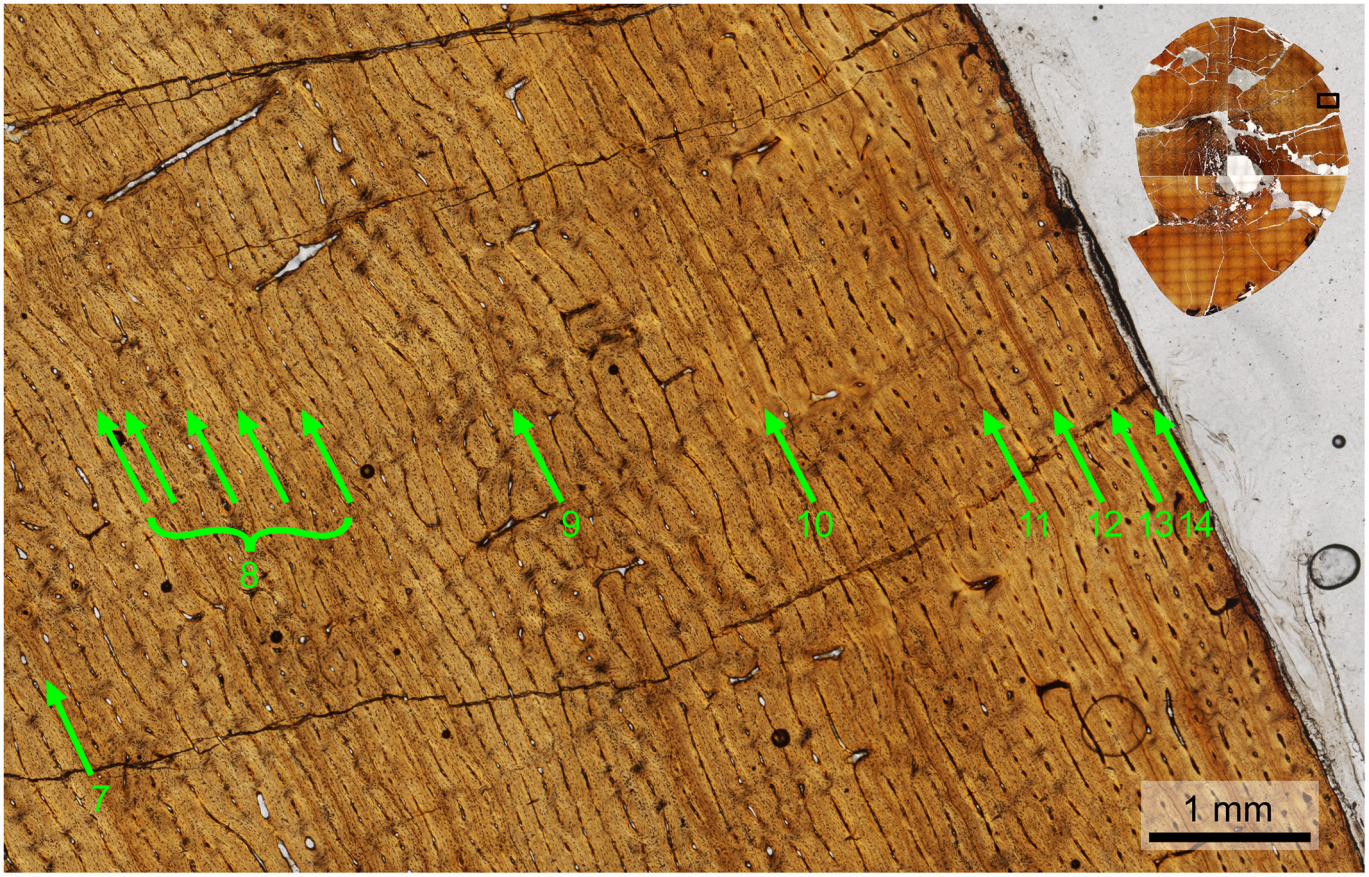
Outer cortical histology of MOR 2919 tibia. This is a higher-resolution image of the posteromedial cortex pictured in Fig 24, as indicated by the black box on the upper right thumbnail image of the entire mid-diaphyseal cross-section. Numbered green arrows identify lines of arrested growth (LAGs), including the multiple lines of LAG zone 8 (see text). A high-resolution version of this figure is available at http://www.morphobank.org/index.php/Projects/Media/id/377388/project_id/2157.

The left tibia of MOR 2919 includes 14 lines of arrested growth (LAGs) (Figs 24 and 25). Because LAGs represent annual interruptions in bone growth [52], this indicates that MOR 2919 was 14 years old at time of death. The outermost LAGS (11–14) are closely spaced, yet still retain vascularity, and so cannot be considered an External Fundamental System (EFS). This indicates that MOR 2919 was still increasing its tibia circumference, and therefore presumably still increasing it body length and body mass, at time of death.

Bone apposition rates in hadrosaurids are generally greater on the posteromedial side of the cortex than on the anterolateral cortex (H. N. Woodward, personal communication 2013). In MOR 2919, this produced thicker zones between early LAGs on the posteromedial side. Later, bone deposition continued on the posteromedial side while it had ceased on the lateral side, resulting in the highest number of LAGs (14) countable on the posteromedial side, with the number of LAGs decreasing along the posterolateral (12–8 LAGs) and anteromedial (13–11 LAGs) cortex, and the fewest number of LAGs (8) countable on the lateral cortex (Fig 24). LAGs 1–12 are well defined and visible at 10x total magnification. When present, LAGs 13 and 14 are visible at 40x total magnification. LAG 13 is visible as a darkened band that sometimes contains one or more lines. Thus, LAG 13 may not actually be a LAG, but may instead be an annulus, which represents an annual marker of growth that slowed but did not actually stop as it would in a LAG. LAG 14, when present, is extremely close to the outer surface of the cortex.

**Fig 26:**
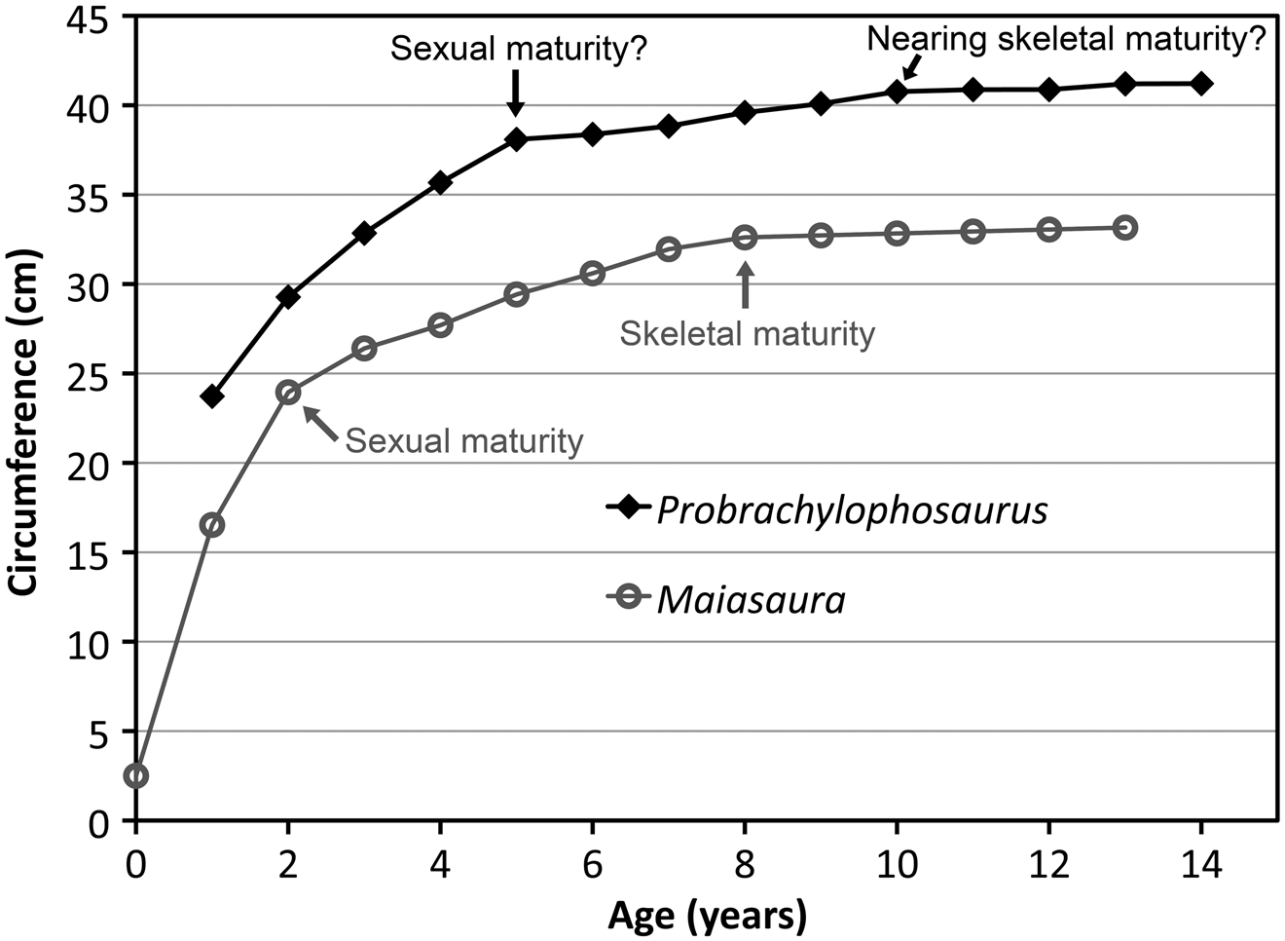
Growth curves of tibial cortical circumference at each line of arrested growth in *Probrachylophosaurus bergei* gen. et sp. nov. (MOR 2919) and *Maiasaura peeblesorum* (MOR 758 NC-7-4-96-16 “T46”). Age in years was determined by counting the number of lines of arrested growth within the tibial cortex. MOR 2919 left tibia is 119 cm long. *Maiasaura* data are taken from the largest tibia with complete histologic data (93 cm long) from Woodward et al. [53]. Inflection points in the growth of *Probrachylophosaurus* occur at 5 years and 10 years; these inflection points occur earlier in *Maiasaura*, at 2–3 years and 8 years. In *Maiasaura*, the first inflection point is interpreted as sexual maturity, and the second inflection point occurs at the initiation of the external fundamental system (EFS), representing skeletal maturity [53]. Although MOR 2919 has an apparent second inflection point, it does not possess an EFS, so it is not skeletally mature.

Generally, the zone thickness (spacing between LAGs) decreased each year, indicating a faster growth rate in the first few years of life, followed by a gradual decrease in growth rate (Fig 26). However, LAGs 5, 6, and 7 are relatively closely spaced, with wider growth zones occurring between later LAGs. This may indicate that the two growing seasons in between LAGs 5 and 6, and 6 and 7, were stressful times of low access to resources, and so growth was limited.

LAG 8 is actually a zone of one to five closely spaced lines occurring after a zone of typical annual bone deposition (Fig 25). Similar to the locations of outermost LAG deposition, LAG zone 8 contains the most lines (five) on the posteromedial side of the tibia, decreasing incrementally around the circumference, with a single line along the anterolateral to posterolateral sides. This LAG zone is interpreted as the result of a mild “unfavorable season” (*sensu* Köhler et al., 2012), perhaps a mild winter, during which small increments of bone deposition occurred between multiple pauses in growth, rather than the typical single cessation of growth for the entire unfavorable season. The circumference of LAG 8 was measured at the outermost line of the zone, because the outer circumference of this line marks the resumption of growth in the favorable season, as it does for all other LAGs.

**Fig 27:**
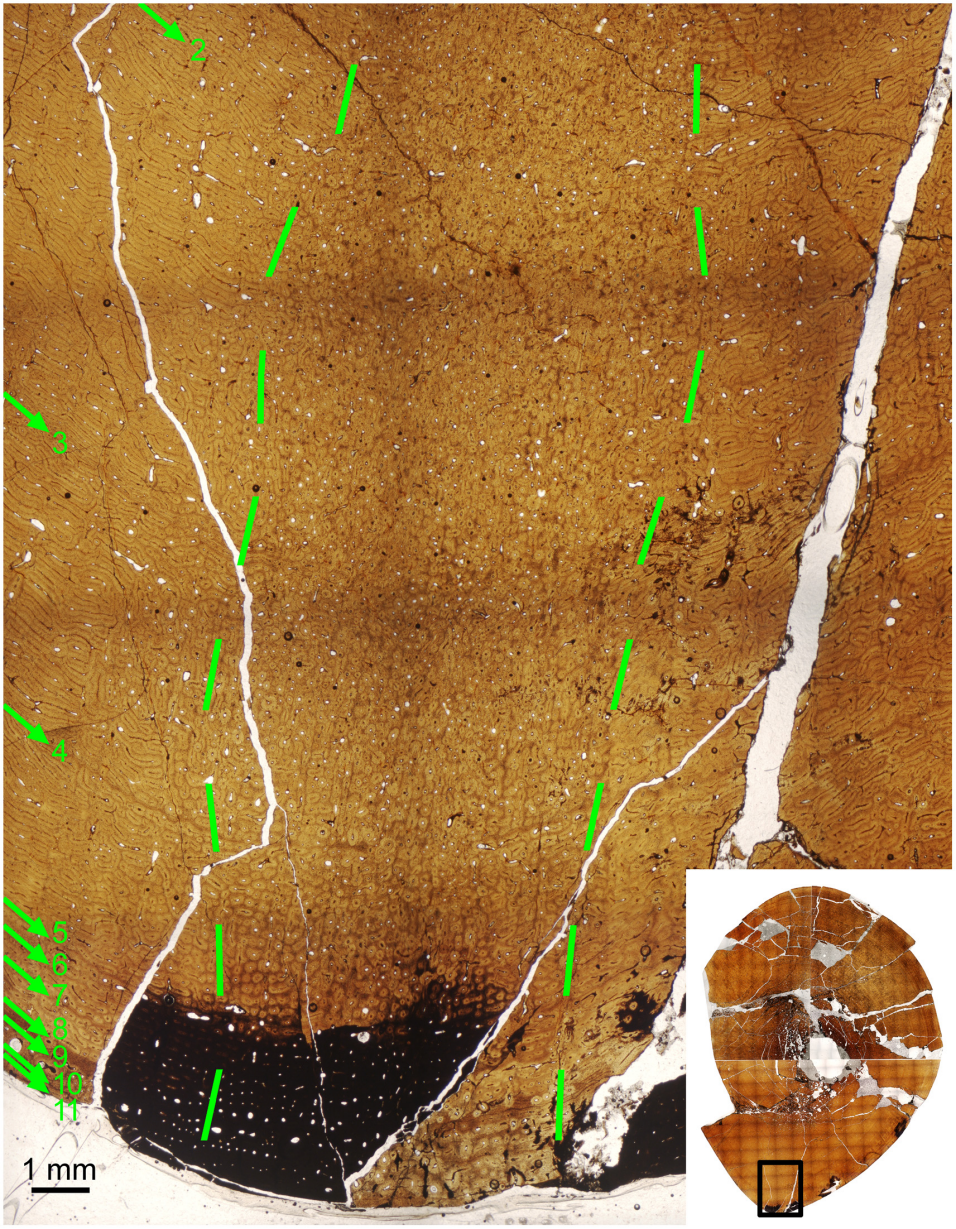
Remodeled “fountain” of secondary osteons on anterolateral side of cortex, MOR 2919 tibia. Margins of the remodeled region are indicated with dashed green lines. Numbered green arrows identify lines of arrested growth. Dark areas near edge of cortex are sections of bone that began to separate from the slide, requiring additional adhesive. Black box on the thumbnail image on lower right indicates location of Fig 27 within the entire tibial cross-section. A high-resolution version of this figure is available at http://www.morphobank.org/index.php/Projects/Media/id/377389/project_id/2157.

The bone is minimally remodeled, such that LAG 1 is clearly visible along most of its circumference, and still faintly visible on the anterior side where secondary osteons mostly obscure the original bone texture. Secondary osteons are mainly confined to the area between the medullary cavity and LAG 1. Isolated secondary osteons are also scattered sparsely throughout the cortex. The only area of intensive remodeling outside of LAG 1 occurs as a narrow “fountain” of secondary osteons leading from the medullary cavity to the anterolateral outer cortex (Fig 27). This location corresponds to the anterolateral border of the tibia, along which runs the M. tibialis anterior. Dilkes [54, 55] notes that in turtles, *Sphenodon*, and squamates, the M. tibialis anterior originates on the anterior, medial, and lateral sides of the diaphysis of the tibia, whereas in crocodilians it originates from the proximolateral surface of the tibia, and in birds it originates on the lateral outer cnemial crest of the tibia and the anterior external condyle of the femur. Dilkes [54, 55] reconstructed the M. tibialis anterior of *Maiasaura* as originating on the cnemial crest of the proximal tibia due to the presence of muscle scarring on the cnemial crest. If the origin of the M. tibialis anterior actually covered a larger area, continuing down the anterolateral tibial diaphysis, then the “fountain” of secondary osteons along the anterolateral border of MOR 2919 may be due to it being a muscle attachment area; however, the cortical surface is smooth, with no scarring indicative of muscle attachment. Alternatively, the anterolateral border of the tibia may simply be an area subjected to great compressive or extensive stress, triggering Haversian remodeling.

This remodeled region is possibly similar to the “anterolateral plug” in a *Dysalotosaurus* (Ornithischia: Dryosauridae) tibia [56], but the “fountain” in MOR 2919 is a continuous band of fairly consistent width connecting the medullary cavity to the periosteal surface and is nearly entirely composed of secondary osteons; the “plug” in *Dysalotosaurus* is “whirl-like” and may not extend to the medullary cavity or the periosteal surface. The “plug” is initially composed of randomly oriented primary osteons, and later contains a higher density of secondary osteons than are found in other areas of the tibial cross-section; it is interpreted as forming due to this area being a muscle attachment site [56]. The “fountain” of MOR 2919 and “anterolateral plug” of *Dysalotosaurus* are similar in their location and presence of secondary osteons, but the radial extent and degree of remodeling are much greater in MOR 2919. Adult *Maiasaura* also have concentrations of secondary osteons on the anterior side of the tibia that remodel the radial vascularity of the primary bone tissue in this area; Woodward et al. [53] hypothesize that this remodeling results from muscle insertion on the anterior tibial border.

### Histologic Maturity

The growth inflection points (Fig 26) occur later in MOR 2919 than in *Maiasaura*, indicating later sexual and skeletal maturation. Whereas *Maiasaura* is hypothesized to reach sexual maturity between two and three years of age [53], the corresponding inflection point in the growth of MOR 2919 occurs at 5 years of age. This suggests that *Probrachylophosaurus* had delayed sexual maturity relative to *Maiasaura*.

At the second inflection point, *Maiasaura* begins depositing an external fundamental system (EFS), indicating skeletal maturity [53]. MOR 2919 decreases its growth rate at the second inflection point, but does not deposit an EFS, so it has not yet reached true skeletal maturity. This second decrease in growth rate occurs later in MOR 2919 (10 years) than in the largest sampled *Maiasaura* tibia (8 years). The later achievement of the second inflection point, as well as the lack of an EFS, suggests that *Probrachylophosaurus* attained skeletal maturity later than *Maiasaura*. However, the age of skeletal maturity in *Maiasaura* is individually variable (Woodward et al. in review), so a greater sample size of *Probrachylophosaurus* is needed to determine whether the apparent delayed maturation relative to *Maiasaura* is real or simply reflects individual variation in growth rates. In either case, MOR 2919’s histology suggests that *Probrachylophosaurus* is a larger-bodied taxon than *Maiasaura* at all growth stages.

Although MOR 2919 lacks an EFS, its histology confirms that its growth rate had greatly slowed and it was possibly somewhat near skeletal maturity. Thus, its “adult” status is supported by three independent lines of evidence: histology, large body size, and externally fused and remodeled axial sutures. The term “adult”, as applied to MOR 2919, is used to indicate that this is not a subadult specimen that may reasonably be expected to grow a full paddle-shaped nasal crest as seen in *Brachylophosaurus canadensis*. Its skull and body size, compared to the size distribution of its close relative *B*. *canadensis*, clearly categorize it as an adult *sensu* Evans [26]. However, because MOR 2919 has not attained an EFS, it is technically not skeletally mature, and by some definitions (e.g., [57]) would not be considered an adult. In this paper, MOR 2919 is considered an adult, and a fully skeletally mature specimen with an EFS would be considered a “senescent adult”.

**Fig 28:**
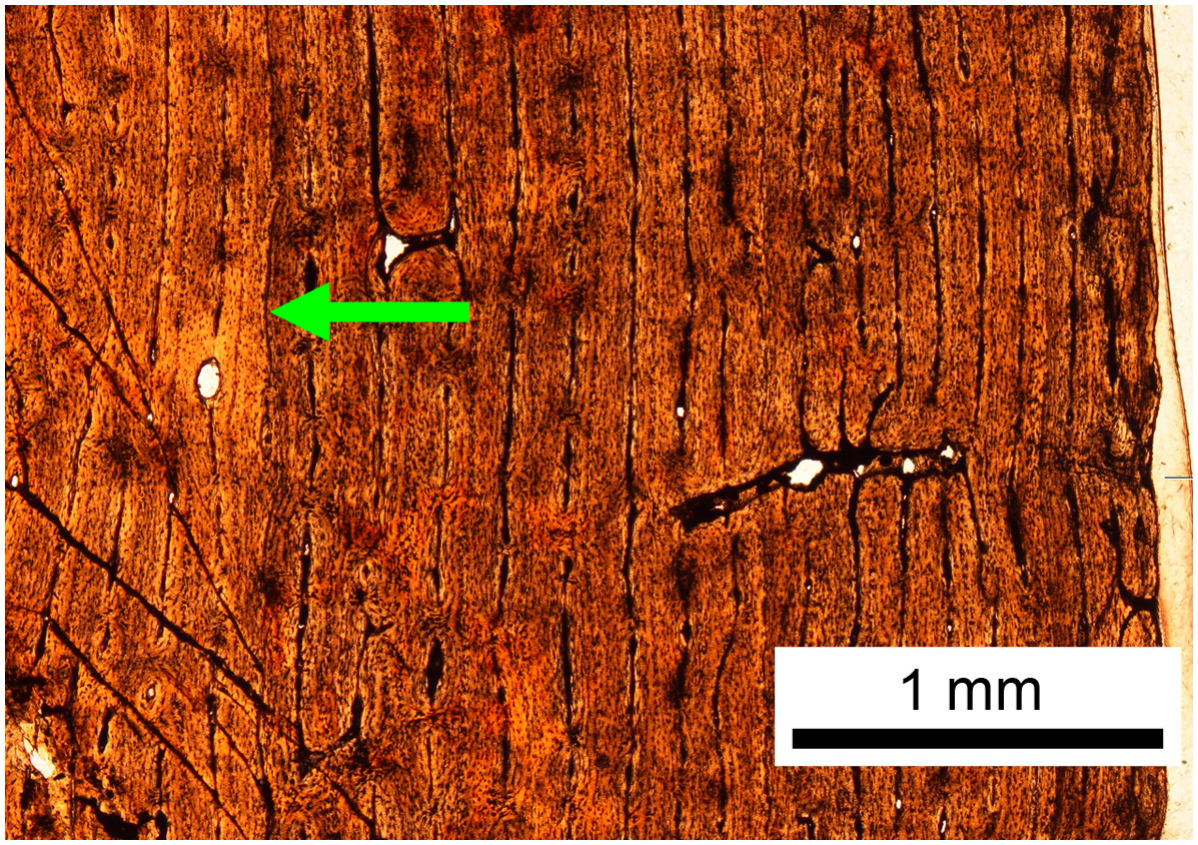
Outer cortical histology of MOR 794 *Brachylophosaurus canadensis* femur. The right femur (length 129.5 cm) of MOR 794 was histologically sampled using a core of the outer cortex of the anteromedial mid-diaphysis rather than a complete cross-section of the mid-diaphysis, so the complete LAG record was not sampled and the individual’s age in years is unknown. Green arrow indicates the outermost LAG. Cortical surface is on the right.

MOR 794, a large “adult” *Brachylophosaurus canadensis*, is similar to MOR 2919 in that it also has not yet deposited an EFS (Fig 28). The LAGs in the outermost cortex of the femur of MOR 794 are more widely spaced than those of MOR 2919; the outermost 3 mm of the femur of MOR 794 contain only one LAG, whereas the outermost 3 mm of the tibia of MOR 2919 contain five LAGs (Fig 25). Thus, MOR 2919, with its smaller nasal crest, is at a slightly later ontogenetic stage than MOR 794, and so MOR 2919 cannot represent an immature stage of *Brachylophosaurus canadensis*.

## Discussion

Ontogeny is intimately tied to evolution, as in a very general sense, ontogeny recapitulates phylogeny [58, 59]. This is often expressed in juvenile dinosaurs, which may bear morphologic traits of their adult ancestors (peramorphy) that are not expressed in the adults of the later taxon, e.g. juvenile *Bactrosaurus* dentition resembling that of iguanodonts [60]. Because juveniles may retain ancestral characteristics, without proper stratigraphic data they may be mistaken as older, more basal taxa. Indeed, when juvenile specimens are coded into a phylogenetic matrix, they are placed more basally on the resulting cladogram than are adults of that taxon [61, 62].

Within hadrosaurine and lambeosaurine skulls, the most drastic ontogenetic changes occur in the morphology of the premaxillae, nasals, and supraoccipitals [63]. As the individual grows, its premaxillae and nasals generally enlarge and change shape to form the crest [63], with the crest typically moving posteriorly during ontogeny [38, 64]. Bones far from the incipient crest (quadrates, dentaries, and maxillae) change the least [63]. The number of tooth rows increases [37, 38], articular surfaces become more rugose, and the relative size of the orbit decreases [37]. These general trends are observed in both *Probrachylophosaurus* and *Brachylophosaurus*.

The nasal crest of *Brachylophosaurus canadensis* enlarges late in ontogeny. Prieto-Márquez [1] separated *Brachylophosaurus* specimens into “slender” and “robust” morphotypes, and stated that these individuals were approximately the same size. Although specimens of the two morphotypes have roughly the same interorbital and postorbital widths [1, 23], these widths have been affected by diagenetic compression. The robust specimen MOR 794 is laterally compressed, decreasing its cranial width; the slender specimens MOR 1071-7-7-98-86 and MOR 1071-7-16-98-248 are dorsoventrally compressed, increasing their cranial widths. Thus, although their apparent sizes are similar, after accounting for differential compression slender *Brachylophosaurus* specimens are slightly smaller than robust specimens. Slender and robust *Brachylophosaurus* specimens are both found in nearly the same horizons at Malta, Montana, and are therefore most parsimoniously ontogenetic stages of the same species. Cuthbertson and Holmes [23] noted that the slender and robust morphotypes are not discrete; *Brachylophosaurus* specimens such as FMNH PR 862 and TMP 1990.104.001 possess crests of intermediate sizes. The presence of intermediate crest morphologies supports individual or ontogenetic variation. Also, individual variation in body size could account for the presence of specimens with different crest sizes at similar body sizes.

Like many dinosaur taxa, hadrosaurids reached nearly their full adult size relatively quickly, and then grew increasingly slowly for the rest of their lives [65]. In modern animals such as cassowaries, the cranial crest grows rapidly late in ontogeny, often after the rate of skeletal growth has greatly slowed and the individual is nearly at full adult size [66]. A near-adult cassowary with a skull length of 165 mm possesses a small incipient crest, and after a mere 5 mm (3%) of additional growth in skull length, acquires a full adult casque that covers most of the dorsal skull. *Pachycephalosaurus* and *Stegoceras* juveniles have flat skulls, gaining their distinctive frontoparietal domes later in ontogeny [67, 68]. Centrosaurine ceratopsids also developed their pronounced cranial ornamentation late in ontogeny [69]. The nasal crests of slender and robust *Brachylophosaurus* follow this pattern of rapidly increasing crest size with a very small increase in overall skull size.

In *Brachylophosaurus*, the nasal crest elongates and flattens posteriorly ontogenetically, and the posterior margin of the nasofrontal suture migrates posteriorly as well. In MOR 2919, the nasofrontal suture is anteriorly placed as in a subadult *Brachylophosaurus*, and the nasal crest is shorter than that of any adult *Brachylophosaurus*. Yet, MOR 2919 is not a subadult; in addition to its large size, the braincase elements and vertebral neural arches are completely fused externally, with most sutures obliterated, and histology confirms that MOR 2919 was nearing skeletal maturity. In slender adult *Brachylophosaurus* (MOR 1071), cranial sutures are still clearly defined, and sutures are still visible in the robust morphology of adult *Brachylophosaurus* (MOR 794). Although all sutures of braincase elements of MOR 2919 are fused and nearly obliterated, the nasofrontal suture and internasal suture are disarticulated and show no indications of fusion. The nasofrontal suture in *Brachylophosaurus* becomes more rugose ontogenetically, stabilizing the suture as the nasal crest grows. The lack of nasofrontal fusion in MOR 2919 suggests that the nasal crest was still enlarging at time of death.

The holotype of *Brachylophosaurus goodwini* (UCMP 130139) and MOR 2919 were both collected from the Kennedy Coulee area, but the *Brachylophosaurus goodwini* holotype was located stratigraphically lower in the coulee. Unfortunately, UCMP 130139 does not preserve the posterior nasals, which are a critical character in defining *Probrachylophosaurus bergei*. UCMP 130139 is adult-sized, and its frontals exhibit an anteriorly-located nasofrontal suture that does not cover the entire frontals, similar to that of MOR 2919 and subadult *Brachylophosaurus canadensis*. This supports UCMP 130139 and MOR 2919 being more closely related than either is to *B*. *canadensis*. However, UCMP 130139 exhibits anomalously deep frontal depressions that preclude it from being referred to *P*. *bergei*. If the deep frontal depressions are not pathologic, taphonomic, diagenetic, or individual variation, then they may potentially be used as an autapomorphy to resurrect the species “*B*.” *goodwini*, although it will need a different genus name. Given the poor preservational condition of the frontals of UCMP 130139, the specimen should be referred to Brachylophosaurini indet. pending additional specimens with similar frontal depressions.

**Fig 29:**
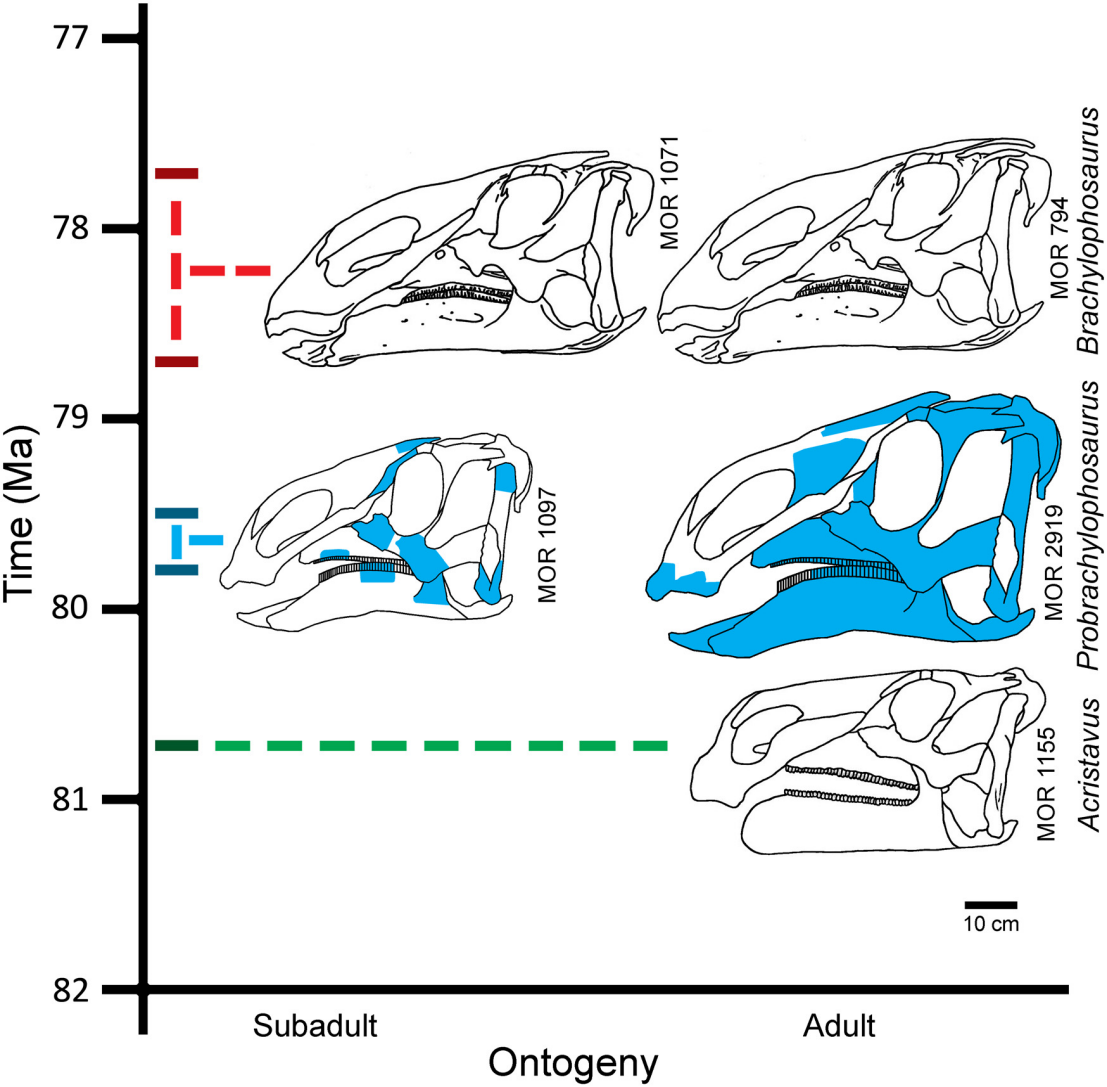
Ontogenetic and anagenetic hypothesis of brachylophosaurin evolution. *Probrachylophosaurus bergei* gen. et sp. nov. is proposed as an intermediate member of the lineage leading from *Acristavus gagslarsoni* to *Brachylophosaurus canadensis*. Shaded blue areas indicate known elements of *Probrachylophosaurus*. Skull outlines of *Acristavus* and *Brachylophosaurus* are used courtesy of Terry A. Gates. The reconstruction of the MOR 1071 *Brachylophosaurus* skull is a composite of an articulated skull roof with a scaled-down copy of the MOR 794 skull outline. All skulls are scaled to the same 10 cm scale bar. The horizontal axis is not to scale; the MOR 1071 reconstruction is much closer to MOR 794 in size and hypothesized maturity than MOR 1097 is to MOR 2919. Radiometric ages have been recalibrated to the Fish Canyon sanidine standard (28.305 +/- 0.036 Ma) of Renne et al. [10] from the originally published values [3, 11–13]; see text and Table 1 for further recalibration details. The age of the *Acristavus* holotype was precisely estimated by Gates et al. [3]. The age of the Comrey Sandstone Zone of the Oldman Formation is not tightly constrained, leading to uncertainty in the exact age of *Brachylophosaurus*.

The morphologies of elements of *Probrachylophosaurus* are consistently more similar to members of Brachylophosaurini than to other hadrosaurids. Within Brachylophosaurini, elements of *Probrachylophosaurus* are either most similar to *Brachylophosaurus* or *Acristavus*, or intermediate between these taxa, as predicted by their relative stratigraphic positions. This supports the hypothesis of *Probrachylophosaurus bergei* representing an intermediate taxon within the *Acristavus*-*Brachylophosaurus* lineage (Fig 29).

In some morphologic characters within Brachylophosaurini, the subadult morphology of the stratigraphically younger taxon matches the adult condition of the stratigraphically older taxon, illustrating a peramorphic trend of immature animals of a descendant taxon resembling adults of the ancestral taxon [60]. The palatine process of the jugal in subadult *Probrachylophosaurus* resembles the condition of adult *Acristavus*. The nasal crest in *Brachylophosaurus* increases in size late in ontogeny: subadults lack any crest, similar to the adult form of their proposed ancestor *Acristavus*; small adults have a small crest reminiscent of their proposed ancestor *Probrachylophosaurus*; and large adults have a large broad paddle-shaped crest. The nasofrontal suture of small subadult *Brachylophosaurus* is similar to that of adult *Probrachylophosaurus*, although *Probrachylophosaurus* has the deep rugosities of a more mature suture. The prequadratic process of the squamosal of small subadult *Brachylophosaurus* is also more similar to that of adult *Probrachylophosaurus* than to adult *Brachylophosaurus*.

*Probrachylophosaurus bergei* shares many morphologic similarities with *Brachylophosaurus canadensis*, but in other characters is more similar to *Acristavus gagslarsoni*. Thus, placing *P*. *bergei* as merely a new species of either genus would be problematic. Depending on the cladistic matrix used, *P*. *bergei* may be the sister taxon to *B*. *canadensis* and could be classified as a member of the same genus (Fig 23), or *Maiasaura peeblesorum* may be the sister taxon to *B*. *canadensis* (Fig 22), requiring *P*. *bergei* to be classified as a separate genus. Given this phylogenetic uncertainty, *Probrachylophosaurus bergei* should be considered a unique genus. Future discoveries of additional intermediate specimens will hopefully clarify evolutionary relationships within the Brachylophosaurini clade.

## Conclusions

In the early years of dinosaur paleontology, specimens were collected as isolated points, each so morphologically unique that their evolutionary relationships were difficult to determine. As more fossil specimens are collected, the gaps in morphology that previously separated species are being filled. With larger sample sizes resulting in more continuous series of fossils with good stratigraphic resolution, variations in morphology can be analyzed in a more complete context, and attributed to ontogeny, evolution, taphonomic alteration, biogeography, or individual variation [70]. A better understanding of stratigraphy and advancements in radiometric dating enable precise temporal correlation of geographically separated localities. Taxa can then be placed in temporal sequence, allowing tests of evolutionary hypotheses.

A precise stratigraphic framework is critical for determining whether morphological variations of adult specimens are due to evolution or are variations within a roughly contemporaneous population. If closely related taxa do not overlap stratigraphically, the pattern is more parsimonious with anagenesis than cladogenesis. Recent research has greatly increased the sample size and stratigraphic resolution of specimens from the Judith River and Hell Creek Formations of Montana and their Canadian equivalents, revealing several potential anagenetic lineages in Campanian and Maastrichtian ornithischians [7, 45, 71–73].

Because *Probrachylophosaurus bergei* is stratigraphically older than all *Brachylophosaurus canadensis* specimens, it is hypothesized to represent a basal brachylophosaur morphology, early in the evolution of this lineage from a non-crested ancestor. Thus, the small crest of *Probrachylophosaurus* would represent a transitional nasal morphology between a non-crested ancestor such as *Acristavus* and the larger crests of adult *Brachylophosaurus*. The fourth member of Brachylophosaurini, *Maiasaura*, would represent a cladogenic event, diverging from the lineage that led to *Brachylophosaurus* at a currently unknown point.

## Supporting Information

S1 Fig Ray teeth from Oldman Formation equivalent localities in the Judith River Formation of Montana. Upper half of figure (A-D): two *Myledaphus bipartitus* teeth in occlusal view (A, C) and side view (B, D) with worn occlusal surface of the crowns from the MOR 1071 (JR-224) *Brachylophosaurus canadensis* bonebed in Malta, Montana, at a horizon equivalent to Unit 2 (Comrey Sandstone Zone) of the Oldman Formation. Lower half of figure (E-J): three teeth tentatively attributed to *Pseudomyledaphus* sp. from the upper muddy zone of Kennedy Coulee, Montana, equivalent to Unit 1 of the Oldman Formation. (E-F) tooth with unworn occlusal surface of crown; (G-H) tooth with worn occlusal surface of crown; (I-J) tooth with extremely worn occlusal surface of crown. Dashed lines indicate partially broken crown.
(TIF)

S2 Fig Phylogeny of hadrosaurines based on Prieto-Márquez [29]. Strict consensus of two most parsimonious trees resulting from adding *Probrachylophosaurus bergei* to Prieto-Márquez’s [29] matrix with minor character recodings as discussed in text, but no excluded characters. Values on branches represent bootstrap support; branches without values had less than 50% support. Tree statistics: shortest tree length = 627, Consistency Index = 0.72, Retention Index = 0.68, Rescaled Consistency Index = 0.49. The clade of *Probrachylophosaurus*, *Brachylophosaurus*, and *Maiasaura* with the exclusion of *Acristavus* had 55% bootstrap support.
(TIF)

S1 File Character matrix based on Prieto-Márquez [29] with all characters included, in nexus format. Change file extension from.txt. to.nex for use in PAUP.
(TXT)

S2 File Character matrix based on Prieto-Márquez [29] with some characters excluded, in nexus format. Change file extension from.txt. to.nex for use in PAUP.
(TXT)

S3 File Character matrix based on Gates et al. [3], in nexus format. Change file extension from.txt. to.nex for use in PAUP.
(TXT)

S1 Text Ray teeth identification. (PDF)
