# Supplementary figures and images for: A New Brachylophosaurin Hadrosaur (Dinosauria: Ornithischia) with an Intermediate Nasal Crest from the Campanian Judith River Formation of Northcentral Montana
Source: PLoS One. 2015 Nov 11;10(11):e0141304. doi: 10.1371/journal.pone.0141304 (PMC4641681; doi:10.1371/journal.pone.0141304)

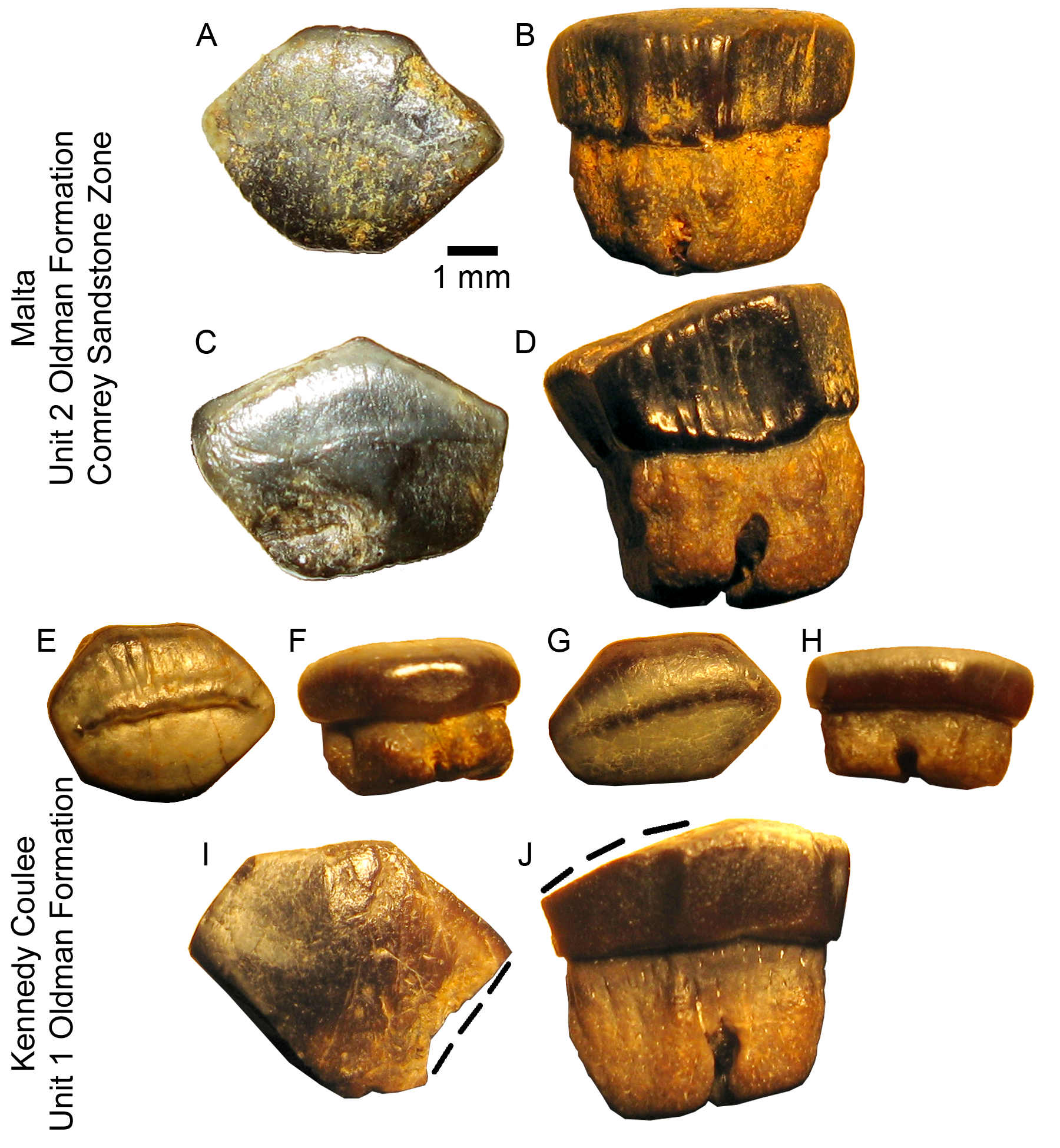

Supplement: S1 Fig — Upper half of figure (A-D): two Myledaphus bipartitus teeth in occlusal view (A, C) and side view (B, D) with worn occlusal surface of the crowns from the MOR 1071 (JR-224) Brachylophosaurus canadensis bonebed in Malta, Montana, at a horizon equivalent to Unit 2 (Comrey Sandstone Zone) of the Oldman Formation. Lower half of figure (E-J): three teeth tentatively attributed to Pseudomyledaphus sp. from the upper muddy zone of Kennedy Coulee, Montana, equivalent to Unit 1 of the Oldman Formation. (E-F) tooth with unworn occlusal surface of crown; (G-H) tooth with worn occlusal surface of crown; (I-J) tooth with extremely worn occlusal surface of crown. Dashed lines indicate partially broken crown. (TIF) [file pone.0141304.s001.tif]

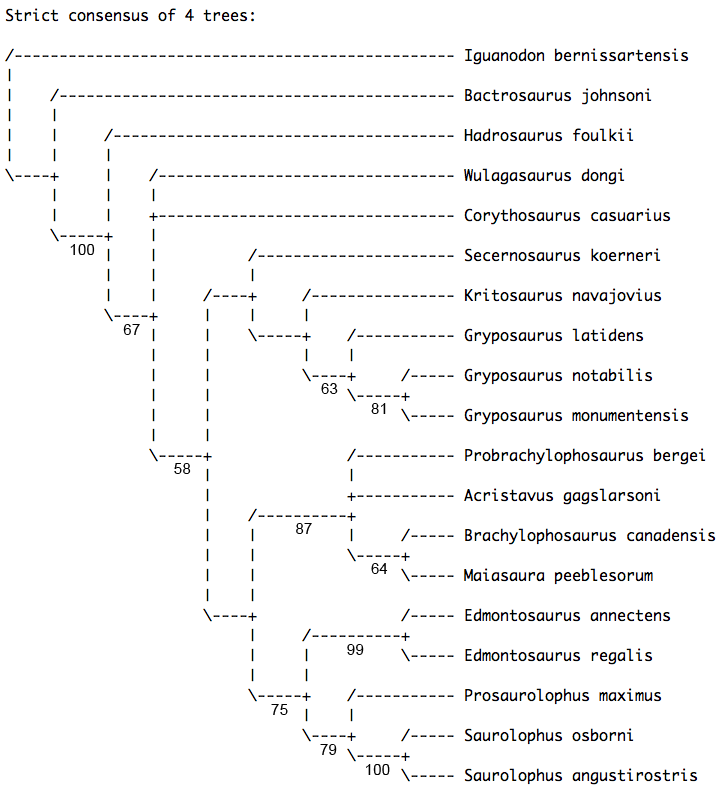

Supplement: S2 Fig — Strict consensus of two most parsimonious trees resulting from adding Probrachylophosaurus bergei to Prieto-Márquez’s [29] matrix with minor character recodings as discussed in text, but no excluded characters. Values on branches represent bootstrap support; branches without values had less than 50% support. Tree statistics: shortest tree length = 627, Consistency Index = 0.72, Retention Index = 0.68, Rescaled Consistency Index = 0.49. The clade of Probrachylophosaurus, Brachylophosaurus, and Maiasaura with the exclusion of Acristavus had 55% bootstrap support. (TIF) [file pone.0141304.s002.tif]
