# Supplementary material for: A New Brachylophosaurin Hadrosaur (Dinosauria: Ornithischia) with an Intermediate Nasal Crest from the Campanian Judith River Formation of Northcentral Montana
Source: PLoS One. 2015 Nov 11;10(11):e0141304. doi: 10.1371/journal.pone.0141304 (PMC4641681; doi:10.1371/journal.pone.0141304)
Supplement: S1 Text — (PDF) [file pone.0141304.s006.pdf]

Ray teeth identification

The ray teeth from Kennedy Coulee have a transverse crest on the occlusal surface of the crown that varies from barely present to strongly developed; in unworn teeth the crest is very well developed, with crenulations on the higher (lingual) half of the crown (S2E Figure). The sides of the crowns are straight and lack crenulations (S2F, S2H, S2J Figure). In the small hexagonal teeth, some roots are subequal in height to their crowns, while other roots are longer than their crowns; roots of the larger hexagonal teeth are distinctly longer than their crowns. The roots taper noticeably toward their tips. The nutritive groove of the root varies from arch shaped to keyhole shaped in side view. They cannot be *Myledaphus bipartitus* (sensu Kirkland et al., 2013) because the crenulations on the crowns of the Kennedy Coulee teeth do not cross the transverse crest, and do not have crenulated sides of the crowns.

Cristomylus cifellii and *Pseudomyledaphus madseni* teeth are both similar to the Kennedy Coulee teeth in having roots with tapering ends, and some teeth do have crenulations on the lingual side of the transverse crest of the crown (Kirkland et al., 2013). However, many *C. cifellii* small hexagonal teeth differ in the crowns being more oval shaped, with generally poorly defined sides of the crowns, whereas Kennedy Coulee small hexagonal teeth have well defined flat vertical sides of the crowns, similar to *P. madseni*. The midpoint of the root is wider in *C. cifellii* than in *P. madseni*; this character in the Kennedy Coulee teeth is more similar to that of *P. madseni*. Thus, the Kennedy Coulee teeth are more similar to *Pseudomyledaphus madseni* than to *Cristomylus cifellii*.

Kirkland et al. (2013) identified the "*Myledaphus* sp." teeth from the Foremost Formation of Alberta (Frampton, 2006; Peng et al., 2001) as being most similar to *Pseudomyledaphus madseni*; these Foremost Formation teeth may represent a distinct species, so Kirkland et al. (2013) referred them to *Pseudomyledaphus* sp. Because the Kennedy Coulee teeth are not perfectly consistent with *P. madseni*, but are morphologically consistent with the "*Myledaphus* sp." teeth of the Foremost and lower Oldman formations of Alberta (Brinkman et al., 2004; Peng et al., 2001), they are here referred to *Pseudomyledaphus* sp. as well, supporting the stratigraphic assignment of Kennedy Coulee to the upper Foremost Formation and lower Oldman Formation.

Two ray teeth were found in the MOR 1071 (locality JR-224) *Brachylophosaurus canadensis* bonebed in the Comrey Sandstone Zone of Malta, Montana (S2A-S2D Figure). These large hexagonal teeth have well-worn occlusal surfaces of the crowns with the faint remains of a transverse ridge and possibly very faint crenulations. Crenulations are present on all sides of the crown. The roots are longer than the crowns, and the nutrient grooves of the roots are keyhole shaped. These teeth are referable to the emended diagnosis of *Myledaphus bipartitus* by Kirkland et al. (2013). In Alberta, *Myledaphus bipartitus* is only found in the Comrey Sandstone Zone and higher units (Brinkman et al., 2004). The presence of these two stratigraphically separated ray taxa in Kennedy Coulee and Malta supports their stratigraphic assignments to Unit 1 and Unit 2 (Comrey Sandstone Zone), respectively, of the Oldman Formation.

References

Brinkman, D.B., Russell, A.P., Eberth, D.A. and Peng, J., 2004. Vertebrate palaeocommunities of the lower Judith River Group (Campanian) of southeastern Alberta, Canada, as

interpreted from vertebrate microfossil assemblages. Palaeogeography, Palaeoclimatology, Palaeoecology, 213(3-4): 295-313.
Frampton, E.K., 2006. Taphonomy and paleoecology of mixed invertebrate-vertebrate fossil assemblage in the Foremost Formation (Cretaceous, Campanian), Milk River Valley, Alberta, University of Calgary, Calgary, Alberta, 294 pp.
Kirkland, J.I., Eaton, J.G. and Brinkman, D.B., 2013. Elasmobranchs from Upper Cretaceous freshwater facies in Southern Utah. In: A.L. Titus and M.A. Loewen (Editors), At the Top of the Grand Staircase: the Late Cretaceous of southern Utah. Indiana University Press, Bloomington, Indiana, pp. 153-194.
Peng, J.-H., Russell, A.P. and Brinkman, D.B., 2001. Vertebrate microsite assemblages (exclusive of mammals) from the Foremost and Oldman Formations of the Judith River Group (Campanian) of Southeastern Alberta: an illustrated guide. Provincial Museum of Alberta Natural History Occasional Paper, 25: 1-54.
